# Massive dissemination of a SARS-CoV-2 Spike Y839 variant in Portugal

**DOI:** 10.1080/22221751.2020.1844552

**Published:** 2020-11-25

**Authors:** Vítor Borges, Joana Isidro, Helena Cortes-Martins, Sílvia Duarte, Luís Vieira, Ricardo Leite, Isabel Gordo, Constantino P. Caetano, Baltazar Nunes, Regina Sá, Ana Oliveira, Raquel Guiomar, Portuguese network for SARS-CoV-2 genomics, João Paulo Gomes

**Affiliations:** a Bioinformatics Unit, Department of Infectious Diseases, National Institute of Health Doutor Ricardo Jorge (INSA), Lisbon, Portugal; b Reference and Surveillance Unit, Department of Infectious Diseases, National Institute of Health Doutor Ricardo Jorge (INSA), Lisbon, Portugal; c Innovation and Technology Unit, Department of Human Genetics, National Institute of Health Doutor Ricardo Jorge (INSA), Lisbon, Portugal; d Centre for Toxicogenomics and Human Health (ToxOmics), Genetics, Oncology and Human Toxicology, Nova Medical School Faculdade de Ciências Médicas, Universidade Nova de Lisboa, Lisbon, Portugal; e Instituto Gulbenkian de Ciência (IGC), Oeiras, Portugal; f Department of Epidemiology, National Institute of Health Doutor Ricardo Jorge (INSA), Lisbon, Portugal; g Centro de Investigação em Saúde Pública, Escola Nacional de Saúde Pública, Universidade Nova de Lisboa, Lisbon, Portugal; h Public Health Unit, Primary Care Cluster of Baixo Vouga, Central Regional Health Administration, Aveiro, Portugal; i National Reference Laboratory for Influenza and other Respiratory Viruses, Department of Infectious Diseases, National Institute of Health Doutor Ricardo Jorge (INSA), Lisbon, Portugal

**Keywords:** SARS-CoV-2, COVID-19, genomic epidemiology, mutation, genetic variant, Spike, fusion peptide, D839Y

## Abstract

Genomic surveillance of SARS-CoV-2 was rapidly implemented in Portugal by the National Institute of Health in collaboration with a nationwide consortium of >50 hospitals/laboratories. Here, we track the geotemporal spread of a SARS-CoV-2 variant with a mutation (D839Y) in a potential host-interacting region involving the Spike fusion peptide, which is a target motif of anti-viral drugs that plays a key role in SARS-CoV-2 infectivity. The Spike Y839 variant was most likely imported from Italy in mid-late February and massively disseminated in Portugal during the early epidemic, becoming prevalent in the Northern and Central regions of Portugal where it represented 22% and 59% of the sampled genomes, respectively, by 30 April. Based on our high sequencing sampling during the early epidemics [15.5% (1275/8251) and 6.0% (1500/24987) of all confirmed cases until the end of March and April, respectively], we estimate that, between 14 March and 9 April (covering the epidemic exponential phase) the relative frequency of the Spike Y839 variant increased at a rate of 12.1% (6.1%–18.2%, CI 95%) every three days, being potentially associated with 24.8% (20.8–29.7%, CI 95%; 3177–4542 cases, CI 95%) of all COVID-19 cases in Portugal during this period. Our data supports population/epidemiological (founder) effects contributing to the Y839 variant superspread. The potential existence of selective advantage is also discussed, although experimental validation is required. Despite huge differences in genome sampling worldwide, SARS-CoV-2 Spike D839Y has been detected in 13 countries in four continents, supporting the need for close surveillance and functional assays of Spike variants.

## Introduction

The causative agent of COVID-19, the novel coronavirus SARS-CoV-2 (Severe Acute Respiratory Syndrome Coronavirus 2), is a tremendous global threat, already leading to nearly 25 million confirmed cases and approximately 800 thousand deaths worldwide, as of 30 August 2020 [1]. The Spike protein governs the binding of SARS-CoV-2 with its receptor angiotensin-converting enzyme 2 (ACE2) in human cells, the fusion between the viral and host cell membranes and, thus, the virus entry [2–8]. This protein, which decorates the virion surface, also induces neutralizing antibodies and is therefore the key target for vaccine development [2,5,9]. In this context, it is of upmost importance to track the genetic diversity and evolution of circulating SARS-CoV-2 at regional and global levels, with special focus on detecting the emergence and monitoring the spread of Spike variants. Although surveillance has been expectedly focused on genetic changes affecting the Spike receptor binding domain (RBD) [2,10–12], changes in other domains should also be closely surveyed, particularly when they are linked to variant frequency increase at local, regional or global levels [13,14]. A variant carrying the Spike D614G mutation stands out as a remarkable example, as it became dominant worldwide during the first months of the pandemic [13,15,16] with recent studies suggesting that the G614 variant might be linked to an increased transmissibility but not pathogenicity [13]. Spike amino acid 614 is pocketed adjacent to the fusion peptide, which is the functional fusogenic element of the Spike protein [2], and near the expected cleavage site, suggesting that G614 might have induced a conformational change influencing the dynamics of the spatially proximal fusion peptide, thereby resulting in the altered infectivity [13]. Hence, other mutations of interest outside the RBD have been highlighted [13], particularly those falling within the Spike fusion peptide or proximal regions [2,13], due to the critical role of these motifs in inserting SARS-CoV-2 into the membrane of human cells [2,17]. The fusion mechanism is also pointed out as an important target for the development of specific drugs against coronavirus, since it is expectedly less mutable than the surface-exposed and immunogenic RBD [2,18]. The Spike amino acid 839, within the fusion peptide or proximal regions (there is still no consensus on their precise location) [2], is being highlighted due to its potential specific host-interacting role in Spike cleavage for SARS-CoV-2 fusion activation and/or in the induction of host inflammatory responses [13,19,20].

Here, we evaluated the temporal and geographical spread of a SARS-CoV-2 variant carrying the Spike protein amino acid change D839Y that had a massive dissemination during the early COVID-19 epidemic in Portugal after its introduction from Italy in mid-late February. After the globally dispersed Spike D614G mutation, this is the first study reporting the superspread of a Spike variant with a tremendous epidemiological impact at country level.

## Material and methods

A detailed description of methods is available as Supplementary material.

### Sample characterization

Samples used in this study were collected as part of the ongoing national SARS-CoV-2 laboratory surveillance conducted by the National Institute of Health (INSA) Doutor Ricardo Jorge, Portugal. SARS-CoV-2 positive samples (either clinical specimens or RNA) were provided by a nationwide network with >50 laboratories that was established at the beginning of the epidemic in Portugal. Available demographic information, date of sample collection, date of illness onset and travel history were provided by laboratories and Regional and National Health Authorities. Geographical data presented in this study refers to the Region (“Health Administration region”), District or Municipality of the patients’ residence or, when no information is available (for a small proportion of cases), to the location of exposure or of the hospital/laboratory that collected/sent the sample.

### SARS-CoV-2 genome sequencing and analysis

After cDNA synthesis, SARS-CoV-2 positive RNA samples were subjected to amplicon-based whole-genome amplification with tiled, multiplexed primers [21], following the Artic Consortium protocol (https://artic.network/ncov-2019; https://www.protocols.io/view/ncov-2019-sequencing-protocol-bbmuik6w). After Illumina NexteraXT library preparation, paired-end sequencing was performed either on Illumina MiSeq or NextSeq 550, targeting ∼1M reads per sample.

Analysis of sequence read data was conducted using the bioinformatics pipeline implemented in INSaFLU (https://insaflu.insa.pt/; https://github.com/INSaFLU), which is a web-based (and also locally installable) platform for amplicon-based next-generation sequencing data analysis [22].

### Phylogenetic analysis and real-time data sharing on SARS-CoV-2 genetic diversity and geotemporal spread in Portugal

A total of 1516 SARS-CoV-2 genome sequences were analyzed in this study (corresponding to INSA’s collection as of 23 July 2020; Table S1) using the SARS-CoV-2 Nextstrain pipeline [23] version from March 23, 2020 (https://github.com/nextstrain/ncov).

A website (https://insaflu.insa.pt/covid19) was launched on March 28, 2020 for real-time data sharing on SARS-CoV-2 genetic diversity and geotemporal spread in Portugal, giving access to “situation reports” of the study and providing interactive data navigation using both Nextstrain (https://nextstrain.org/) [23] and Microreact (https://microreact.org/) [24] tools. To explore the frequency of Spike D839Y variant at worldwide level, we downloaded all the amino acid sequences (and associated metadata) of SARS-CoV-2 spike protein available at GISAID (as of 23 July 2020). The genome sequences with the D839Y mutation detected abroad were downloaded from GISAD (Table S2) and subjected to clade classification and integration into “global” and Portugal phylogeny using Nextstrain (https://nextstrain.org/ncov) and Nextclade (https://clades.nextstrain.org/).

### Statistical analysis

In order to assess the temporal variation in the proportion of D839Y mutation among sequenced samples, a binomial regression model with logarithmic link function was applied. The model was then applied to extrapolate the evolution of Y839 cases in the total case population (data presented in Figure 4). To increase the robustness of the analysis, the studied timeframe (from 14 March to 9 April, overlapping the exponential phase of the epidemic in Portugal) was adjusted to ensure 3-days bins with at least 25 genome sequences. We assumed one day as the timeframe delay between sample collection and case notification. The Kruskal-Walls non-parametric test was used to assess the existence of statistically significant differences in Ct values between groups (Figure S4). Differences in Ct values for each pair of groups were assessed using the Wilcoxon test adjusted for multiple comparison tests.

## Results

### Introduction and spread of the SARS-CoV-2 Spike Y839 variant in Portugal

Acting as the National Reference Laboratory for SARS-CoV-2, INSA rapidly established the genome-based molecular surveillance of SARS-CoV-2 in Portugal. A website (https://insaflu.insa.pt/covid19) was launched, providing updated data regarding the analysis of the SARS-CoV-2 genetic diversity and geotemporal spread. As of July 23, 2020, INSA had analysed 1516 genome sequences (Table S1), enrolling 15.5% (1275/8251) and 6.0% (1500/24987) of all confirmed cases detected until the end of March and April, respectively. According to Nextstrain clade definition (https://clades.nextstrain.org/), the 1516 SARS-CoV2 genomes from Portugal (https://microreact.org/project/nDGsJKFv7gQTj1q8CQwwKR/18a0a470) follow, in general, the same trend observed at European level (https://nextstrain.org/ncov/europe) [15,25]. Most viruses (89.8%) integrate the phylogenetic branch enrolling clades 20A (40.8%), 20B (46.1%) and 20C (2.9%), carrying, among other genetic markers, the D614G amino acid replacement in the Spike protein [13,15,16]. Clades 19A and 19B were found at the relative frequencies of 7.4% and 2.8%, respectively.

**Figure 1.**
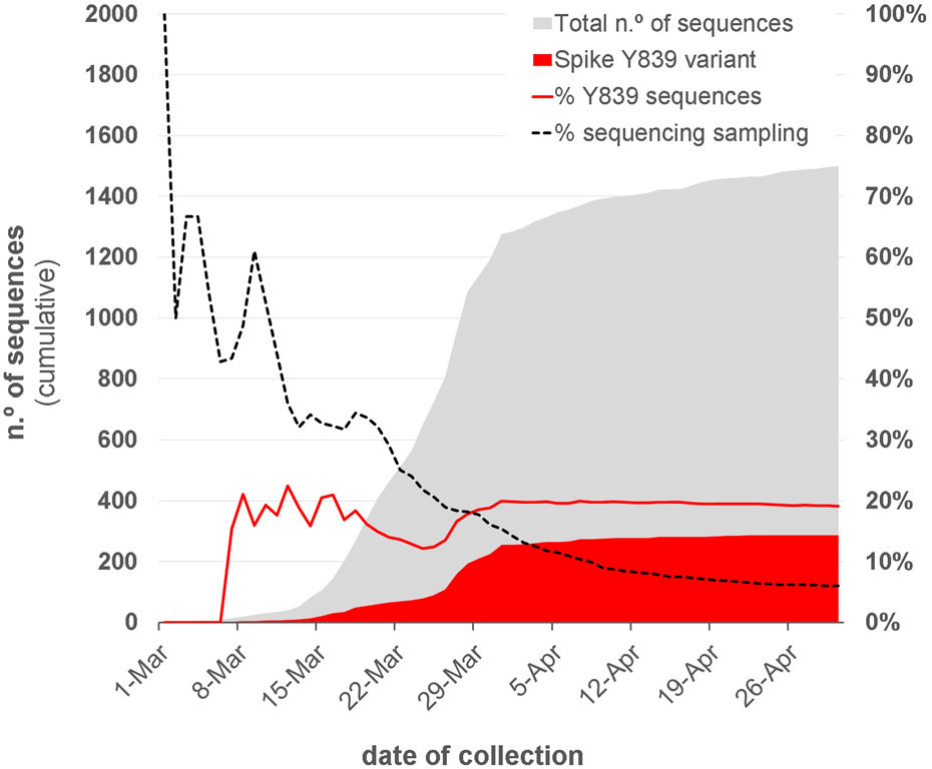
Overview of the SARS-CoV-2 genome sequencing sampling in Portugal and cumulative relative frequency of the circulating Spike Y839 variant, as of 30 April 2020 (*n* = 1500). Area plots (left y-axis) reflect the cumulative total number of SARS-CoV-2 genome sequences (gray) and Spike Y839 variant sequences (red) obtained in Portugal during the first two months of the epidemic. Lines (right y-axis) display the cumulative percentage of COVID-19 confirmed cases for which SARS-CoV-2 genome data was generated (“sequencing sampling” – black dash line) and the cumulative proportion of the Spike Y839 variant sequences (red line) detected in Portugal during the same period.

**Figure 2.**
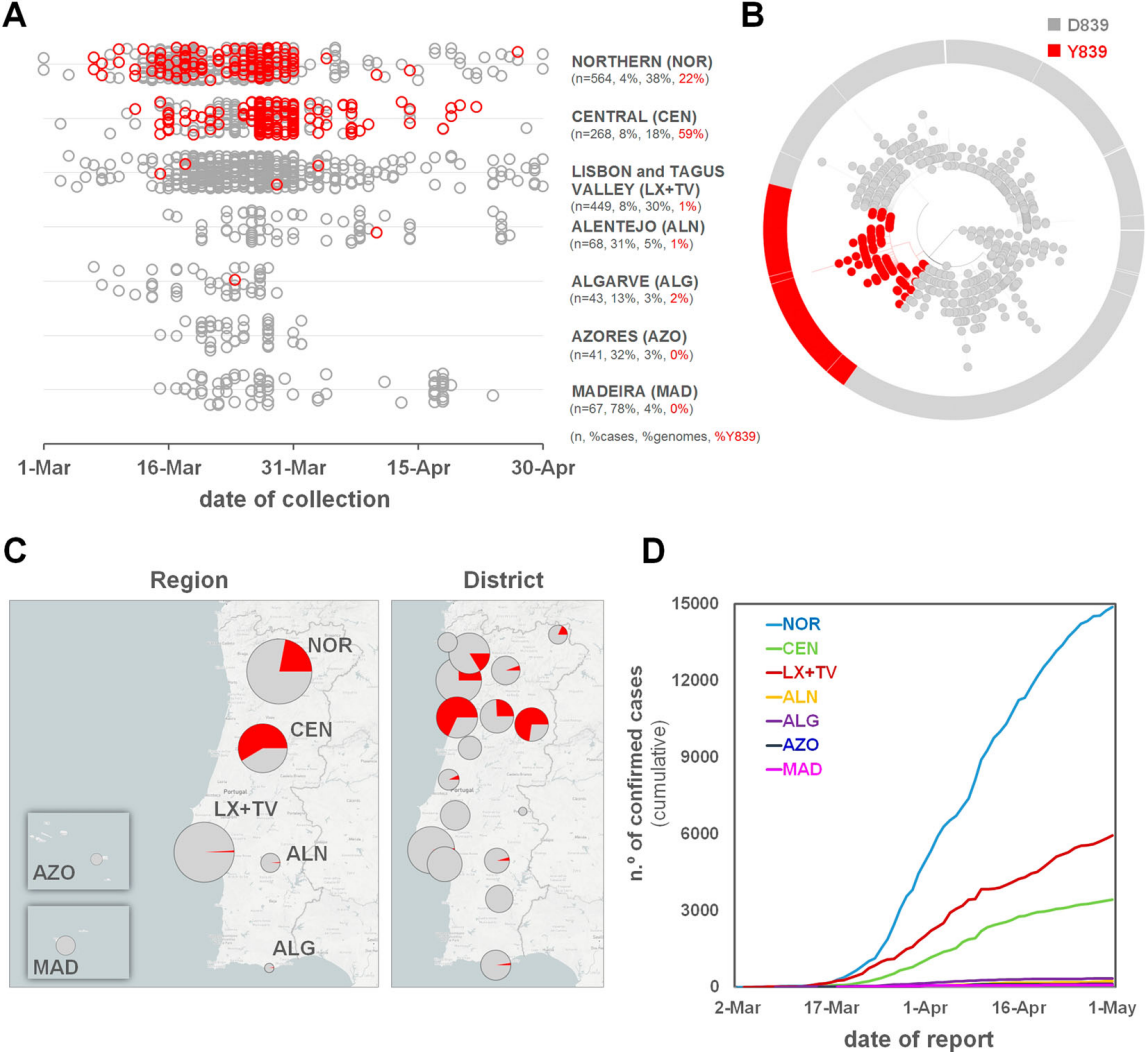
Landscape of the geotemporal spread of SARS-CoV-2 Spike Y839 variant in Portugal by region, as of 30 April 2020. (A) Distribution of the analysed genome sequences (*n* = 1500) by date of sample collection and Health Administration region, highlighting COVID-19 cases caused by the Spike Y839 variant (red dots). In y-axis, it is indicated, for each region, the number of sequences analysed (*n*), the percentage of confirmed cases with SARS-CoV-2 genome data (% cases), the percentage of sequences from each region in the whole dataset (% genomes) and the percentage of Y839 variant sequences (%Y839, in red), as of 30 April, 2020. (B) Radial maximum likelihood phylogenetic tree showing the high proportion of genomes with the Spike D839Y mutation detected in Portugal [about 20% of all sequences collected until the end of March (255/1275) or the end of April (287/1500)]. This dataset covers 15.5% (1275/8251) and 6.0% (1500/24987) of all confirmed cases detected until the end of March and April, respectively. The phylogeny and geotemporal distribution can be visualized interactively at https://microreact.org/project/nDGsJKFv7gQTj1q8CQwwKR/0489f840 (geographic resolution by Region) and https://microreact.org/project/2kh3TRVYB9gWGRpNSJWDW5/b6c659e0 (geographic resolution by District) using Microreact (https://microreact.org/). (C) Distribution of the Spike Y839 variant by Health Administration region, highlighting its high relative frequency in the Northern and Central regions of Portugal, where this variant represented 22% and 59% of the sampled genomes until the end of April 2020, respectively. The size of the pie charts is proportional to the number of sequenced genomes. (D) Cumulative total number of COVID-19 confirmed cases by Health Administration region, showing the Northern region as the “epicenter” of the epidemic during the two first months (source: General Directorate of Health (DGS), https://covid19.min-saude.pt/relatorio-de-situacao/).

**Figure 3.**
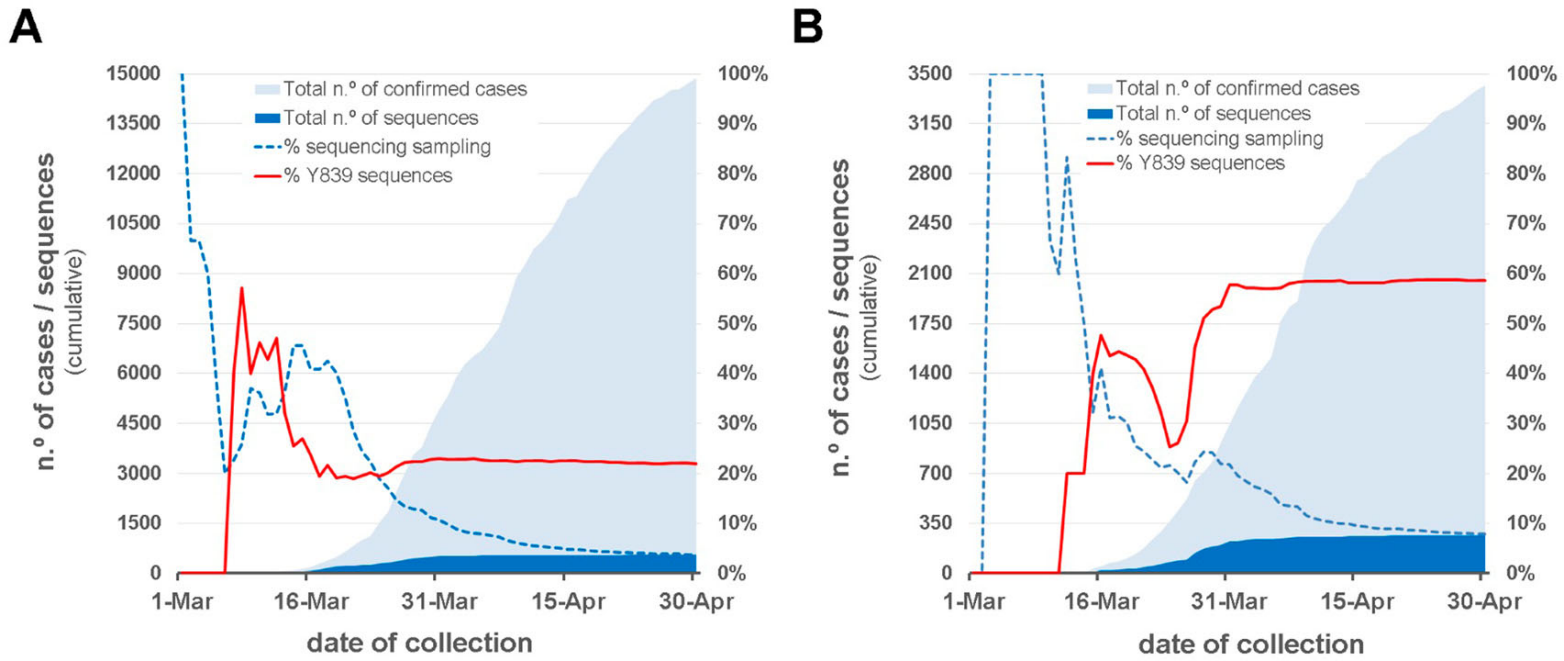
Overview of the SARS-CoV-2 genome sequencing sampling and cumulative relative frequency of the circulating Spike Y839 variant, as of 30 April 2020 (*n* = 1500), in the Northern (A) and Central (B) regions of Portugal. Area plots (left y-axis) reflect the cumulative total number of COVID-19 confirmed cases (light blue) and SARS-CoV-2 genome sequences (dark blue) detected/generated in each Health Administration region. Lines (right y-axis) display the cumulative percentage of COVID-19 confirmed cases with SARS-CoV-2 genome data, i.e. sequencing sampling (blue dash line) and the cumulative proportion of the Spike Y839 variant sequences (red line) detected in those regions during the same period.

**Figure 4.**
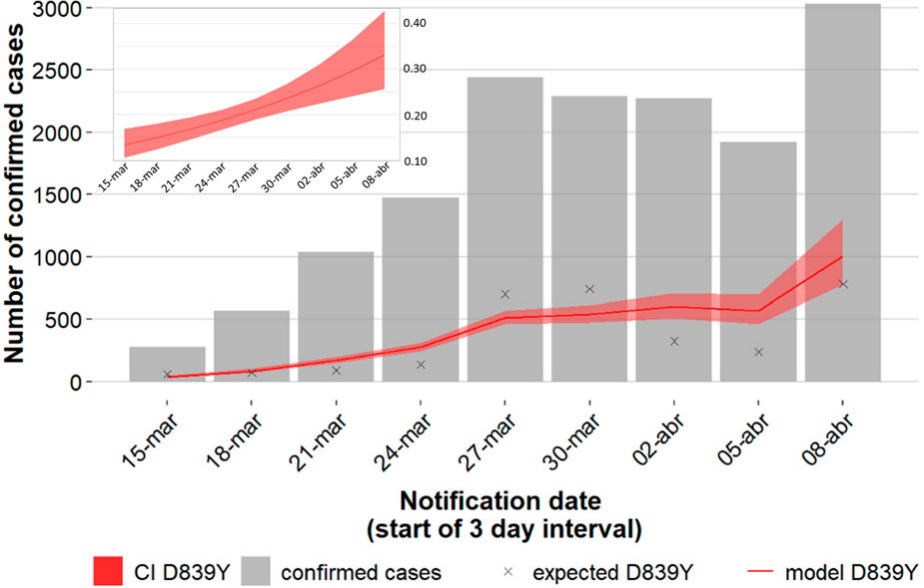
Increasing trajectory of the Spike Y839 variant and estimated weight of this variant in the total number of COVID-19 confirmed cases in early epidemic. A binomial regression model with logarithmic link function was applied to assess the temporal variation in the proportion of the Y839 variant among sequenced samples (graph in the upper left corner, showing an estimate increase from 13.3% to 33.1%). This model was then applied to extrapolate the evolution of Y839 cases (red line) in the total confirmed case population (gray bars) at each 3-day interval (main graph). Crosses represent the estimated Y839 cases and the shaded region shows the 95% confidence interval. 1-day was assumed as the timeframe delay between sample collection and case notification.

Within clade 20A, a SARS-CoV-2 variant carrying the Spike amino acid change D839Y (due to a G24077 T SNP) was detected early (7 March 2020) during the COVID-19 epidemic in Portugal. The Spike Y839 variant was most likely imported from Italy in mid-late February 2020, as first detected genomes were all collected from individuals from the Northern region of Portugal that contacted (primarily or secondarily) with epidemiologically linked infected individuals that had been in Milan for an international trade fair during the third week of February 2020. Concordantly, the first D839Y genome sequence reported worldwide was collected in Italy (Lombardy) on 21 February (Italy/PV-5314-N/2020; GISAID accession number EPI_ISL_451307) (Table S2) [26], being identical (i.e, Nextstrain clade 20A background plus the G24077 T SNP) to the “founder” Spike Y839 variant genome sequences detected in Portugal (https://insaflu.insa.pt/covid19). So far, current data and ongoing epidemiological investigations support a single origin (Milan, Italy) and, although there was a substantial representation of Portuguese companies in this event, available data links the introduction of the Y839 variant in Portugal to a single industrial area in the North of Portugal. The Spike Y839 variant became particularly prevalent in Portugal, representing about 20% of all sampled genomes collected until the end of March (255/1275) or the end of April (287/1500) (Figure 1). Its circulation was particularly marked in Northern (epicenter of COVID-19 epidemic in Portugal) and Central regions of Portugal, representing 22% and 59% of the sampled genomes, respectively, by the end of April 2020 (Figures 2 and 3; https://microreact.org/project/nDGsJKFv7gQTj1q8CQwwKR/0489f840). In the same period, four districts (with at least 50 genomes sampled as of 30 April) revealed the highest Y839 relative frequency: Braga (16.3%), Porto (25.2%), Aveiro (68.1%) and Guarda (72.7%) (Figure S1). We estimate that the relative frequency of Y839 increased at a rate of 12.1% (6.1%–18.2%, CI 95%) every three days between 14 March and 9 April, increasing from 13.3% to 33.1% (Figure 4). It was potentially associated with 3793 (3177–4542, CI 95%) COVID-19 cases in Portugal during that period, representing 24.8% (20.8–29.7%, CI 95%) of the total confirmed cases reported in the same period (Figure 4). Hence, our data supports that the Spike Y839 variant was circulating in Portugal since mid-late February (more than one week before the first COVID-19 confirmed case at 2 March 2020), being most likely responsible for the largest SARS-CoV-2 transmission chain occurred during the first 1–1.5 months of COVID-19 epidemic in Portugal. In particular, the Spike Y839 variant is strongly linked to a large and worrying COVID-19 “local” outbreak occurring in a small municipality (Ovar) in the coastal side of the Central region of the country (District of Aveiro), with 80% of the genomes collected from this municipality carrying the Y839 variant. Ovar was the only municipality in Portugal mainland that was subjected to strict local quarantine and lockdown measures (from 17 March to 17 April), presenting an incidence of 636 cases per 100 000 inhabitants in the last 14 days by 5 April. The Public Health Unit of Primary Care Cluster of Baixo Vouga (covering several municipalities, including Ovar), has been carrying out a deep investigation leading to the identification of clusters of potential epidemiologically linked confirmed cases (“epiclusters”) among 1556 monitored COVID-19 cases (by 30 April). Our SARS-CoV-2 genome collection (as of 30 April) includes samples representative of 41 of those potential epiclusters (covering a total of 420 confirmed cases), of which 33 (323 confirmed cases, 77%) are exclusively associated with the Spike Y839 variant (Figure S2). In a conservative manner, it is reasonable to extrapolate that the Spike Y839 variant is potentially associated with about 1200 cases (77% of the total 1556 monitored cases) in the region covered by Primary Care Cluster of Baixo Vouga. Still, the Spike Y839 variant disseminated far beyond the coastal municipality of Ovar and neighbourhood municipalities. In the inland region of the country, Y839 variant was for instance linked to a large cluster of infected individuals living/working in a nursing home in Vila Nova de Foz Côa municipality (District of Guarda) detected by the end of March, and more than 50% of Y839 genomes detected in April were collected in the District of Viseu (Figure S1 and S3; https://microreact.org/project/2kh3TRVYB9gWGRpNSJWDW5/b6c659e0), which borders the district of Aveiro. In total, the highly prevalent Spike Y839 variant was already detected in 44 municipalities across 11 out of the 18 Districts of Portugal mainland, consolidating that this descendent variant of the globally spread G614 variant had a remarkable weight in the early COVID-19 epidemic in Portugal.

### Detection and circulation of SARS-CoV-2 Spike 839 amino acid variants worldwide

**Figure 5.**
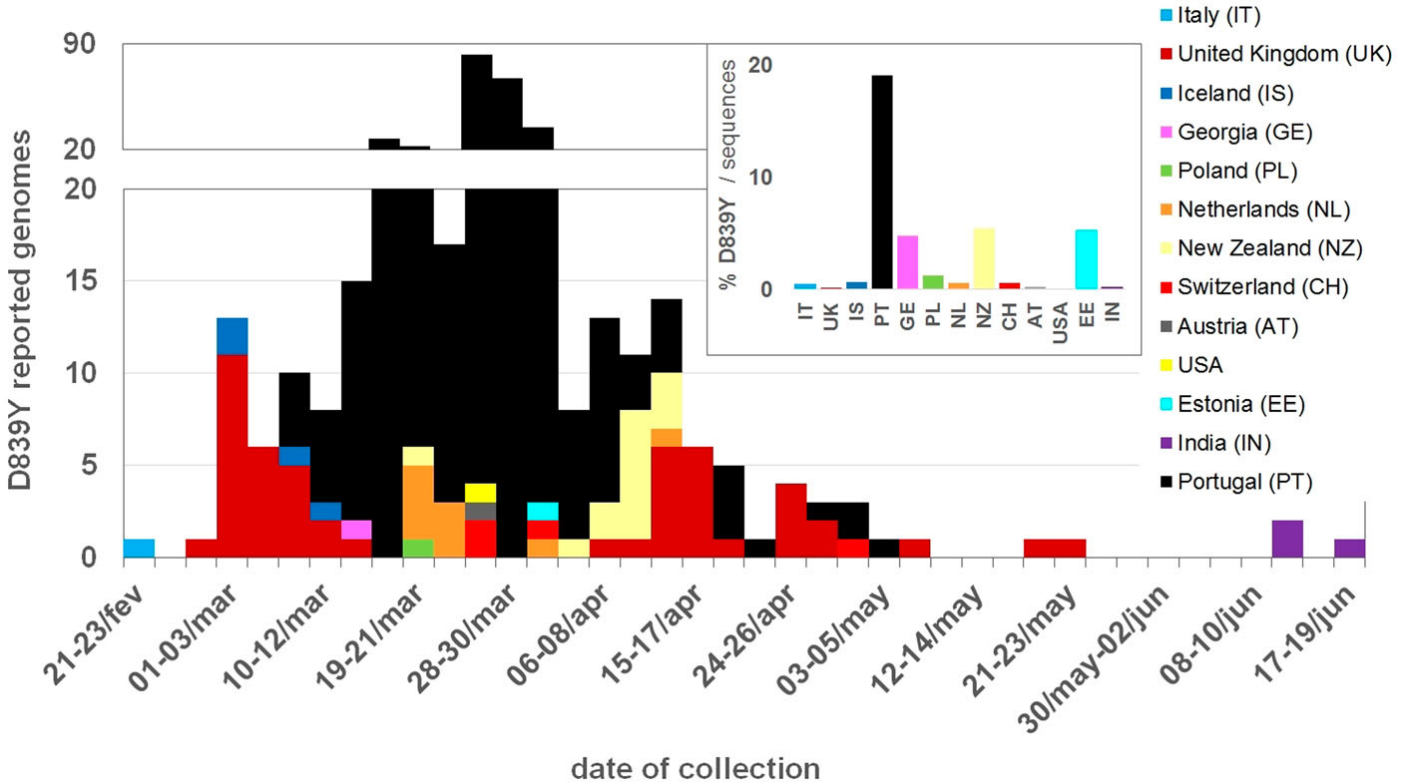
Detection and circulation of the SARS-CoV-2 Spike D839Y mutation worldwide. SARS-CoV-2 Spike amino acid sequences available at GISAID (https://www.gisaid.org/), as of 23 July 2020, were download, aligned and screened for the presence of mutations in Spike 839 amino acid position. The main plot displays the country and data of collection of 92 Spike sequences with the D839Y mutation (detailed in Table S2). The bar graph in the upper right corner displays the proportion of D839Y sequences in the total number of Spike amino acid sequences available per country. As detailed in Table S2, the D839Y sequence from Estonia indicates March 2020 as the date of collection (31 March 2020 was assumed in this plot). Two genomes (one from United Kingdom and another from India) only had the year of sampling available, thus they were not included in the graph.

To explore the frequency of SARS-CoV-2 D839Y mutation (and other mutations in 839 protein position) at worldwide level, we downloaded 66548 amino acid sequences (and associated metadata) of SARS-CoV-2 Spike protein available at GISAID (as of 23 July 2020). From the 65367 sequences collected outside Portugal having data for the 839 position, 97 were found to display mutations in this amino acid of interest (Table 1, Table S2). Of those, 92 genomes revealed the same G24077T nucleotide substitution (leading to the D839Y amino acid replacement) in a SARS-CoV-2 with Spike G614 background. As previously noticed^13^, this data sustains that the global frequency of amino acid variants in this 839 site remains around 0.5% (similar estimates can be found in https://cov.lanl.gov/content/index and https://bigd.big.ac.cn/ncov/). After the first Spike Y839 variant was detected in Italy (Lombardy) on 21 February, the mutation D839Y has been reported in 12 other countries from four continents (Europe, Oceania, Asia and America) (Figure 5). Despite the highly unequal sequencing “sampling” (i.e. proportion of confirmed cases with SARS-CoV-2 genome data) between the different countries, it is noteworthy that, apart from Portugal, D839Y genomes represent ∼5% of all sequences made available at GISAID (as of 23 July 2020) by three countries (Estonia, Georgia and New Zealand) (Figure 5). Of note, the four genomes detected in Iceland on early March are also associated with travel history to Italy [15,27] (Table S2). Notwithstanding, fine-tune integration of the 92 Spike Y839 genomes detected abroad in the “global” phylogeny (using Nextstrain https://nextstrain.org/ncov and Nextclade https://clades.nextstrain.org/) pointed out that the D839Y amino acid change likely emerged independently in two other instances. This observation is supported by one genome sequence collected in the United Kingdom (Wales) on 24 April (Wales/PHWC-35B01/2020; GISAID accession EPI_ISL_474528), which presents all clade-defining SNPs of Nextstrain Clade 20C plus four additional SNPs (including G24077 T). In this particular case, we cannot exclude the hypothesis that the G24077 T nucleotide change might have been introduced in a clade “20C” SARS-CoV-2 by recombination, as “20C” and “20A harbouring Y839” viruses co-circulated in Wales during the collection period (13 out of the 51 Spike 839Y genomes from UK were collected in Wales between 10 April and 25 May). The potential third independent emergence of a Spike Y839 variant is supported by the four genomes collected in India, as they cluster apart from other G614+Y839 genomes, forming a sub-branch (supported by 6 SNPs, including G24077T) within a large cluster mostly enrolling genomes from India.

**Table 1.** Overview of the SARS-CoV-2 Spike amino acid sequences with mutations in the 839 site available at GISAID, as of 23 July 2020.

Spike mutation	D614G background	Count	Countries^a^^,^^b^	Date of collection range^b^
D839Y	G614	382	Italy (1), United Kingdom (51), Iceland (4), Portugal (290), Georgia (1), Poland (1), Netherlands (9), New Zealand (14), Switzerland (4), Austria (1), USA (1), Estonia (1), India (4)	21/Feb–17/Jun
D839E	G614	1	Netherlands (1)	28/Feb
D839N	G614	1	Australia (1)	20/Jun
D839N	D614	1	United Kingdom (1)	25/Mar
D839G	D614	2	United Kingdom (2)	24/Mar–8/May

^a^ Countries are ordered by the date of collection of the first reported genome with the Spike 839 site variant. Individual sequences are detailed in Table S1 (Portugal) and S2 (abroad).

^b^ The reported case in Estonia has March 2020 as the date of collection (31 March 2020 was assumed in the Figure 5). Two genomes (one from United Kingdom and another from India) only had the year of sampling available. These were not included in Figure 5.

### Impact of Spike Y839 variant on viral load

The Cycle threshold (Ct) obtained in diagnostic PCR is an indirect indicator for relative viral loads *in vivo*, with lower Ct values indicating higher viral loads. Increasing reports have been linking the globally dispersed Spike G614 variant to lower Ct values [13,28,29], suggestive of higher upper respiratory tract viral loads leading to increased G614 transmissibility, but not pathogenicity [13]. INSA gathered Ct values of 940 out of the 1516 genomes analyzed in present study. Despite the expected bias associated with sample collection (e.g. momentum), sampling (e.g. selection) and testing (e.g. extraction, PCR protocol and equipment) within datasets enrolling multiple laboratories, we sought to verify whether the same trend is observed for the D614/G614 comparison and whether the Spike Y839 variant (Spike background G614+Y839) may also be potentially linked with changes in Ct values. As previously seen [13,28,29], we observed that samples with Spike G614 had Ct values (mean = 22.2; *n* = 844) lower than those with D614 viruses (mean = 22.9; *n* = 96), although without statistical significance (Figure S4). Considering the phylogeny of SARS-CoV-2 in Portugal, we then compared Ct values according to D614/G614 status, phylogenetic group within G614 (i.e. 20B or non-20B) and D839/Y839 status (Figure S4; https://microreact.org/project/nDGsJKFv7gQTj1q8CQwwKR/f46f1fa4). Curiously, 20B clade (defined by the GGA-to-AAC SNP triplet at genome position 28881-3) revealed the lowest Ct values (mean = 21.7; *n* = 413) among all groups, although without statistical significance in pairwise comparisons. Regarding the D839/Y839 comparison, we observed that Y839 presented non-significant higher Ct values (mean = 22.7; *n* = 220) than the ancestral D839 (mean = 22.1; *n* = 720). However, considering that D839 includes both 20B and non-20B samples, contrarily to Y839, which is a sub-group within 20A (Figure S4), we repeated the analysis by excluding 20B samples from the comparison and observed similar average Ct values in D839 (mean = 22.6; *n* = 624) and Y839 (mean = 22.7; *n* = 220) groups. When applying the same rationale (i.e. excluding 20B) to the D614/G614 comparison, the 0.7 difference observed in the mean Ct values using the whole dataset decreased to less than 0.3.

## Discussion

One of the main objectives of conducting genome-based surveillance of circulating pathogens is to identify mutations potentially leading to fitness advantages and/or immunological/drug resistance. In this perspective, mutations in the SARS-CoV-2 Spike D839 site, which is predicted to fall within the Spike fusion peptide or proximal regions [2,13], are of particular interest given both its non-negligible relative frequency worldwide and the pivotal role of this protein region in inserting SARS-CoV-2 into human cell membranes [2,13]. In the present study, we show that a SARS-CoV-2 variant with a Spike D839Y mutation was associated with ∼25% of all COVID-19 cases in Portugal during the exponential phase of the epidemic, after its importation from Italy in mid-late February 2020. Our data shows that it was circulating in Portugal before the first COVID-19 confirmed case was detected on 2 March, becoming notably prevalent in the Northern (22%) and Central (59%) regions of the country by the end of April 2020 (Figures 2 and 3).

The massive dissemination of the Spike Y839 variant in Portugal might be due to a populational/epidemiological effect (founder effect). This variant was likely one of the first SARS-CoV-2 to be introduced in Portugal, so it might have had more opportunity to spread. Its introduction is strongly linked to an international trade fair in Milan with many Portuguese attendees. So, we can raise the possibility of several “simultaneous” introductions of Y839 variant in Portugal, although this scenario would likely imply its circulation in the South of Portugal and abroad, namely in Spain (also highly represented in the event) where it has not been detected so far (http://seqcovid.csic.es/nextspain/, as of 30 August) despite the high sequencing sampling. The alternative scenario of a single introduction is then plausible considering that travel history and contact tracing data collected to date links all initial cases to a single industrial area in the North of Portugal and does not indicate additional introductions. Regardless of the scenario, the increasing frequency trajectory of Spike Y839 variant would not have been mitigated because this variant disseminated well before the first confirmed cases in Portugal, when contact tracing, broad testing, and strict lockdown measures were still not in place. For instance, on 9 March, case definition for a COVID-19 suspected case did not include individuals with acute respiratory infection, unless they required hospitalization, reported travel history or any contact with suspected or confirmed cases in the 14 days before symptoms onset (https://www.dgs.pt/directrizes-da-dgs/orientacoes-e-circulares-informativas/orientacao-n-002a2020-de-25012020-atualizada-a-250220201.aspx). In another perspective, one cannot rule out that the high dissemination could have also been driven by fitness increase mediated by the D839Y mutation, which would be consistent with its estimated frequency increase from 13.3% to 33.1% in a 4-week period. In this hypothesis, this mutation would have posed “advantageous” structural changes in the Spike protein with potential impact on SARS-CoV-2 infectivity and, consequently, on its transmissibility, as suggested for D614G [13]. While D614G was hypothesized to increase SARS-CoV-2 infectivity by influencing the dynamics of the spatially proximal fusion peptide [13], D839Y, which itself falls within functional fusogenic element of Spike[2,13], could also have shaped this motif towards a better fitted fusion of SARS-CoV-2 with human cells. Also, recent data based on computational modeling suggested that mutations in Spike D839 may strengthen the interaction between the virus and human T cells potentially influencing host inflammatory responses [in particular, when replacing the aspartic acid (D) to an aromatic tyrosine (Y)] [19], and may influence Spike cleavage by host proteases during SARS-CoV-2 fusion activation and entry [20]. Nevertheless, these clues about the potential impact of this mutation on SARS-CoV-2 transmissibility/pathogenicity require evidence through experimental validation to verify or rule out the fitness advantage hypothesis. Although we did not observe any association between Y839 and Ct values (viral load) in our dataset, this assessment needs to be revisited as more data is acquired and be complemented with other assays. Notwithstanding, we indirectly observed that samples from clade 20B had a lower mean Ct value than non-20B groups in our dataset, supporting that it is worth performing this comparison with additional datasets and that the evaluation of (sub)clade effects should not be neglected in this kind of screenings.

Besides the potential functional role of D839Y mutation and its high prevalence in Portugal, its detection in 12 other countries from four continents, it’s probable independent emergence in distinct times and genetic clades (20A and 20C) in some of these countries and its considerable relative weight (∼5%) in the sampled genomes of three countries (besides Portugal) also indicate that the hypothesis of selective advantage is not implausible. Contrarily to Spike G614 variant, which emerged way before the general quarantine in Europe, the Spike Y839 variant likely emerged on mid-late February in Lombardy, Italy. This is strongly corroborated by the detection of both D839 and Y839 subpopulations in different anatomical sites of the Y839-infected individual in Lombardy, Italy, by 21 February [26]. At this time, rigid lockdown measures started being implemented everywhere in Europe, which posed strong bottlenecks on SARS-CoV-2 population, likely giving Y839 variant less opportunity to expand, even if it was selectively advantageous over other circulating variants. Nonetheless, in Portugal, contrarily to what might have happened in other countries, the timeline of the epidemics certainly favoured a high weight of “founder effect” in the remarkable dissemination of the Y839 variant. For instance, first lockdowns in Lombardy, Italy, where Spike Y839 variant was firstly detected on 21 February, coincidently began on this date [30]. In contrast, in Portugal, first cases were confirmed in 2 March and national quarantine was implemented on 18 March, when Spike Y839 variant had already been circulating in the community for at least three weeks. It is still worth highlighting that the huge discrepancies in sequencing sampling between countries completely hampers a real knowledge of D839Y frequency regionally and globally. Our sequencing sampling after 30 April does not allow us to infer the current relative frequency of the Spike Y839 variant in Portugal. One can speculate that its circulation was highly contained considering that, after this period, the epidemic evolved favourably in Northern and Central regions (where Y839 had mostly circulated), contrarily to the Lisbon and Tagus Valley region (where Y839 was, at that time, rarely seen) (http://www.insa.min-saude.pt/wp-content/uploads/2020/08/Report_covid19_07_08_2020.pdf).

In summary, we describe the emergence and increase in frequency of a Spike Y839 variant that reached a tremendous impact on COVID-19 epidemic in Portugal, as estimated by its high relative weight of one in each four cases during the exponential phase of the epidemic. Similarly to other major Spike variants, Y839 certainly constitutes an important target for functional and immunological studies. Our study reinforces the need for continuous and close surveillance of SARS-CoV-2 genetic diversity, with emphasis on detecting and monitoring variants with potential impact at biological and/or epidemiological levels.

## Supplementary Material

Supplemental Material

Supplementary_Information.docx

## Data Availability

SARS-CoV-2 genome sequences generated in this study were uploaded to GISAID database (https://www.gisaid.org/). Accession numbers can be found in Supplementary material (Table S1).
