# Supplementary material for: Massive dissemination of a SARS-CoV-2 Spike Y839 variant in Portugal
Source: Emerg Microbes Infect. 2020 Nov 25;9(1):2488–96. doi: 10.1080/22221751.2020.1844552 (PMC7717510; doi:10.1080/22221751.2020.1844552)
Supplement: Supplemental Material [file TEMI_A_1844552_SM5824.docx]

**Supplementary Information**

**Authors list within the consortium entitled “Portuguese network for SARS-CoV-2 genomics”**

All members (listed below) of the consortium entitled “Portuguese network for SARS-CoV-2 genomics” are authors of this manuscript.

| Carlos Sousa | Laboratório De Biologia Molecular da Unilabs Portugal | [carlossousa@LAP.PT](mailto:carlossousa@LAP.PT) |
| --- | --- | --- |
| Pedro Cardoso | Laboratório De Biologia Molecular da Unilabs Portugal | [plnncardoso@gmail.com](mailto:plnncardoso@gmail.com) |
| Carlos Cardoso | Laboratório de Análises Clínicas Dr Joaquim Chaves | [carlos.cardoso@jcs.pt](mailto:carlos.cardoso@jcs.pt) |
| Laura Brum | Synlab Lisboa | laura.brum@synlab.com |
| Lurdes Monteiro | Secção de Patologia Molecular, Synlab Lisboa | [lurdes.monteiro@synlab.pt](mailto:lurdes.monteiro@synlab.pt) |
| Cristina Toscano | Laboratório de Microbiologia e Biologia Molecular do Centro Hospitalar de Lisboa Ocidental | [ctoscano@chlo.min-saude.pt](mailto:ctoscano@chlo.min-saude.pt) |
| Maria Ana Pessanha | Laboratório de Microbiologia e Biologia Molecular do Centro Hospitalar de Lisboa Ocidental | [mpessanha@chlo.min-saude.pt](mailto:mpessanha@chlo.min-saude.pt) |
| Ana Paula Dias | Laboratório de Microbiologia e Biologia Molecular do Centro Hospitalar de Lisboa Ocidental | [apdias@chlo.min-saude.pt](mailto:apdias@chlo.min-saude.pt) |
| João Dias | Laboratório de Microbiologia e Biologia Molecular do Centro Hospitalar de Lisboa Ocidental | [jepdias@chlo.min-saude.pt](mailto:jepdias@chlo.min-saude.pt) |
| Maria Helena Ramos | Serviço de Microbiologia, Centro Hospitalar do Porto | directora.crmad@chporto.min-saude.pt |
| Ana Constança | Serviço de Microbiologia, Centro Hospitalar do Porto | anaconstancamendes.microbiologia@chporto.min-saude.pt |
| Agostinho José S. Lira | Centro Hospitalar de Vila Nova de Gaia e Espinho | [lira@chvng.min-saude.pt](mailto:lira@chvng.min-saude.pt) |
| Filomena Lacerda | Centro Hospitalar de Vila Nova de Gaia e Espinho | [filomenalacerda@chvng.min-saude.pt](mailto:filomenalacerda@chvng.min-saude.pt) |
| Luis Marques Silva | Centro Hospitalar de Vila Nova de Gaia e Espinho | [luis.silva@chvng.min-saude.pt](mailto:luis.silva@chvng.min-saude.pt) |
| Maria Matos Figueiredo | Centro Hospitalar de Vila Nova de Gaia e Espinho | [maria.matos.figueiredo@chvng.min-saude.pt](mailto:maria.matos.figueiredo@chvng.min-saude.pt) |
| Nair Seixas | Centro Hospitalar de Vila Nova de Gaia e Espinho | [nair.seixas@chvng.min-saude.pt](mailto:nair.seixas@chvng.min-saude.pt) |
| Jorge Meneses | Centro Hospitalar de Vila Nova de Gaia e Espinho | [jorge.meneses@chvng.min-saude.pt](mailto:jorge.meneses@chvng.min-saude.pt) |
| Paulo Leandro | Centro Hospitalar de Vila Nova de Gaia e Espinho | [paulo.leandro@chvng.min-saude.pt](mailto:paulo.leandro@chvng.min-saude.pt) |
| Alexandra Estrada | Serviço de Patologia Clínica, Hospital de Braga | [alexandra.estrada@hb.min-saude.pt](mailto:alexandra.estrada@hb.min-saude.pt) |
| Fernando Branca | Serviço de Patologia Clínica, Hospital de Braga | [fernando.branca@hb.min-saude.pt](mailto:fernando.branca@hb.min-saude.pt) |
| Aida M. Sousa Fernandes | Laboratório Regional de Saúde Pública Drª Laura Ayres - ARS Algarve | [afernandes@arsalgarve.min-saude.pt](mailto:afernandes@arsalgarve.min-saude.pt) |
| Aurora Direito | Serviço de Patologia Clinica - Unidade Local de Saúde Litoral Alentejano | [aurora.direito@ulsla.min-saúde.pt](mailto:aurora.direito@ulsla.min-saúde.pt) |
| Inna Slobidnyk | Serviço de Patologia Clinica - Unidade Local de Saúde Litoral Alentejano | inna.slobidnyk@ulsla.min-saude.pt |
| Maria João Peres | Laboratório de Imunologia e Biologia Molecular, Centro Hospitalar de Setúbal | [maria.peres@chs.min-saude.pt](mailto:maria.peres@chs.min-saude.pt) |
| Rita Côrte-Real | Laboratório Biologia Molecular- Serviço de Patologia Clínica,Centro Hospitalar Universitário Lisboa Central | [rita.cortereal@chlc.min-saude.pt](mailto:rita.cortereal@chlc.min-saude.pt) |
| Madalena Almeida Santos | Laboratório Biologia Molecular- Serviço de Patologia Clínica,Centro Hospitalar Universitário Lisboa Central | [santos22@chlc.min-saude.pt](mailto:santos22@chlc.min-saude.pt) |
| Olga Costa | Laboratório Biologia Molecular- Serviço de Patologia Clínica,Centro Hospitalar Universitário Lisboa Central | [ompena@netcabo.pt](mailto:ompena@netcabo.pt) |
| Conceição Godinho | Laboratório Biologia Molecular- Serviço de Patologia Clínica,Centro Hospitalar Universitário Lisboa Central | [mclopes@chlc.min-saude.pt](mailto:mclopes@chlc.min-saude.pt) |
| Paula Branquinho | Laboratório Biologia Molecular- Serviço de Patologia Clínica,Centro Hospitalar Universitário Lisboa Central | [biomolecular@sapo.pt](mailto:biomolecular@sapo.pt) |
| Lurdes Lopes | Laboratório Biologia Molecular- Serviço de Patologia Clínica,Centro Hospitalar Universitário Lisboa Central | biomolecular@sapo.pt |
| Paula Soares | Laboratório Biologia Molecular- Serviço de Patologia Clínica,Centro Hospitalar Universitário Lisboa Central | biomolecular@sapo.pt |
| Lidia Santos | Laboratório Biologia Molecular- Serviço de Patologia Clínica,Centro Hospitalar Universitário Lisboa Central | biomolecular@sapo.pt |
| Patricia Miguel | Laboratório Biologia Molecular- Serviço de Patologia Clínica,Centro Hospitalar Universitário Lisboa Central | biomolecular@sapo.pt |
| Isabel Dias | Laboratório Biologia Molecular- Serviço de Patologia Clínica,Centro Hospitalar Universitário Lisboa Central | biomolecular@chlc.min-saude.pt |
| Isabel Fernandes | Laboratório Biologia Molecular- Serviço de Patologia Clínica,Centro Hospitalar Universitário Lisboa Central | biomolecular@chlc.min-saude.pt |
| Sónia Rodrigues | Laboratório Biologia Molecular- Serviço de Patologia Clínica,Centro Hospitalar Universitário Lisboa Central | biomolecular@chlc.min-saude.pt |
| Fátima Vale | Serviço de Patologia Clínica, Unidade Local de Saúde da Guarda | fatima.vale@ulsguarda.min-saude.pt |
| Joana Ramos | Serviço de Patologia Clínica, Unidade Local de Saúde da Guarda | [joana.ramos@ulsguarda.min-saude.pt](mailto:joana.ramos@ulsguarda.min-saude.pt) |
| Rita Gralha | Serviço de Patologia Clínica, Unidade Local de Saúde da Guarda | [rita.gralha@ulsguarda.min-saude.pt](mailto:rita.gralha@ulsguarda.min-saude.pt) |
| Patricia Fonseca | Serviço de Patologia Clínica, Unidade Local de Saúde da Guarda | [patricia.fonseca@ulsguarda.min-saude.pt](mailto:patricia.fonseca@ulsguarda.min-saude.pt) |
| Nelson Ventura | Serviço de Patologia Clínica, Unidade Local de Saúde da Guarda | [nelson.ventura@ulsguarda.min-saude.pt](mailto:nelson.ventura@ulsguarda.min-saude.pt) |
| Filomena Caldeira | Hospital Espírito Santo, Évora | [dir.patcli@hevora.min-saude.pt](mailto:dir.patcli@hevora.min-saude.pt) |
| Margarida Farinha | Serviço de Patologia Clínica, Centro Hospitalar Tondela-Viseu | [lab.patclinica@hstviseu.min-saude.pt](mailto:lab.patclinica@hstviseu.min-saude.pt) |
| Ana Caldas | Serviço de Patologia Clínica, Centro Hospitalar Tondela-Viseu | [8493@hstviseu.min-saude.pt](mailto:8493@hstviseu.min-saude.pt) |
| Carina de Fátima Rodrigues | Centro de Investigação de Montanha, Instituto Politécnico de Bragança | carina@ipb.pt |
| Maria Alice Pinto | Centro de Investigação de Montanha, Instituto Politécnico de Bragança | [apinto@ipb.pt](mailto:apinto@ipb.pt) |
| António Albuquerque | Unidade Local de Saúde de Matosinhos | [antonio_albuquerque82@hotmail.com](mailto:antonio_albuquerque82@hotmail.com) |
| Valquíria Alves | Unidade Local de Saúde de Matosinhos | [valquiria.alves@ulsm.min-saude.pt](mailto:valquiria.alves@ulsm.min-saude.pt) |
| João Carlos Sousa | Life and Health Sciences Research Institute, School of Medicine, University of Minho, Braga | [jcsousa@med.uminho.pt](mailto:jcsousa@med.uminho.pt) |
| Maria Isabel Veiga | Life and Health Sciences Research Institute, School of Medicine, University of Minho, Braga | [mariaveiga@med.uminho.pt](mailto:mariaveiga@med.uminho.pt) |
| Diana Patrícia Pinto da Silva | Centro Médico da Praça | [diana_silva_28@hotmail.com](mailto:diana_silva_28@hotmail.com) |
| Ricardo Filipe Romão Ferreira | Centro Médico da Praça | [ricardo_ferreira1@hotmail.com](mailto:ricardo_ferreira1@hotmail.com) |
| Maria Beatriz Tomaz | Beatriz Godinho Saúde | [beatriztomaz@beatrizgodinho.pt](mailto:beatriztomaz@beatrizgodinho.pt) |
| Alfredo Rodrigues | Beatriz Godinho Saúde | [qualidade@beatrizgodinho.pt](mailto:qualidade@beatrizgodinho.pt) |
| Jácome Bruges Armas | Serviço Especializado de Epidemiologia e Biologia Molecular, Hospital de Santo Espírito da Ilha Terceira | brugesarmas@gmail.com |
| Paula Valente | Departamento de Saúde Pública e Planeamento, ARS Alentejo | Paula.Valente@arsalentejo.min-saude.pt |
| Cláudia Nunes dos Santos | Centro de Estudos de Doenças Crónicas, Faculdade de Ciências Médicas, Universidade Nova de Lisboa | claudia.nunes.santos@nms.unl.pt |
| Paulo Pereira | Centro de Estudos de Doenças Crónicas, Faculdade de Ciências Médicas, Universidade Nova de Lisboa | [paulo.pereira@nms.unl.pt](mailto:paulo.pereira@nms.unl.pt) |
| José Alves | Serviço de Patologia Clínica - Hospital Dr. Nélio Mendonça - SESARAM | [jose.alves@sesaram.pt](mailto:jose.alves@sesaram.pt) |
| Graça Andrade | Serviço de Patologia Clínica - Hospital Dr. Nélio Mendonça - SESARAM | [drgraca@sesaram.pt](mailto:drgraca@sesaram.pt) |
| Ludivina Freitas | Serviço de Patologia Clínica - Hospital Dr. Nélio Mendonça - SESARAM | [ludivina.freitas@sesaram.pt](mailto:ludivina.freitas@sesaram.pt) |
| Bruna R. Gouveia | Interactive Technologies Institute - LARSyS | bruna.gouveia@iasaude.madeira.gov.pt |
| Pedro Ramos | Secretaria Regional de Saúde e Proteção Civil - Governo Regional da Madeira | [pedro.ramos@madeira.gov.pt](mailto:pedro.ramos@madeira.gov.pt) |
| Herberto Jesus | Instituto de Administração da Saúde da Madeira | [herberto.jesus@iasaude.madeira.gov.pt](mailto:herberto.jesus@iasaude.madeira.gov.pt) |
| Maurício Melim | Instituto de Administração da Saúde da Madeira | [mauricio.melim@iasaude.madeira.gov.pt](mailto:mauricio.melim@iasaude.madeira.gov.pt) |
| Hugo Sousa | Serviço de Virologia, Instituto Português de Oncologia do Porto | [hugo.sousa@ipoporto.min-saude.pt](mailto:hugo.sousa@ipoporto.min-saude.pt) |
| Inês Baldaque | Serviço de Virologia, Instituto Português de Oncologia do Porto | [Ibaldaque@ipoporto.min-saude.pt](mailto:Ibaldaque@ipoporto.min-saude.pt) |
| Daniela Silva | ALS Controlvet , Tondela | Daniela.silva@ alsglobal.com |
| Inês Gomes | ALS Controlvet, Tondela | [ines.gomes@alsglobal.com](mailto:ines.gomes@alsglobal.com) |
| Eliana Costa | Serviço de Patologia Clínica, Centro Hospitalar de Trás-os-Montes e Alto Douro | [ecsvalente@chtmad.min-saude.pt](mailto:ecsvalente@chtmad.min-saude.pt) |
| Sara Sousa | Serviço de Patologia Clínica, Centro Hospitalar de Trás-os-Montes e Alto Douro | [sisousa@chtmad.min-saude.pt](mailto:sisousa@chtmad.min-saude.pt) |
| Ana Miguel Matos | Laboratório de Análises Clínicas da Universidade de Coimbra | [anamatos@ci.uc.pt](mailto:anamatos@ci.uc.pt) |
| Miguel Babarro Jorreto | Serviço de Imunohemoterapia, Unidade Local de Saúde do Alto Minho | [miguel.jorreto@ulsam.min-saude.pt](mailto:miguel.jorreto@ulsam.min-saude.pt) |
| Maria da Graça Maciel de Soveral | Serviço de Imunohemoterapia, Unidade Local de Saúde do Alto Minho | [maria.graca.barbosa@ulsam.min-saude.pt](mailto:maria.graca.barbosa@ulsam.min-saude.pt) |
| Luís Silva | Serviço de Patologia Clínica, Hospital de Vila Franca de Xira | [luis.silva@hvfx.pt](https://eur03.safelinks.protection.outlook.com/?url=http%3A%2F%2Fluis.silvahvfx.pt%2F&data=02%7C01%7CJ.Paulo.Gomes%40insa.min-saude.pt%7Cf74a1f7fa7b94e27789208d83bbf635f%7C22c84608f01d46c5802463cc962e5f51%7C1%7C0%7C637325040488764401&sdata=ccRaEblwntLxiIuPoh%2B7C9I1dXdtGsocpBYDEoC7FAU%3D&reserved=0) |
| Helena Ribeiro | Serviço de Patologia Clínica, Hospital de Vila Franca de Xira | [helena.ribeiro@hvfx.pt](mailto:helena.ribeiro@hvfx.pt) |
| Rita Rodrigues | Serviço de Patologia Clínica, Hospital de Vila Franca de Xira | [rita.silva.rodrigues@hvfx.pt](mailto:rita.silva.rodrigues@hvfx.pt) |
| Teresa Salvado | Serviço de Patologia Clínica, Hospital de Vila Franca de Xira | [teresa.salvado@hvfx.pt](mailto:teresa.salvado@hvfx.pt) |
| Luisa Mota-Vieira | Unidade de Genética e Patologia Moleculares, Hospital do Divino Espirito Santo de Ponta Delgada | [luisa.mq.vieira@azores.gov.pt](mailto:luisa.mq.vieira@azores.gov.pt) |
| Rita C. Veloso | Unidade de Genética e Patologia Moleculares, Hospital do Divino Espirito Santo de Ponta Delgada | [rita.tr.veloso@azores.gov.pt](mailto:rita.tr.veloso@azores.gov.pt) |
| Claudia C. Branco | Unidade de Genética e Patologia Moleculares, Hospital do Divino Espirito Santo de Ponta Delgada | [claudia.ma.branco@azores.gov.pt](mailto:claudia.ma.branco@azores.gov.pt) |
| Sónia Marta Santos Magalhães | Hospital Agostinho Ribeiro-Felgueiras | [analises.har@gmail.com](mailto:analises.har@gmail.com) |
| Helena Rodrigues | Laboratório Dra Helena Rodrigues, Valença | administracao@helenarodriguesanalisesclinicas.pt |
| Francisca Rocha | Laboratório Dra Helena Rodrigues, Valença | franciscarocha@helenarodriguesanalisesclinicas.pt |
| Sandra Paulo | Serviço de Patologia Clínica da Unidade Local de Saúde de Castelo Branco | [sandra.paulo@ulscb.min-saude.pt](mailto:sandra.paulo@ulscb.min-saude.pt) |
| Mariana Martins | Serviço de Patologia Clínica da Unidade Local de Saúde de Castelo Branco | [mcmartins@ulscb.min-saude.pt](mailto:mcmartins@ulscb.min-saude.pt) |
| Mariana Viana | Centro Hospitalar Tâmega e Sousa, Penafiel | [patclinica@chts.min-saude.pt](mailto:patclinica@chts.min-saude.pt ) |
| Maria Calle Vellés | Centro Hospitalar Tâmega e Sousa, Penafiel | callemaria@chts.min-saude.pt |
| Miguel Pinheiro | iBiMED/Universidade de Aveiro | monsantopinheiro@gmail.com |
| Miguel Fevereiro | Instituto Nacional de Investigação Agrária e Veterinária | miguel.fevereiro@iniav.pt |
| Ana Margarida Henriques | Instituto Nacional de Investigação Agrária e Veterinária | [margarida.henriques@iniav.pt](mailto:margarida.henriques@iniav.pt) |
| Tiago Luís | Instituto Nacional de Investigação Agrária e Veterinária | [tiago.luis@iniav.pt](mailto:tiago.luis@iniav.pt) |
| Cathy Paulino | Instituto Gulbenkian de Ciência, Oeiras | [cfpaulino@igc.gulbenkian.pt](mailto:cfpaulino@igc.gulbenkian.pt) |
| João Costa | Instituto Gulbenkian de Ciência, Oeiras | [jcosta@igc.gulbenkian.pt](mailto:jcosta@igc.gulbenkian.pt) |
| João Sobral | Instituto Gulbenkian de Ciência, Oeiras | [jsobral@igc.gulbenkian.pt](mailto:jsobral@igc.gulbenkian.pt) |
| Susana Ladeiro | Instituto Gulbenkian de Ciência, Oeiras | [sladeiro@igc.gulbenkian.pt](mailto:sladeiro@igc.gulbenkian.pt) |
| Jorge Machado | Instituto Nacional de Saúde Dr Ricardo Jorge (INSA), Lisboa | [jorge.machado@insa.min-saude.pt](mailto:jorge.machado@insa.min-saude.pt) |
| Hugo Martiniano | Instituto Nacional de Saúde Dr Ricardo Jorge (INSA), Lisboa | [hugo.martiniano@insa.mi-saude.pt](mailto:hugo.martiniano@insa.mi-saude.pt) |
| Paula Bajanca-Lavado | Instituto Nacional de Saúde Dr Ricardo Jorge (INSA), Lisboa | [paula.lavado@insa.min-saude.pt](mailto:paula.lavado@insa.min-saude.pt) |
| Maria José Borrego | Instituto Nacional de Saúde Dr Ricardo Jorge (INSA), Lisboa | [m.jose.borrego@insa.min-saude.pt](mailto:m.jose.borrego@insa.min-saude.pt) |
| Líbia Zé-Zé | Instituto Nacional de Saúde Dr Ricardo Jorge (INSA), Lisboa | [libia.zeze@insa.min-saude.pt](mailto:libia.zeze@insa.min-saude.pt) |
| Nuno Verdasca | Instituto Nacional de Saúde Dr Ricardo Jorge (INSA), Lisboa | [nuno.verdasca@insa.min-saude.pt](mailto:nuno.verdasca@insa.min-saude.pt) |
| Sílvia Lopo | Instituto Nacional de Saúde Dr Ricardo Jorge (INSA), Lisboa | [silvia.lopo@insa.min-saude.pt](mailto:silvia.lopo@insa.min-saude.pt) |
| Rita de Sousa | Instituto Nacional de Saúde Dr Ricardo Jorge (INSA), Lisboa | [rita.sousa@insa.min-saude.pt](mailto:rita.sousa@insa.min-saude.pt) |
| Maria João Gargate | Instituto Nacional de Saúde Dr Ricardo Jorge (INSA), Lisboa | [m.joao.gargate@insa.min-saude.pt](mailto:m.joao.gargate@insa.min-saude.pt) |
| Susana Martins | Instituto Nacional de Saúde Dr Ricardo Jorge (INSA), Lisboa | [susana.martins@insa.min-saude.pt](mailto:susana.martins@insa.min-saude.pt) |
| Isabel Lopes de Carvalho | Instituto Nacional de Saúde Dr Ricardo Jorge (INSA), Lisboa | [isabel.carvalho@insa.min-saude.pt](mailto:isabel.carvalho@insa.min-saude.pt) |
| Célia Rodrigues Bettencourt | Instituto Nacional de Saúde Dr Ricardo Jorge (INSA), Lisboa | [celia.betencourt@insa.min-saude.pt](mailto:celia.betencourt@insa.min-saude.pt) |
| Carla Roque | Instituto Nacional de Saúde Dr Ricardo Jorge (INSA), Lisboa | [carla.roque@insa.min-saude.pt](mailto:carla.roque@insa.min-saude.pt) |
| Leonor Silveira | Instituto Nacional de Saúde Dr Ricardo Jorge (INSA), Lisboa | [leonor.silveira@insa.min-saude.pt](mailto:leonor.silveira@insa.min-saude.pt) |
| João Rodrigues | Instituto Nacional de Saúde Dr Ricardo Jorge (INSA), Lisboa | [joao.rodrigues@insa.min-saude.pt](mailto:joao.rodrigues@insa.min-saude.pt) |
| Ivone Água-Doce | Instituto Nacional de Saúde Dr Ricardo Jorge (INSA), Lisboa | [ivone.agua-doce@insa.min-saude.pt](mailto:ivone.agua-doce@insa.min-saude.pt) |
| Rita Cordeiro | Instituto Nacional de Saúde Dr Ricardo Jorge (INSA), Lisboa | [rita.cordeiro@insa.min-saude.pt](mailto:rita.cordeiro@insa.min-saude.pt) |
| Ana Pelerito | Instituto Nacional de Saúde Dr Ricardo Jorge (INSA), Lisboa | [ana.pelerito@insa.min-saude.pt](mailto:ana.pelerito@insa.min-saude.pt) |
| Cristina Correia | Instituto Nacional de Saúde Dr Ricardo Jorge (INSA), Lisboa | [cristina.correia@insa.min-saude.pt](mailto:cristina.correia@insa.min-saude.pt) |
| Vera Manageiro | Instituto Nacional de Saúde Dr Ricardo Jorge (INSA), Lisboa | [vera.manageiro@insa.min-saude.pt](mailto:vera.manageiro@insa.min-saude.pt) |
| Raquel Rocha | Instituto Nacional de Saúde Dr Ricardo Jorge (INSA), Lisboa | [maria.rocha@insa.min-saude.pt](mailto:maria.rocha@insa.min-saude.pt) |
| Raquel Neves | Instituto Nacional de Saúde Dr Ricardo Jorge (INSA), Lisboa | [raquel.neves@insa.min-saude.pt](mailto:raquel.neves@insa.min-saude.pt) |
| Paula Palminha | Instituto Nacional de Saúde Dr Ricardo Jorge (INSA), Lisboa | [paula.palminha@insa.min-saude.pt](mailto:paula.palminha@insa.min-saude.pt) |
| Cristina Veríssimo | Instituto Nacional de Saúde Dr Ricardo Jorge (INSA), Lisboa | [cristina.verissimo@insa.min-saude.pt](mailto:cristina.verissimo@insa.min-saude.pt) |
| Elizabeth Pádua | Instituto Nacional de Saúde Dr Ricardo Jorge (INSA), Lisboa | [elizabeth.padua@insa.min-saude.pt](mailto:elizabeth.padua@insa.min-saude.pt) |
| Rita Matos | Instituto Nacional de Saúde Dr Ricardo Jorge (INSA), Lisboa | [rita.matos@insa.min-saude.pt](mailto:rita.matos@insa.min-saude.pt) |
| Susana Silva | Instituto Nacional de Saúde Dr Ricardo Jorge (INSA), Lisboa | susana.pereira@insa.min-saude.pt |
| Alexandra Nunes | Instituto Nacional de Saúde Dr Ricardo Jorge (INSA), Lisboa | [alexandra.nunes@insa.min-saude.pt](mailto:alexandra.nunes@insa.min-saude.pt) |
| Pedro Pechirra | Instituto Nacional de Saúde Dr Ricardo Jorge (INSA), Lisboa | [pedro.pechirra@insa.min-saude.pt](mailto:pedro.pechirra@insa.min-saude.pt) |
| Inês Costa | Instituto Nacional de Saúde Dr Ricardo Jorge (INSA), Lisboa | [ines.costa@insa.min-saude.pt](mailto:ines.costa@insa.min-saude.pt) |
| Mónica Oleastro | Instituto Nacional de Saúde Dr Ricardo Jorge (INSA), Lisboa | [monica.oleastro@insa.min-saude.pt](mailto:monica.oleastro@insa.min-saude.pt) |
| Carla Feliciano | Instituto Nacional de Saúde Dr Ricardo Jorge (INSA), Lisboa | carla.feliciano@insa.min-saude.pt |
| Isabel Albergaria | Instituto Nacional de Saúde Dr Ricardo Jorge (INSA), Lisboa | m.isabel.albergaria@insa.min-saude.pt |
| Fernanda Vilarinho | Instituto Nacional de Saúde Dr Ricardo Jorge (INSA), Lisboa | fernanda.vilarinho@insa.min-saude.pt |
| Márcia Faria | Instituto Nacional de Saúde Dr Ricardo Jorge (INSA), Lisboa | marcia.faria@insa.min-saude.pt |
| Margarida Vaz | Instituto Nacional de Saúde Dr Ricardo Jorge (INSA), Lisboa | margarida.vaz@insa.min-saude.pt |
| Patrícia Barros | Instituto Nacional de Saúde Dr Ricardo Jorge (INSA), Lisboa | patricia.barros@insa.min-saude.pt |
| Raquel Rodrigues | Instituto Nacional de Saúde Dr Ricardo Jorge (INSA), Lisboa | raquel.rodrigues@insa.min-saude.pt |
| José Vicente Constantino | Instituto Nacional de Saúde Dr Ricardo Jorge (INSA), Lisboa | jose.constantino@insa.min-saude.pt |
| Rita Macedo | Instituto Nacional de Saúde Dr Ricardo Jorge (INSA), Lisboa | [rita.macedo@insa.min-saude.pt](mailto:rita.macedo@insa.min-saude.pt) |
| Raquel Sabino | Instituto Nacional de Saúde Dr Ricardo Jorge (INSA), Lisboa | raquel.sabino@insa.min-saude.pt |
| Idalina Ferreira | Instituto Nacional de Saúde Dr Ricardo Jorge (INSA), Lisboa | [idalina.ferreira@insa.min-saude.pt](mailto:idalina.ferreira@insa.min-saude.pt) |
| Sónia Silva | Instituto Nacional de Saúde Dr Ricardo Jorge (INSA), Lisboa | sonia.silva@insa.min-saude.pt |
| Anabela Vilares | Instituto Nacional de Saúde Dr Ricardo Jorge (INSA), Lisboa | [anabela.vilares@insa.min-saude.pt](mailto:anabela.vilares@insa.min-saude.pt) |
