# Supplementary material for: Massive dissemination of a SARS-CoV-2 Spike Y839 variant in Portugal
Source: Emerg Microbes Infect. 2020 Nov 25;9(1):2488–96. doi: 10.1080/22221751.2020.1844552 (PMC7717510; doi:10.1080/22221751.2020.1844552)
Supplement: Supplementary_Information.docx [file TEMI_A_1844552_SM8935.docx]

**Supplementary Information**

**Supplementary methods**

**SARS-CoV-2 amplicon-based genome amplification and sequencing**

SARS-CoV-2 positive RNA samples were subjected to genome sequencing using a whole-genome amplification strategy with tiled, multiplexed primers^1^ and the Artic Consortium protocol (<https://artic.network/ncov-2019>; <https://www.protocols.io/view/ncov-2019-sequencing-protocol-bbmuik6w>), with slight modifications. In brief, after cDNA synthesis using SuperScript™ IV First-Strand Synthesis System kit (Invitrogen™, catalog: 18091050) with random hexamers from 11 μL of RNA (exactly as described in Artic Network protocol), targeted amplification was performed with 2·5 μL of cDNA using NEBNExt® Q5® HotStart HiFi Master Mix (12·5 μL per reaction) (New England BioLabs, catalog: M0544S) with two pools of tiling primers (A and B) separately. Primers versions V1 and V2 (aliquots kindly provided by Artic Network team) were used for the first 243 samples of this study, while the V3 primers (with a total of 218 primers) were applied to all samples afterwards (all versions available here: (<https://github.com/artic-network/artic-ncov2019/tree/master/primer_schemes/nCoV-2019>). The final concentration per primer (V3 version) was ~0·013µM (1·4 µM per pool) in a 25 μL total reaction volume. PCR amplification parameters were: 30s at 98^o^C, 35 cycles of 15s at 98^o^C and 5 min at 65^o^C (for the first 762 samples) or 63^o^C (afterwards), and final extension for 5 min at 65/63^o^C. Amplicons were visualized on a 1% agarose gel, tubes A and B were pooled per sample, and subjected to clean up with Agencourt AMPure XP (Beckman Coulter, catalog: A63880) using a 1:1 volume ratio. Purified amplicons were quantified using Qubit fluorometer (Thermo Fisher Scientific) and normalized to a concentration of 0·4 ng/ul. Dual-indexed sequencing libraries were prepared using Illumina Nextera XT DNA Library Prep Kit (Illumina). Pooling, denaturation and dilution of bead-based normalized libraries was performed according to the manufacturer´s instructions for the MiSeq or NextSeq 550 systems (Illumina). A 1% spike-in PhiX genome library (Illumina) was used as internal quality control. Libraries were sequenced using 250bp (MiSeq) or 150bp (NextSeq 550) paired-end reads targeting ~1M reads per sample.

**Genome assembly and sequence curation**

Analysis of sequence read data was conducted using the bioinformatics pipeline implemented in INSaFLU (<https://insaflu.insa.pt/>; <https://github.com/INSaFLU>), which is a web-based (and also locally installable) platform for amplicon-based next-generation sequencing data analysis^2^. Briefly, the core bioinformatics steps (documented in Borges et al, 2018 and <https://insaflu.readthedocs.io/>) involved: i) raw NGS reads quality analysis and improvement using FastQC; (<https://www.bioinformatics.babraham.ac.uk/projects/fastqc>) and Trimmomatic (<http://www.usadellab.org/cms/index.php?page=trimmomatic>), respectively (read’s ends were cropped 30bp for primer clipping); ii) draft *de novo* assembly using SPAdes (<http://cab.spbu.ru/software/spades/>) followed by classification and contigs assignment of Human Betacoronavirus; and, iii) reference-based mapping, consensus generation and variant detection using the multisoftware tool Snippy (<https://github.com/tseemann/snippy>), using the Wuhan-Hu-1/2019 genome sequence (<https://www.ncbi.nlm.nih.gov/nuccore/MN908947>) as reference. The first 40bp and end 100bp were discarded and consensus sequences were exclusively included in the study when >70% of genome was covered by at least 10-fold. For samples with coverage drop below 10-fold, fine-tuned consensus sequence curation was performed as follows: i) undefined bases (“N”) were placed in genome regions with depth of coverage below 10 using a python script (<https://github.com/rfm-targa/BioinfUtils/blob/master/msa_masker.py>), and “N” regions at the sequence ends were trimmed to avoid releasing sequences starting or ending with “N”; ii) all regions with depth coverage below 10 were visually inspected in the Integrative Genomics Viewer (<http://www.broadinstitute.org/igv>) available *in situ* at INSaFLU, and when mutations were detected and validated within these low coverage regions, they were inserted in the consensus sequence to avoid disrupting/biasing the phylogenetic signal; iii) when a mutation was validated within a “N” region with <=100bp, the whole region was inserted (as long as the region was covered by at least one read); when a mutation was validated within a “N” region with >100bp, the mutation was inserted together with additional 20 bp (10bp from each flanking side) to improve the downstream sequence alignment.

**SARS-CoV-2 Phylogenetic analysis using Nextstrain**

A total of 1516 SARS-CoV-2 genome sequences were analyzed in this study (corresponding to INSA’s collection as of July 23^rd^, 2020; Table S1) using the SARS-CoV-2 Nextstrain pipeline^3^ version from March 23, 2020 (<https://github.com/nextstrain/ncov>), with slight modifications. In brief, sequences were aligned against the reference Wuhan-Hu-1/2019 genome of SARS-CoV-2 (GenBank accession MN908947) using MAFFT^4^, the alignment was visually inspected, manually curated and further used to build a maximum likelihood phylogenetic tree based on the GTR model using IQ-TREE^5^ following the Nextstrain implementation (the first 130bp and last 50 bp, as well as a few bases within the alignment, were masked as likely sequencing artifacts). Within the Nextstrain pipeline, Treetime^6^ is applied to infer a time-resolved phylogeny. The phylogeny is rooted relative to early samples from Wuhan, China (Wuhan-Hu-1/2019, GenBank accession MN908947; Wuhan/WH01/2019, GenBank accession LR757998) and temporal resolution assumes a nucleotide substitution rate of 0.0008±0.0004 substitutions *per* site *per* year.

**Real-time data sharing of SARS-CoV-2 genetic diversity and geotemporal spread in Portugal**

A website (<https://insaflu.insa.pt/covid19>) was launched on March 28, 2020 for real-time data sharing on SARS-CoV-2 genetic diversity and geotemporal spread in Portugal. This site gives access to “situation reports of the study and provides interactive data navigation using both Nextstrain (<https://nextstrain.org/>)^3^ and Microreact (<https://microreact.org/>)^7^ tools. As of July 23^rd^, 2020, “clade" assignment reflects the Nextstrain classification (<https://github.com/nextstrain/ncov>; version June 3, 2020), while "Lineage" refers to the classification based on Phylogenetic Assignment of Named Global Outbreak Lineages (Pangolin) (<https://github.com/hCoV-2019/pangolin> - lineage_version: 2020-05-07)^8^.

In this study, both Nextstrain (<https://nextstrain.org/>)^3^ and Microreact (<https://microreact.org/>)^7^ visualization tools were used to deeply explore the genetic diversity and geotemporal spread dynamics of SARS-CoV-2 Spike D839Y variant in Portugal. The phylogeny and associated metadata used in this study can be visualized interactively at <https://microreact.org/project/nDGsJKFv7gQTj1q8CQwwKR/18a0a470> (global dataset, as of July 23^rd^, 2020), <https://microreact.org/project/nDGsJKFv7gQTj1q8CQwwKR/0489f840> (geographic resolution by Region, genomes collected until April 30^th^ highlighted) and <https://microreact.org/project/2kh3TRVYB9gWGRpNSJWDW5/b6c659e0> (geographic resolution by District, genomes collected until April 30^th^ highlighted)) using Microreact (<https://microreact.org/>)^7^. To explore the frequency of SARS-CoV-2 Spike D839Y variant at worldwide level, we downloaded 66548 amino acid sequences (and associated metadata) of SARS-CoV-2 spike protein available at GISAID (as of 23 July 2020) and aligned them using MAFFT^4^. A total of 65367 sequences collected outside Portugal had sequence data for amino acid position 839, giving a total of 66883 (65367 plus 1516) sequences screened in this study for the presence of mutations in this amino acid of interest. The 92 genomes sequences of the SARS-CoV-2 Spike D839Y variant detected abroad were downloaded from GISAD (GISAID acknowledgments are in Table S2). Their clade classification and integration into “global” and Portugal phylogeny was performed using Nextstrain (<https://nextstrain.org/ncov>) and Nextclade (<https://clades.nextstrain.org/>).

**References**

1. Quick J, Grubaugh ND, Pullan ST, et al. Multiplex PCR method for MinION and Illumina sequencing of Zika and other virus genomes directly from clinical samples. *Nat Protoc* 2017; **12**(6):1261–1276. doi:10.1038/nprot.2017.066
2. Borges V, Pinheiro M, Pechirra P, Guiomar R, Gomes JP. INSaFLU: an automated open web-based bioinformatics suite "from-reads" for influenza whole-genome-sequencing-based surveillance. *Genome Med* 2018; **10**(1):46. Published 2018 Jun 29. doi:10.1186/s13073-018-0555-0
3. Hadfield J, Megill C, Bell SM, et al. Nextstrain: real-time tracking of pathogen evolution. *Bioinformatics* 2018; **34**(23):4121–4123. doi:10.1093/bioinformatics/bty407
4. Katoh K, Misawa K, Kuma K, Miyata T. MAFFT: a novel method for rapid multiple sequence alignment based on fast Fourier transform. *Nucleic Acids Res* 2002; **30**(14):3059–3066. doi:10.1093/nar/gkf436
5. Nguyen LT, Schmidt HA, von Haeseler A, Minh BQ. IQ-TREE: a fast and effective stochastic algorithm for estimating maximum-likelihood phylogenies. *Mol Biol Evol* 2015; **32**(1):268–274. doi:10.1093/molbev/msu300
6. Sagulenko P, Puller V, Neher RA. TreeTime: Maximum-likelihood phylodynamic analysis. *Virus Evol* 2018; **4**(1):vex042. doi:10.1093/ve/vex042
7. Argimón S, Abudahab K, Goater RJE, et al. Microreact: visualizing and sharing data for genomic epidemiology and phylogeography. *Microb Genom* 2016; **2**(11):e000093. doi:10.1099/mgen.0.000093
8. Rambaut A, Holmes EC, O'Toole Á, et al. A dynamic nomenclature proposal for SARS-CoV-2 lineages to assist genomic epidemiology [published online ahead of print, 2020 Jul 15]. Nat Microbiol 2020;10.1038/s41564-020-0770-5. doi:10.1038/s41564-020-0770-5

**Supplementary Figures and Tables**

**
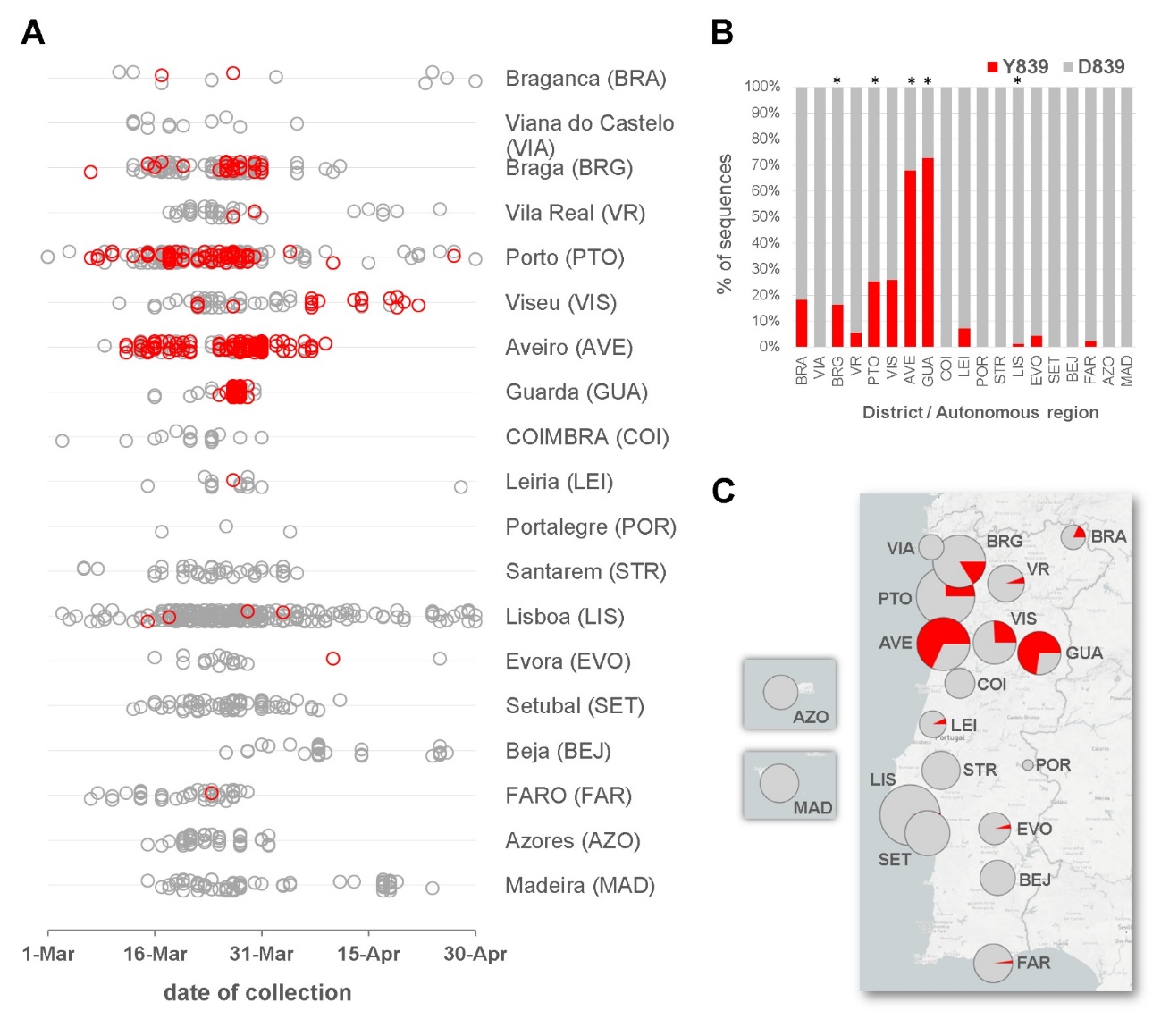
**

**Figure S1. Landscape of the geotemporal spread of SARS-CoV-2 Spike Y839 variant in Portugal by District, as of April 30^th,^ 2020. A.** Distribution of the analysed genome sequences (n=1500) by date of sample collection and District, highligthing COVID-19 cases caused by the Spike Y839 variant (red dots). **B.** Relative frequency of the Spike Y839 variant across the 11 Districts where the variant was detected until April 30^th^. Asterisks above the graph denote Districts where more than 50 genomes were sampled. **C.** Relative frequency of Spike Y839 variant by District until the end of April 2020. The phylogeny and geotemporal distribution can be visualized interactively at <https://microreact.org/project/nDGsJKFv7gQTj1q8CQwwKR/0489f840> (geographic resolution by Region) and <https://microreact.org/project/2kh3TRVYB9gWGRpNSJWDW5/b6c659e0> (geographic resolution by District) using Microreact (<https://microreact.org/>).

**
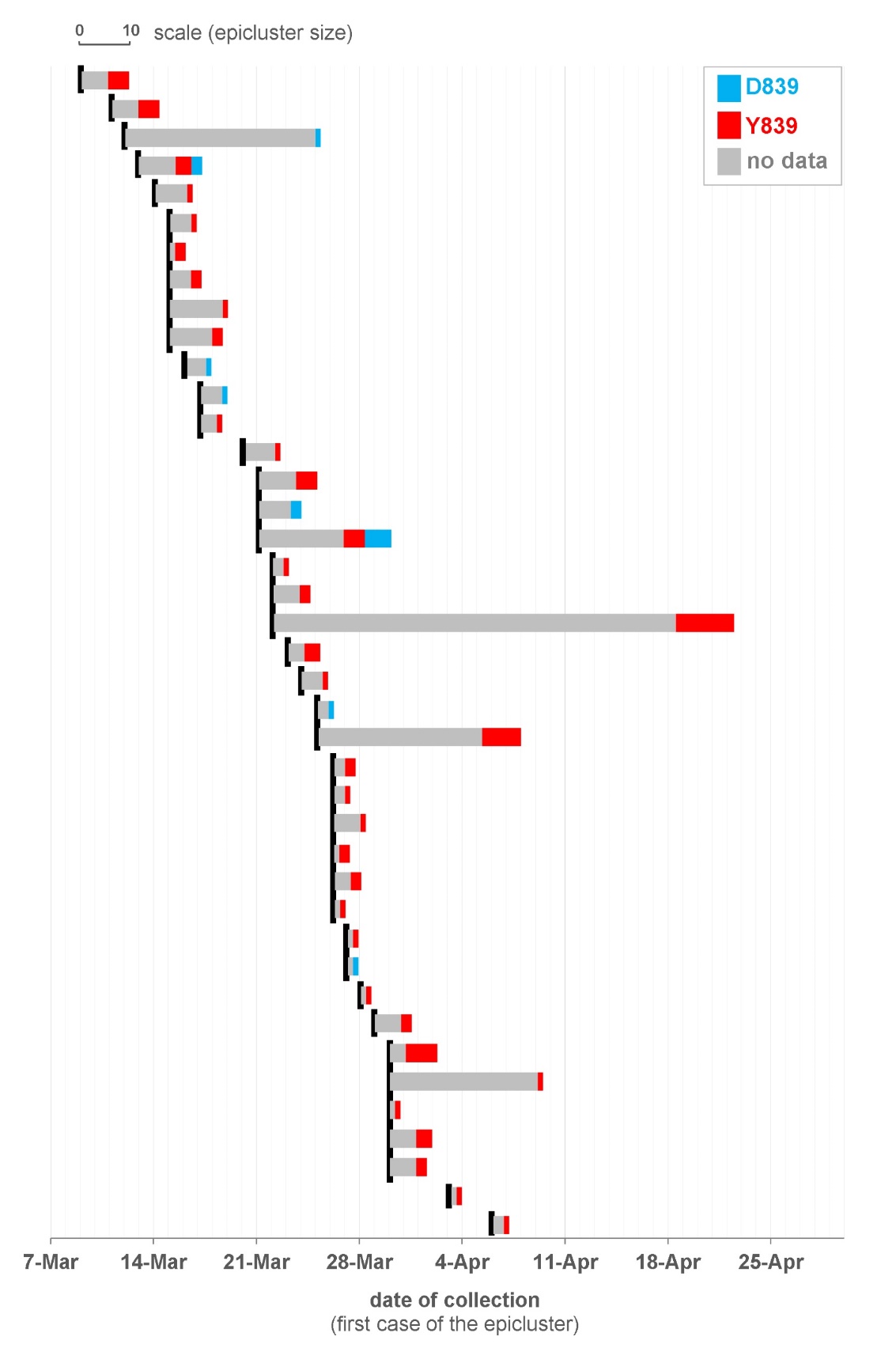
**

**Figure S2. SARS-CoV-2 D839/Y839 status of** **epiclusters of potential epidemiologically linked confirmed cases (“epiclusters”) monitored by the Public Health Unit of Primary Care Cluster of Baixo Vouga, as of April 30^th^, 2020.** The graph shows the temporal distribution (by date of sample collection of the first case of the epicluster; black lines) of 41 potential “epiclusters” (covering a total of 420 confirmed cases) for which SARS-CoV-2 genome data is already available. 33 epiclusters (323 confirmed cases) (77%) are exclusively associated with the Spike Y839 variant. The two potential epiclusters where both D839 and Y839 variants were ambiguously detected are under close contact tracing investigation to disclose this incongruence.

**
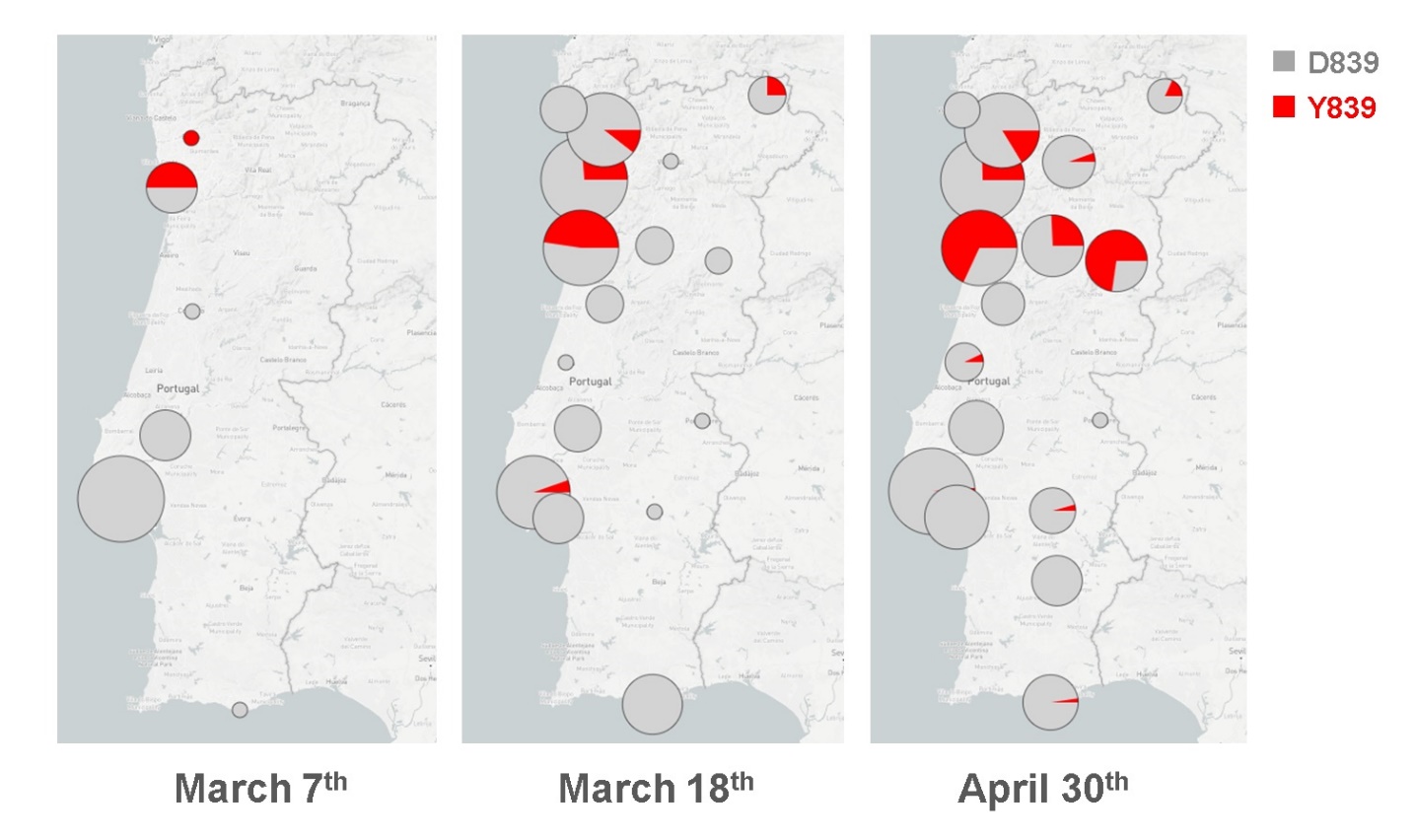
**

**Figure S3. Geographical distribution of the relative frequencies of the D839 (gray) and Y839 (red) variants by District in three timeframes: March 7^th^** (when the first Y839 genomes were detected)**, March 18^th^** (when the emergency state was declared in Portugal and national lockdown was implemented) **and April 30^th,^ 2020.** Autonomous regions (Azores and Madeira) are not shown in these maps as no Y839 case where detected there (see Figure S1).The phylogeny and geotemporal distribution can be visualized interactively at <https://microreact.org/project/nDGsJKFv7gQTj1q8CQwwKR/0489f840> (geographic resolution by Region) and <https://microreact.org/project/2kh3TRVYB9gWGRpNSJWDW5/b6c659e0> (geographic resolution by District) using Microreact (<https://microreact.org/>).


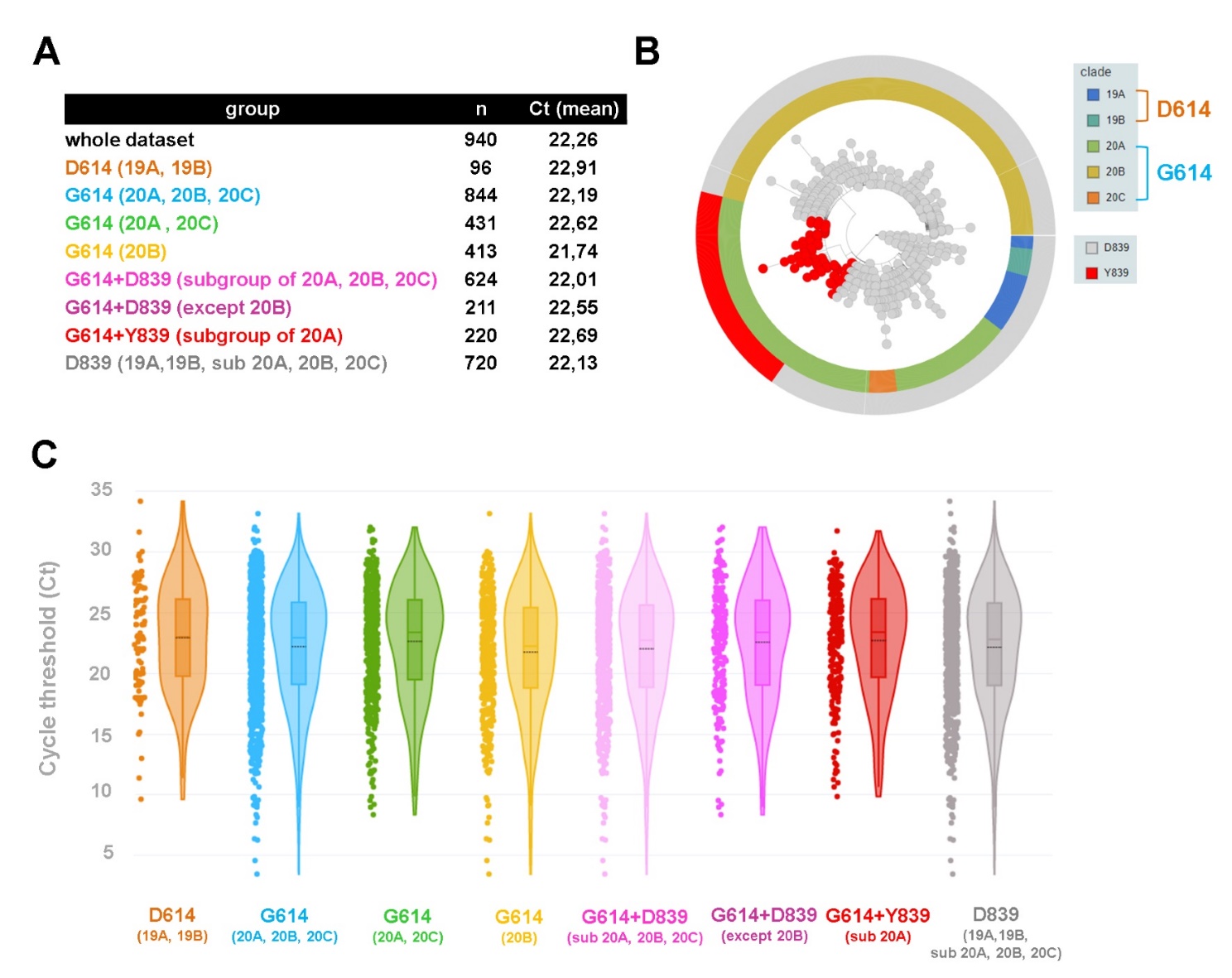


**Figure S4. Comparison of Cycle threshold (Ct) values obtained in diagnostic PCR between different samples groups.** Groups were established according to phylogenetic clustering and Nextstrain Clade classification of the genomes studied, D614/G614 status, phylogenetic group within G614 (i.e., 20B or non-20B) and Spike D839/Y839 status. **A.** Mean Ct values observed per group. **B.** Global phylogeny of the 1516 genomes studied (<https://microreact.org/project/nDGsJKFv7gQTj1q8CQwwKR/f46f1fa4>) highlighting the Nextstrain clades**,** D614/G614 and D839/Y839 status. **C.** Violin, scatter and box plots showing the dispersion of Ct values per group. Mean Ct values are indicated by black dash lines.

| **Table S1. List of SARS-CoV-2 genome sequences from Portugal used in this study.** | | | | |
| --- | --- | --- | --- | --- |
|  |  |  |  |  |
| **Sample designation** | **Location** | **Spike Y839 variant** | **Collection date** | **GISAID Accession ID** |
| Portugal/CV62/2020 | Europe / Portugal |  | 2020-03-01 | EPI_ISL_413647 |
| Portugal/CV63/2020 | Europe / Portugal |  | 2020-03-01 | EPI_ISL_413648 |
| Portugal/PT0003/2020 | Europe / Portugal |  | 2020-03-03 | EPI_ISL_417987 |
| Portugal/PT0025/2020 | Europe / Portugal |  | 2020-03-03 | EPI_ISL_418010 |
| Portugal/PT0005/2020 | Europe / Portugal |  | 2020-03-04 | EPI_ISL_417989 |
| Portugal/PT0026/2020 | Europe / Portugal |  | 2020-03-04 | EPI_ISL_418011 |
| Portugal/PT0004/2020 | Europe / Portugal |  | 2020-03-05 | EPI_ISL_417988 |
| Portugal/PT0006a/2020 | Europe / Portugal |  | 2020-03-06 | EPI_ISL_417990 |
| Portugal/PT0027/2020 | Europe / Portugal |  | 2020-03-06 | EPI_ISL_418012 |
| Portugal/PT0007/2020 | Europe / Portugal |  | 2020-03-07 | EPI_ISL_417992 |
| Portugal/PT0012/2020 | Europe / Portugal | yes | 2020-03-07 | EPI_ISL_417997 |
| Portugal/PT0014/2020 | Europe / Portugal | yes | 2020-03-07 | EPI_ISL_417999 |
| Portugal/PT0028/2020 | Europe / Portugal |  | 2020-03-07 | EPI_ISL_418013 |
| Portugal/PT0008/2020 | Europe / Portugal |  | 2020-03-08 | EPI_ISL_417993 |
| Portugal/PT0009/2020 | Europe / Portugal |  | 2020-03-08 | EPI_ISL_417994 |
| Portugal/PT0010/2020 | Europe / Portugal |  | 2020-03-08 | EPI_ISL_417995 |
| Portugal/PT0011/2020 | Europe / Portugal |  | 2020-03-08 | EPI_ISL_417996 |
| Portugal/PT0013/2020 | Europe / Portugal | yes | 2020-03-08 | EPI_ISL_417998 |
| Portugal/PT1516/2020 | Europe / Portugal | yes | 2020-03-08 | EPI_ISL_511475 |
| Portugal/PT0029/2020 | Europe / Portugal |  | 2020-03-09 | EPI_ISL_418014 |
| Portugal/PT0030/2020 | Europe / Portugal |  | 2020-03-09 | EPI_ISL_418015 |
| Portugal/PT0285/2020 | Europe / Portugal |  | 2020-03-09 | EPI_ISL_454000 |
| Portugal/PT0286a/2020 | Europe / Portugal |  | 2020-03-09 | EPI_ISL_454001 |
| Portugal/PT0287/2020 | Europe / Portugal |  | 2020-03-09 | EPI_ISL_454003 |
| Portugal/PT1517/2020 | Europe / Portugal |  | 2020-03-09 | EPI_ISL_511476 |
| Portugal/PT0015/2020 | Europe / Portugal |  | 2020-03-10 | EPI_ISL_418000 |
| Portugal/PT0016/2020 | Europe / Portugal |  | 2020-03-10 | EPI_ISL_418001 |
| Portugal/PT0018/2020 | Europe / Portugal | yes | 2020-03-10 | EPI_ISL_418003 |
| Portugal/PT0031/2020 | Europe / Portugal |  | 2020-03-10 | EPI_ISL_418016 |
| Portugal/PT0288/2020 | Europe / Portugal | yes | 2020-03-10 | EPI_ISL_454004 |
| Portugal/PT0289/2020 | Europe / Portugal |  | 2020-03-10 | EPI_ISL_454005 |
| Portugal/PT0017/2020 | Europe / Portugal |  | 2020-03-11 | EPI_ISL_418002 |
| Portugal/PT0291/2020 | Europe / Portugal |  | 2020-03-11 | EPI_ISL_454007 |
| Portugal/PT0498/2020 | Europe / Portugal |  | 2020-03-11 | EPI_ISL_454222 |
| Portugal/PT0019/2020 | Europe / Portugal |  | 2020-03-12 | EPI_ISL_418004 |
| Portugal/PT0020/2020 | Europe / Portugal |  | 2020-03-12 | EPI_ISL_418005 |
| Portugal/PT0290/2020 | Europe / Portugal | yes | 2020-03-12 | EPI_ISL_454006 |
| Portugal/PT0292/2020 | Europe / Portugal |  | 2020-03-12 | EPI_ISL_454008 |
| Portugal/PT0293/2020 | Europe / Portugal | yes | 2020-03-12 | EPI_ISL_454009 |
| Portugal/PT0294/2020 | Europe / Portugal | yes | 2020-03-12 | EPI_ISL_454010 |
| Portugal/PT0021/2020 | Europe / Portugal |  | 2020-03-13 | EPI_ISL_418006 |
| Portugal/PT0022/2020 | Europe / Portugal |  | 2020-03-13 | EPI_ISL_418007 |
| Portugal/PT0023/2020 | Europe / Portugal |  | 2020-03-13 | EPI_ISL_418008 |
| Portugal/PT0295/2020 | Europe / Portugal |  | 2020-03-13 | EPI_ISL_454011 |
| Portugal/PT0296/2020 | Europe / Portugal |  | 2020-03-13 | EPI_ISL_454012 |
| Portugal/PT0297/2020 | Europe / Portugal |  | 2020-03-13 | EPI_ISL_454013 |
| Portugal/PT0298/2020 | Europe / Portugal | yes | 2020-03-13 | EPI_ISL_454014 |
| Portugal/PT0299/2020 | Europe / Portugal |  | 2020-03-13 | EPI_ISL_454015 |
| Portugal/PT0300/2020 | Europe / Portugal |  | 2020-03-13 | EPI_ISL_454016 |
| Portugal/PT0499/2020 | Europe / Portugal |  | 2020-03-13 | EPI_ISL_454223 |
| Portugal/PT0606/2020 | Europe / Portugal |  | 2020-03-13 | EPI_ISL_454329 |
| Portugal/PT0607/2020 | Europe / Portugal |  | 2020-03-13 | EPI_ISL_454330 |
| Portugal/PT1509a/2020 | Europe / Portugal |  | 2020-03-13 | EPI_ISL_511468 |
| Portugal/PT0301/2020 | Europe / Portugal | yes | 2020-03-14 | EPI_ISL_454017 |
| Portugal/PT0303/2020 | Europe / Portugal | yes | 2020-03-14 | EPI_ISL_454019 |
| Portugal/PT0304/2020 | Europe / Portugal |  | 2020-03-14 | EPI_ISL_454020 |
| Portugal/PT0305/2020 | Europe / Portugal |  | 2020-03-14 | EPI_ISL_454021 |
| Portugal/PT0306/2020 | Europe / Portugal |  | 2020-03-14 | EPI_ISL_454022 |
| Portugal/PT0307/2020 | Europe / Portugal |  | 2020-03-14 | EPI_ISL_454023 |
| Portugal/PT0309/2020 | Europe / Portugal |  | 2020-03-14 | EPI_ISL_454025 |
| Portugal/PT0310/2020 | Europe / Portugal | yes | 2020-03-14 | EPI_ISL_454026 |
| Portugal/PT0311/2020 | Europe / Portugal |  | 2020-03-14 | EPI_ISL_454027 |
| Portugal/PT0312/2020 | Europe / Portugal |  | 2020-03-14 | EPI_ISL_454028 |
| Portugal/PT0315/2020 | Europe / Portugal |  | 2020-03-14 | EPI_ISL_454031 |
| Portugal/PT0316/2020 | Europe / Portugal |  | 2020-03-14 | EPI_ISL_454032 |
| Portugal/PT0342/2020 | Europe / Portugal |  | 2020-03-14 | EPI_ISL_454058 |
| Portugal/PT0500/2020 | Europe / Portugal |  | 2020-03-14 | EPI_ISL_454224 |
| Portugal/PT0605/2020 | Europe / Portugal |  | 2020-03-14 | EPI_ISL_454328 |
| Portugal/PT0608/2020 | Europe / Portugal |  | 2020-03-14 | EPI_ISL_454331 |
| Portugal/PT0609/2020 | Europe / Portugal |  | 2020-03-14 | EPI_ISL_454332 |
| Portugal/PT0610/2020 | Europe / Portugal |  | 2020-03-14 | EPI_ISL_454333 |
| Portugal/PT0694/2020 | Europe / Portugal |  | 2020-03-14 | EPI_ISL_510953 |
| Portugal/PT0695/2020 | Europe / Portugal |  | 2020-03-14 | EPI_ISL_510954 |
| Portugal/PT0696/2020 | Europe / Portugal |  | 2020-03-14 | EPI_ISL_510955 |
| Portugal/PT0697/2020 | Europe / Portugal |  | 2020-03-14 | EPI_ISL_510956 |
| Portugal/PT0698/2020 | Europe / Portugal |  | 2020-03-14 | EPI_ISL_510957 |
| Portugal/PT0699/2020 | Europe / Portugal |  | 2020-03-14 | EPI_ISL_510958 |
| Portugal/PT0776/2020 | Europe / Portugal |  | 2020-03-14 | EPI_ISL_511035 |
| Portugal/PT0779/2020 | Europe / Portugal |  | 2020-03-14 | EPI_ISL_511038 |
| Portugal/PT0780/2020 | Europe / Portugal |  | 2020-03-14 | EPI_ISL_511039 |
| Portugal/PT1478a/2020 | Europe / Portugal |  | 2020-03-14 | EPI_ISL_511437 |
| Portugal/PT1519/2020 | Europe / Portugal |  | 2020-03-14 | EPI_ISL_511478 |
| Portugal/PT0024/2020 | Europe / Portugal |  | 2020-03-15 | EPI_ISL_418009 |
| Portugal/PT0034/2020 | Europe / Portugal |  | 2020-03-15 | EPI_ISL_418019 |
| Portugal/PT0037/2020 | Europe / Portugal |  | 2020-03-15 | EPI_ISL_418022 |
| Portugal/PT0308/2020 | Europe / Portugal | yes | 2020-03-15 | EPI_ISL_454024 |
| Portugal/PT0313/2020 | Europe / Portugal | yes | 2020-03-15 | EPI_ISL_454029 |
| Portugal/PT0317/2020 | Europe / Portugal |  | 2020-03-15 | EPI_ISL_454033 |
| Portugal/PT0319/2020 | Europe / Portugal | yes | 2020-03-15 | EPI_ISL_454035 |
| Portugal/PT0320/2020 | Europe / Portugal |  | 2020-03-15 | EPI_ISL_454036 |
| Portugal/PT0321/2020 | Europe / Portugal | yes | 2020-03-15 | EPI_ISL_454037 |
| Portugal/PT0323/2020 | Europe / Portugal |  | 2020-03-15 | EPI_ISL_454039 |
| Portugal/PT0324/2020 | Europe / Portugal | yes | 2020-03-15 | EPI_ISL_454040 |
| Portugal/PT0329/2020 | Europe / Portugal |  | 2020-03-15 | EPI_ISL_454045 |
| Portugal/PT0330/2020 | Europe / Portugal |  | 2020-03-15 | EPI_ISL_454046 |
| Portugal/PT0501/2020 | Europe / Portugal |  | 2020-03-15 | EPI_ISL_454225 |
| Portugal/PT0611/2020 | Europe / Portugal |  | 2020-03-15 | EPI_ISL_454334 |
| Portugal/PT0612/2020 | Europe / Portugal |  | 2020-03-15 | EPI_ISL_454335 |
| Portugal/PT0613/2020 | Europe / Portugal | yes | 2020-03-15 | EPI_ISL_454336 |
| Portugal/PT0781/2020 | Europe / Portugal | yes | 2020-03-15 | EPI_ISL_511040 |
| Portugal/PT0782/2020 | Europe / Portugal |  | 2020-03-15 | EPI_ISL_511041 |
| Portugal/PT1475/2020 | Europe / Portugal |  | 2020-03-15 | EPI_ISL_511434 |
| Portugal/PT1493/2020 | Europe / Portugal | yes | 2020-03-15 | EPI_ISL_511452 |
| Portugal/PT1501/2020 | Europe / Portugal | yes | 2020-03-15 | EPI_ISL_511460 |
| Portugal/PT1514/2020 | Europe / Portugal |  | 2020-03-15 | EPI_ISL_511473 |
| Portugal/PT1515/2020 | Europe / Portugal |  | 2020-03-15 | EPI_ISL_511474 |
| Portugal/PT0036a/2020 | Europe / Portugal |  | 2020-03-15 | EPI_ISL_511189 |
| Portugal/PT0032/2020 | Europe / Portugal |  | 2020-03-16 | EPI_ISL_418017 |
| Portugal/PT0033/2020 | Europe / Portugal |  | 2020-03-16 | EPI_ISL_418018 |
| Portugal/PT0035/2020 | Europe / Portugal |  | 2020-03-16 | EPI_ISL_418020 |
| Portugal/PT0038/2020 | Europe / Portugal |  | 2020-03-16 | EPI_ISL_418023 |
| Portugal/PT0302/2020 | Europe / Portugal | yes | 2020-03-16 | EPI_ISL_454018 |
| Portugal/PT0314/2020 | Europe / Portugal | yes | 2020-03-16 | EPI_ISL_454030 |
| Portugal/PT0318/2020 | Europe / Portugal | yes | 2020-03-16 | EPI_ISL_454034 |
| Portugal/PT0322/2020 | Europe / Portugal | yes | 2020-03-16 | EPI_ISL_454038 |
| Portugal/PT0325/2020 | Europe / Portugal |  | 2020-03-16 | EPI_ISL_454041 |
| Portugal/PT0326/2020 | Europe / Portugal |  | 2020-03-16 | EPI_ISL_454042 |
| Portugal/PT0327/2020 | Europe / Portugal | yes | 2020-03-16 | EPI_ISL_454043 |
| Portugal/PT0328/2020 | Europe / Portugal |  | 2020-03-16 | EPI_ISL_454044 |
| Portugal/PT0331/2020 | Europe / Portugal |  | 2020-03-16 | EPI_ISL_454047 |
| Portugal/PT0614/2020 | Europe / Portugal |  | 2020-03-16 | EPI_ISL_454337 |
| Portugal/PT0778/2020 | Europe / Portugal |  | 2020-03-16 | EPI_ISL_511037 |
| Portugal/PT0783/2020 | Europe / Portugal |  | 2020-03-16 | EPI_ISL_511042 |
| Portugal/PT0784/2020 | Europe / Portugal |  | 2020-03-16 | EPI_ISL_511043 |
| Portugal/PT1095/2020 | Europe / Portugal |  | 2020-03-16 | EPI_ISL_511642 |
| Portugal/PT1096/2020 | Europe / Portugal |  | 2020-03-16 | EPI_ISL_511643 |
| Portugal/PT1098/2020 | Europe / Portugal |  | 2020-03-16 | EPI_ISL_511645 |
| Portugal/PT1099/2020 | Europe / Portugal |  | 2020-03-16 | EPI_ISL_511646 |
| Portugal/PT1249/2020 | Europe / Portugal |  | 2020-03-16 | EPI_ISL_511210 |
| Portugal/PT1250/2020 | Europe / Portugal |  | 2020-03-16 | EPI_ISL_511211 |
| Portugal/PT1251/2020 | Europe / Portugal |  | 2020-03-16 | EPI_ISL_511212 |
| Portugal/PT1299/2020 | Europe / Portugal | yes | 2020-03-16 | EPI_ISL_511258 |
| Portugal/PT1301/2020 | Europe / Portugal |  | 2020-03-16 | EPI_ISL_511260 |
| Portugal/PT1415/2020 | Europe / Portugal |  | 2020-03-16 | EPI_ISL_511374 |
| Portugal/PT1426/2020 | Europe / Portugal | yes | 2020-03-16 | EPI_ISL_511385 |
| Portugal/PT1430/2020 | Europe / Portugal |  | 2020-03-16 | EPI_ISL_511389 |
| Portugal/PT1467/2020 | Europe / Portugal |  | 2020-03-16 | EPI_ISL_511426 |
| Portugal/PT1468/2020 | Europe / Portugal |  | 2020-03-16 | EPI_ISL_511427 |
| Portugal/PT1482/2020 | Europe / Portugal |  | 2020-03-16 | EPI_ISL_511441 |
| Portugal/PT1500/2020 | Europe / Portugal |  | 2020-03-16 | EPI_ISL_511459 |
| Portugal/PT1503/2020 | Europe / Portugal |  | 2020-03-16 | EPI_ISL_511462 |
| Portugal/PT1506/2020 | Europe / Portugal |  | 2020-03-16 | EPI_ISL_511465 |
| Portugal/PT1518/2020 | Europe / Portugal | yes | 2020-03-16 | EPI_ISL_511477 |
| Portugal/PT0039/2020 | Europe / Portugal |  | 2020-03-17 | EPI_ISL_418024 |
| Portugal/PT0040/2020 | Europe / Portugal |  | 2020-03-17 | EPI_ISL_418025 |
| Portugal/PT0041/2020 | Europe / Portugal |  | 2020-03-17 | EPI_ISL_418026 |
| Portugal/PT0042/2020 | Europe / Portugal |  | 2020-03-17 | EPI_ISL_418027 |
| Portugal/PT0061/2020 | Europe / Portugal |  | 2020-03-17 | EPI_ISL_421464 |
| Portugal/PT0109/2020 | Europe / Portugal | yes | 2020-03-17 | EPI_ISL_453825 |
| Portugal/PT0335/2020 | Europe / Portugal |  | 2020-03-17 | EPI_ISL_454051 |
| Portugal/PT0339/2020 | Europe / Portugal | yes | 2020-03-17 | EPI_ISL_454055 |
| Portugal/PT0343/2020 | Europe / Portugal |  | 2020-03-17 | EPI_ISL_454059 |
| Portugal/PT0348/2020 | Europe / Portugal |  | 2020-03-17 | EPI_ISL_454064 |
| Portugal/PT0502/2020 | Europe / Portugal |  | 2020-03-17 | EPI_ISL_454226 |
| Portugal/PT0503/2020 | Europe / Portugal |  | 2020-03-17 | EPI_ISL_454227 |
| Portugal/PT0504/2020 | Europe / Portugal |  | 2020-03-17 | EPI_ISL_454228 |
| Portugal/PT0615/2020 | Europe / Portugal |  | 2020-03-17 | EPI_ISL_454338 |
| Portugal/PT0616/2020 | Europe / Portugal |  | 2020-03-17 | EPI_ISL_454339 |
| Portugal/PT0617/2020 | Europe / Portugal |  | 2020-03-17 | EPI_ISL_454340 |
| Portugal/PT0618/2020 | Europe / Portugal |  | 2020-03-17 | EPI_ISL_454341 |
| Portugal/PT0700/2020 | Europe / Portugal |  | 2020-03-17 | EPI_ISL_510959 |
| Portugal/PT0701/2020 | Europe / Portugal |  | 2020-03-17 | EPI_ISL_510960 |
| Portugal/PT0702/2020 | Europe / Portugal |  | 2020-03-17 | EPI_ISL_510961 |
| Portugal/PT0703/2020 | Europe / Portugal |  | 2020-03-17 | EPI_ISL_510962 |
| Portugal/PT0785/2020 | Europe / Portugal |  | 2020-03-17 | EPI_ISL_511044 |
| Portugal/PT0786/2020 | Europe / Portugal |  | 2020-03-17 | EPI_ISL_511045 |
| Portugal/PT0787/2020 | Europe / Portugal |  | 2020-03-17 | EPI_ISL_511046 |
| Portugal/PT0788/2020 | Europe / Portugal |  | 2020-03-17 | EPI_ISL_511047 |
| Portugal/PT0789/2020 | Europe / Portugal |  | 2020-03-17 | EPI_ISL_511048 |
| Portugal/PT0790/2020 | Europe / Portugal |  | 2020-03-17 | EPI_ISL_511049 |
| Portugal/PT1252/2020 | Europe / Portugal |  | 2020-03-17 | EPI_ISL_511213 |
| Portugal/PT1253/2020 | Europe / Portugal |  | 2020-03-17 | EPI_ISL_511214 |
| Portugal/PT1254/2020 | Europe / Portugal |  | 2020-03-17 | EPI_ISL_511215 |
| Portugal/PT1298/2020 | Europe / Portugal | yes | 2020-03-17 | EPI_ISL_511257 |
| Portugal/PT1300/2020 | Europe / Portugal |  | 2020-03-17 | EPI_ISL_511259 |
| Portugal/PT1302/2020 | Europe / Portugal |  | 2020-03-17 | EPI_ISL_511261 |
| Portugal/PT1303/2020 | Europe / Portugal |  | 2020-03-17 | EPI_ISL_511262 |
| Portugal/PT1304/2020 | Europe / Portugal |  | 2020-03-17 | EPI_ISL_511263 |
| Portugal/PT1305/2020 | Europe / Portugal |  | 2020-03-17 | EPI_ISL_511264 |
| Portugal/PT1306/2020 | Europe / Portugal |  | 2020-03-17 | EPI_ISL_511265 |
| Portugal/PT1307/2020 | Europe / Portugal |  | 2020-03-17 | EPI_ISL_511266 |
| Portugal/PT1308/2020 | Europe / Portugal |  | 2020-03-17 | EPI_ISL_511267 |
| Portugal/PT1309/2020 | Europe / Portugal |  | 2020-03-17 | EPI_ISL_511268 |
| Portugal/PT1310/2020 | Europe / Portugal |  | 2020-03-17 | EPI_ISL_511269 |
| Portugal/PT1311/2020 | Europe / Portugal |  | 2020-03-17 | EPI_ISL_511270 |
| Portugal/PT1312/2020 | Europe / Portugal |  | 2020-03-17 | EPI_ISL_511271 |
| Portugal/PT1313/2020 | Europe / Portugal |  | 2020-03-17 | EPI_ISL_511272 |
| Portugal/PT1314/2020 | Europe / Portugal |  | 2020-03-17 | EPI_ISL_511273 |
| Portugal/PT1315/2020 | Europe / Portugal |  | 2020-03-17 | EPI_ISL_511274 |
| Portugal/PT1316/2020 | Europe / Portugal |  | 2020-03-17 | EPI_ISL_511275 |
| Portugal/PT1453/2020 | Europe / Portugal |  | 2020-03-17 | EPI_ISL_511412 |
| Portugal/PT1458/2020 | Europe / Portugal |  | 2020-03-17 | EPI_ISL_511417 |
| Portugal/PT1469/2020 | Europe / Portugal | yes | 2020-03-17 | EPI_ISL_511428 |
| Portugal/PT1485/2020 | Europe / Portugal |  | 2020-03-17 | EPI_ISL_511444 |
| Portugal/PT1488/2020 | Europe / Portugal |  | 2020-03-17 | EPI_ISL_511447 |
| Portugal/PT1491/2020 | Europe / Portugal |  | 2020-03-17 | EPI_ISL_511450 |
| Portugal/PT1496/2020 | Europe / Portugal |  | 2020-03-17 | EPI_ISL_511455 |
| Portugal/PT1504/2020 | Europe / Portugal |  | 2020-03-17 | EPI_ISL_511463 |
| Portugal/PT1508/2020 | Europe / Portugal |  | 2020-03-17 | EPI_ISL_511467 |
| Portugal/PT1512/2020 | Europe / Portugal |  | 2020-03-17 | EPI_ISL_511471 |
| Portugal/PT0403/2020 | Europe / Portugal |  | 2020-03-17 | EPI_ISL_454127 |
| Portugal/PT0043/2020 | Europe / Portugal |  | 2020-03-18 | EPI_ISL_421446 |
| Portugal/PT0044/2020 | Europe / Portugal |  | 2020-03-18 | EPI_ISL_421447 |
| Portugal/PT0045/2020 | Europe / Portugal |  | 2020-03-18 | EPI_ISL_421448 |
| Portugal/PT0046/2020 | Europe / Portugal |  | 2020-03-18 | EPI_ISL_421449 |
| Portugal/PT0047/2020 | Europe / Portugal |  | 2020-03-18 | EPI_ISL_421450 |
| Portugal/PT0048/2020 | Europe / Portugal |  | 2020-03-18 | EPI_ISL_421451 |
| Portugal/PT0049/2020 | Europe / Portugal |  | 2020-03-18 | EPI_ISL_421452 |
| Portugal/PT0050/2020 | Europe / Portugal |  | 2020-03-18 | EPI_ISL_421453 |
| Portugal/PT0051/2020 | Europe / Portugal |  | 2020-03-18 | EPI_ISL_421454 |
| Portugal/PT0052/2020 | Europe / Portugal |  | 2020-03-18 | EPI_ISL_421455 |
| Portugal/PT0054/2020 | Europe / Portugal |  | 2020-03-18 | EPI_ISL_421457 |
| Portugal/PT0079/2020 | Europe / Portugal |  | 2020-03-18 | EPI_ISL_421482 |
| Portugal/PT0080/2020 | Europe / Portugal |  | 2020-03-18 | EPI_ISL_421483 |
| Portugal/PT0332/2020 | Europe / Portugal |  | 2020-03-18 | EPI_ISL_454048 |
| Portugal/PT0333/2020 | Europe / Portugal |  | 2020-03-18 | EPI_ISL_454049 |
| Portugal/PT0334/2020 | Europe / Portugal |  | 2020-03-18 | EPI_ISL_454050 |
| Portugal/PT0336/2020 | Europe / Portugal | yes | 2020-03-18 | EPI_ISL_454052 |
| Portugal/PT0337/2020 | Europe / Portugal |  | 2020-03-18 | EPI_ISL_454053 |
| Portugal/PT0338/2020 | Europe / Portugal |  | 2020-03-18 | EPI_ISL_454054 |
| Portugal/PT0340/2020 | Europe / Portugal | yes | 2020-03-18 | EPI_ISL_454056 |
| Portugal/PT0341/2020 | Europe / Portugal |  | 2020-03-18 | EPI_ISL_454057 |
| Portugal/PT0344/2020 | Europe / Portugal | yes | 2020-03-18 | EPI_ISL_454060 |
| Portugal/PT0345/2020 | Europe / Portugal |  | 2020-03-18 | EPI_ISL_454061 |
| Portugal/PT0346/2020 | Europe / Portugal |  | 2020-03-18 | EPI_ISL_454062 |
| Portugal/PT0347/2020 | Europe / Portugal | yes | 2020-03-18 | EPI_ISL_454063 |
| Portugal/PT0349/2020 | Europe / Portugal |  | 2020-03-18 | EPI_ISL_454065 |
| Portugal/PT0351/2020 | Europe / Portugal | yes | 2020-03-18 | EPI_ISL_454067 |
| Portugal/PT0352/2020 | Europe / Portugal | yes | 2020-03-18 | EPI_ISL_454068 |
| Portugal/PT0353/2020 | Europe / Portugal | yes | 2020-03-18 | EPI_ISL_454069 |
| Portugal/PT0354/2020 | Europe / Portugal |  | 2020-03-18 | EPI_ISL_454070 |
| Portugal/PT0355/2020 | Europe / Portugal |  | 2020-03-18 | EPI_ISL_454071 |
| Portugal/PT0404/2020 | Europe / Portugal |  | 2020-03-18 | EPI_ISL_454128 |
| Portugal/PT0505/2020 | Europe / Portugal |  | 2020-03-18 | EPI_ISL_454229 |
| Portugal/PT0619/2020 | Europe / Portugal |  | 2020-03-18 | EPI_ISL_454342 |
| Portugal/PT0729/2020 | Europe / Portugal |  | 2020-03-18 | EPI_ISL_510988 |
| Portugal/PT0791/2020 | Europe / Portugal |  | 2020-03-18 | EPI_ISL_511050 |
| Portugal/PT0792/2020 | Europe / Portugal | yes | 2020-03-18 | EPI_ISL_511051 |
| Portugal/PT0793/2020 | Europe / Portugal |  | 2020-03-18 | EPI_ISL_511052 |
| Portugal/PT1255/2020 | Europe / Portugal |  | 2020-03-18 | EPI_ISL_511216 |
| Portugal/PT1256/2020 | Europe / Portugal |  | 2020-03-18 | EPI_ISL_511217 |
| Portugal/PT1257/2020 | Europe / Portugal |  | 2020-03-18 | EPI_ISL_511218 |
| Portugal/PT1258/2020 | Europe / Portugal |  | 2020-03-18 | EPI_ISL_511219 |
| Portugal/PT1259/2020 | Europe / Portugal |  | 2020-03-18 | EPI_ISL_511220 |
| Portugal/PT1260/2020 | Europe / Portugal |  | 2020-03-18 | EPI_ISL_511221 |
| Portugal/PT1317/2020 | Europe / Portugal | yes | 2020-03-18 | EPI_ISL_511276 |
| Portugal/PT1318/2020 | Europe / Portugal |  | 2020-03-18 | EPI_ISL_511277 |
| Portugal/PT1319/2020 | Europe / Portugal |  | 2020-03-18 | EPI_ISL_511278 |
| Portugal/PT1320/2020 | Europe / Portugal |  | 2020-03-18 | EPI_ISL_511279 |
| Portugal/PT1321/2020 | Europe / Portugal |  | 2020-03-18 | EPI_ISL_511280 |
| Portugal/PT1322/2020 | Europe / Portugal | yes | 2020-03-18 | EPI_ISL_511281 |
| Portugal/PT1323/2020 | Europe / Portugal |  | 2020-03-18 | EPI_ISL_511282 |
| Portugal/PT1324/2020 | Europe / Portugal |  | 2020-03-18 | EPI_ISL_511283 |
| Portugal/PT1325/2020 | Europe / Portugal |  | 2020-03-18 | EPI_ISL_511284 |
| Portugal/PT1326/2020 | Europe / Portugal | yes | 2020-03-18 | EPI_ISL_511285 |
| Portugal/PT1327/2020 | Europe / Portugal | yes | 2020-03-18 | EPI_ISL_511286 |
| Portugal/PT1328/2020 | Europe / Portugal |  | 2020-03-18 | EPI_ISL_511287 |
| Portugal/PT1329/2020 | Europe / Portugal | yes | 2020-03-18 | EPI_ISL_511288 |
| Portugal/PT1330/2020 | Europe / Portugal | yes | 2020-03-18 | EPI_ISL_511289 |
| Portugal/PT1331/2020 | Europe / Portugal |  | 2020-03-18 | EPI_ISL_511290 |
| Portugal/PT1334/2020 | Europe / Portugal | yes | 2020-03-18 | EPI_ISL_511293 |
| Portugal/PT1335/2020 | Europe / Portugal |  | 2020-03-18 | EPI_ISL_511294 |
| Portugal/PT1336/2020 | Europe / Portugal |  | 2020-03-18 | EPI_ISL_511295 |
| Portugal/PT1413/2020 | Europe / Portugal |  | 2020-03-18 | EPI_ISL_511372 |
| Portugal/PT1470/2020 | Europe / Portugal |  | 2020-03-18 | EPI_ISL_511429 |
| Portugal/PT1513/2020 | Europe / Portugal |  | 2020-03-18 | EPI_ISL_511472 |
| Portugal/PT1520/2020 | Europe / Portugal |  | 2020-03-18 | EPI_ISL_511479 |
| Portugal/PT0053/2020 | Europe / Portugal |  | 2020-03-19 | EPI_ISL_421456 |
| Portugal/PT0055/2020 | Europe / Portugal |  | 2020-03-19 | EPI_ISL_421458 |
| Portugal/PT0062/2020 | Europe / Portugal |  | 2020-03-19 | EPI_ISL_421465 |
| Portugal/PT0081/2020 | Europe / Portugal |  | 2020-03-19 | EPI_ISL_421484 |
| Portugal/PT0350/2020 | Europe / Portugal | yes | 2020-03-19 | EPI_ISL_454066 |
| Portugal/PT0356/2020 | Europe / Portugal |  | 2020-03-19 | EPI_ISL_454072 |
| Portugal/PT0357/2020 | Europe / Portugal |  | 2020-03-19 | EPI_ISL_454073 |
| Portugal/PT0358/2020 | Europe / Portugal |  | 2020-03-19 | EPI_ISL_454074 |
| Portugal/PT0359/2020 | Europe / Portugal |  | 2020-03-19 | EPI_ISL_454075 |
| Portugal/PT0360/2020 | Europe / Portugal |  | 2020-03-19 | EPI_ISL_454076 |
| Portugal/PT0361/2020 | Europe / Portugal | yes | 2020-03-19 | EPI_ISL_454077 |
| Portugal/PT0362/2020 | Europe / Portugal |  | 2020-03-19 | EPI_ISL_454078 |
| Portugal/PT0363/2020 | Europe / Portugal |  | 2020-03-19 | EPI_ISL_454079 |
| Portugal/PT0364/2020 | Europe / Portugal |  | 2020-03-19 | EPI_ISL_454080 |
| Portugal/PT0365/2020 | Europe / Portugal | yes | 2020-03-19 | EPI_ISL_454081 |
| Portugal/PT0366/2020 | Europe / Portugal |  | 2020-03-19 | EPI_ISL_454082 |
| Portugal/PT0367/2020 | Europe / Portugal |  | 2020-03-19 | EPI_ISL_454083 |
| Portugal/PT0368/2020 | Europe / Portugal |  | 2020-03-19 | EPI_ISL_454084 |
| Portugal/PT0369/2020 | Europe / Portugal |  | 2020-03-19 | EPI_ISL_454085 |
| Portugal/PT0370/2020 | Europe / Portugal |  | 2020-03-19 | EPI_ISL_454086 |
| Portugal/PT0371/2020 | Europe / Portugal |  | 2020-03-19 | EPI_ISL_454087 |
| Portugal/PT0373/2020 | Europe / Portugal |  | 2020-03-19 | EPI_ISL_454089 |
| Portugal/PT0374/2020 | Europe / Portugal |  | 2020-03-19 | EPI_ISL_454090 |
| Portugal/PT0375/2020 | Europe / Portugal |  | 2020-03-19 | EPI_ISL_454091 |
| Portugal/PT0383/2020 | Europe / Portugal |  | 2020-03-19 | EPI_ISL_454099 |
| Portugal/PT0434/2020 | Europe / Portugal |  | 2020-03-19 | EPI_ISL_454158 |
| Portugal/PT0506/2020 | Europe / Portugal |  | 2020-03-19 | EPI_ISL_454230 |
| Portugal/PT0507/2020 | Europe / Portugal |  | 2020-03-19 | EPI_ISL_454231 |
| Portugal/PT0508/2020 | Europe / Portugal |  | 2020-03-19 | EPI_ISL_454232 |
| Portugal/PT0509/2020 | Europe / Portugal |  | 2020-03-19 | EPI_ISL_454233 |
| Portugal/PT0510/2020 | Europe / Portugal |  | 2020-03-19 | EPI_ISL_454234 |
| Portugal/PT0511/2020 | Europe / Portugal |  | 2020-03-19 | EPI_ISL_454235 |
| Portugal/PT0595/2020 | Europe / Portugal |  | 2020-03-19 | EPI_ISL_454318 |
| Portugal/PT0670/2020 | Europe / Portugal |  | 2020-03-19 | EPI_ISL_510929 |
| Portugal/PT0730/2020 | Europe / Portugal |  | 2020-03-19 | EPI_ISL_510989 |
| Portugal/PT0775/2020 | Europe / Portugal |  | 2020-03-19 | EPI_ISL_511034 |
| Portugal/PT0794/2020 | Europe / Portugal |  | 2020-03-19 | EPI_ISL_511053 |
| Portugal/PT0795/2020 | Europe / Portugal |  | 2020-03-19 | EPI_ISL_511054 |
| Portugal/PT0796/2020 | Europe / Portugal |  | 2020-03-19 | EPI_ISL_511055 |
| Portugal/PT0797/2020 | Europe / Portugal |  | 2020-03-19 | EPI_ISL_511056 |
| Portugal/PT0798/2020 | Europe / Portugal |  | 2020-03-19 | EPI_ISL_511057 |
| Portugal/PT0799/2020 | Europe / Portugal |  | 2020-03-19 | EPI_ISL_511058 |
| Portugal/PT0800/2020 | Europe / Portugal |  | 2020-03-19 | EPI_ISL_511059 |
| Portugal/PT0801/2020 | Europe / Portugal |  | 2020-03-19 | EPI_ISL_511060 |
| Portugal/PT0803/2020 | Europe / Portugal |  | 2020-03-19 | EPI_ISL_511062 |
| Portugal/PT0804/2020 | Europe / Portugal |  | 2020-03-19 | EPI_ISL_511063 |
| Portugal/PT0805/2020 | Europe / Portugal |  | 2020-03-19 | EPI_ISL_511064 |
| Portugal/PT0806/2020 | Europe / Portugal |  | 2020-03-19 | EPI_ISL_511065 |
| Portugal/PT0886/2020 | Europe / Portugal |  | 2020-03-19 | EPI_ISL_511144 |
| Portugal/PT0887/2020 | Europe / Portugal |  | 2020-03-19 | EPI_ISL_511145 |
| Portugal/PT0888/2020 | Europe / Portugal |  | 2020-03-19 | EPI_ISL_511146 |
| Portugal/PT1261/2020 | Europe / Portugal |  | 2020-03-19 | EPI_ISL_511222 |
| Portugal/PT1262/2020 | Europe / Portugal |  | 2020-03-19 | EPI_ISL_511223 |
| Portugal/PT1263/2020 | Europe / Portugal | yes | 2020-03-19 | EPI_ISL_511224 |
| Portugal/PT1264/2020 | Europe / Portugal |  | 2020-03-19 | EPI_ISL_511225 |
| Portugal/PT1265/2020 | Europe / Portugal |  | 2020-03-19 | EPI_ISL_511226 |
| Portugal/PT1266/2020 | Europe / Portugal |  | 2020-03-19 | EPI_ISL_511227 |
| Portugal/PT1267/2020 | Europe / Portugal |  | 2020-03-19 | EPI_ISL_511228 |
| Portugal/PT1268/2020 | Europe / Portugal |  | 2020-03-19 | EPI_ISL_511229 |
| Portugal/PT1269/2020 | Europe / Portugal |  | 2020-03-19 | EPI_ISL_511230 |
| Portugal/PT1332/2020 | Europe / Portugal |  | 2020-03-19 | EPI_ISL_511291 |
| Portugal/PT1333/2020 | Europe / Portugal |  | 2020-03-19 | EPI_ISL_511292 |
| Portugal/PT1337/2020 | Europe / Portugal |  | 2020-03-19 | EPI_ISL_511296 |
| Portugal/PT1338/2020 | Europe / Portugal |  | 2020-03-19 | EPI_ISL_511297 |
| Portugal/PT1339/2020 | Europe / Portugal |  | 2020-03-19 | EPI_ISL_511298 |
| Portugal/PT1340/2020 | Europe / Portugal | yes | 2020-03-19 | EPI_ISL_511299 |
| Portugal/PT1341/2020 | Europe / Portugal | yes | 2020-03-19 | EPI_ISL_511300 |
| Portugal/PT1342/2020 | Europe / Portugal |  | 2020-03-19 | EPI_ISL_511301 |
| Portugal/PT1343/2020 | Europe / Portugal |  | 2020-03-19 | EPI_ISL_511302 |
| Portugal/PT1344/2020 | Europe / Portugal |  | 2020-03-19 | EPI_ISL_511303 |
| Portugal/PT1345/2020 | Europe / Portugal |  | 2020-03-19 | EPI_ISL_511304 |
| Portugal/PT1481/2020 | Europe / Portugal |  | 2020-03-19 | EPI_ISL_511440 |
| Portugal/PT1487/2020 | Europe / Portugal |  | 2020-03-19 | EPI_ISL_511446 |
| Portugal/PT1489/2020 | Europe / Portugal |  | 2020-03-19 | EPI_ISL_511448 |
| Portugal/PT0056/2020 | Europe / Portugal |  | 2020-03-20 | EPI_ISL_421459 |
| Portugal/PT0057/2020 | Europe / Portugal |  | 2020-03-20 | EPI_ISL_421460 |
| Portugal/PT0058/2020 | Europe / Portugal |  | 2020-03-20 | EPI_ISL_421461 |
| Portugal/PT0059/2020 | Europe / Portugal |  | 2020-03-20 | EPI_ISL_421462 |
| Portugal/PT0060/2020 | Europe / Portugal |  | 2020-03-20 | EPI_ISL_421463 |
| Portugal/PT0063/2020 | Europe / Portugal |  | 2020-03-20 | EPI_ISL_421466 |
| Portugal/PT0064/2020 | Europe / Portugal |  | 2020-03-20 | EPI_ISL_421467 |
| Portugal/PT0086/2020 | Europe / Portugal |  | 2020-03-20 | EPI_ISL_421489 |
| Portugal/PT0091/2020 | Europe / Portugal |  | 2020-03-20 | EPI_ISL_421494 |
| Portugal/PT0105/2020 | Europe / Portugal |  | 2020-03-20 | EPI_ISL_453821 |
| Portugal/PT0372/2020 | Europe / Portugal | yes | 2020-03-20 | EPI_ISL_454088 |
| Portugal/PT0376/2020 | Europe / Portugal |  | 2020-03-20 | EPI_ISL_454092 |
| Portugal/PT0377/2020 | Europe / Portugal |  | 2020-03-20 | EPI_ISL_454093 |
| Portugal/PT0378/2020 | Europe / Portugal |  | 2020-03-20 | EPI_ISL_454094 |
| Portugal/PT0379/2020 | Europe / Portugal | yes | 2020-03-20 | EPI_ISL_454095 |
| Portugal/PT0380/2020 | Europe / Portugal | yes | 2020-03-20 | EPI_ISL_454096 |
| Portugal/PT0381/2020 | Europe / Portugal |  | 2020-03-20 | EPI_ISL_454097 |
| Portugal/PT0382/2020 | Europe / Portugal | yes | 2020-03-20 | EPI_ISL_454098 |
| Portugal/PT0384/2020 | Europe / Portugal |  | 2020-03-20 | EPI_ISL_454100 |
| Portugal/PT0385/2020 | Europe / Portugal | yes | 2020-03-20 | EPI_ISL_454101 |
| Portugal/PT0386/2020 | Europe / Portugal |  | 2020-03-20 | EPI_ISL_454102 |
| Portugal/PT0387/2020 | Europe / Portugal |  | 2020-03-20 | EPI_ISL_454103 |
| Portugal/PT0388/2020 | Europe / Portugal |  | 2020-03-20 | EPI_ISL_454104 |
| Portugal/PT0405/2020 | Europe / Portugal |  | 2020-03-20 | EPI_ISL_454129 |
| Portugal/PT0435/2020 | Europe / Portugal |  | 2020-03-20 | EPI_ISL_454159 |
| Portugal/PT0512/2020 | Europe / Portugal |  | 2020-03-20 | EPI_ISL_454236 |
| Portugal/PT0513/2020 | Europe / Portugal |  | 2020-03-20 | EPI_ISL_454237 |
| Portugal/PT0514/2020 | Europe / Portugal |  | 2020-03-20 | EPI_ISL_454238 |
| Portugal/PT0515/2020 | Europe / Portugal |  | 2020-03-20 | EPI_ISL_454239 |
| Portugal/PT0516/2020 | Europe / Portugal |  | 2020-03-20 | EPI_ISL_454240 |
| Portugal/PT0704/2020 | Europe / Portugal |  | 2020-03-20 | EPI_ISL_510963 |
| Portugal/PT0732/2020 | Europe / Portugal |  | 2020-03-20 | EPI_ISL_510991 |
| Portugal/PT0777a/2020 | Europe / Portugal |  | 2020-03-20 | EPI_ISL_511036 |
| Portugal/PT0802/2020 | Europe / Portugal |  | 2020-03-20 | EPI_ISL_511061 |
| Portugal/PT0807/2020 | Europe / Portugal |  | 2020-03-20 | EPI_ISL_511066 |
| Portugal/PT0808/2020 | Europe / Portugal |  | 2020-03-20 | EPI_ISL_511067 |
| Portugal/PT0809/2020 | Europe / Portugal |  | 2020-03-20 | EPI_ISL_511068 |
| Portugal/PT0810/2020 | Europe / Portugal |  | 2020-03-20 | EPI_ISL_511069 |
| Portugal/PT0811/2020 | Europe / Portugal |  | 2020-03-20 | EPI_ISL_511070 |
| Portugal/PT0812/2020 | Europe / Portugal |  | 2020-03-20 | EPI_ISL_511071 |
| Portugal/PT0813/2020 | Europe / Portugal |  | 2020-03-20 | EPI_ISL_511072 |
| Portugal/PT0814/2020 | Europe / Portugal |  | 2020-03-20 | EPI_ISL_511073 |
| Portugal/PT0815/2020 | Europe / Portugal |  | 2020-03-20 | EPI_ISL_511074 |
| Portugal/PT0816/2020 | Europe / Portugal |  | 2020-03-20 | EPI_ISL_511075 |
| Portugal/PT0817/2020 | Europe / Portugal |  | 2020-03-20 | EPI_ISL_511076 |
| Portugal/PT0818/2020 | Europe / Portugal |  | 2020-03-20 | EPI_ISL_511077 |
| Portugal/PT0819/2020 | Europe / Portugal |  | 2020-03-20 | EPI_ISL_511078 |
| Portugal/PT0820/2020 | Europe / Portugal |  | 2020-03-20 | EPI_ISL_511079 |
| Portugal/PT0821/2020 | Europe / Portugal |  | 2020-03-20 | EPI_ISL_511080 |
| Portugal/PT0822/2020 | Europe / Portugal |  | 2020-03-20 | EPI_ISL_511081 |
| Portugal/PT0823/2020 | Europe / Portugal |  | 2020-03-20 | EPI_ISL_511082 |
| Portugal/PT0824/2020 | Europe / Portugal |  | 2020-03-20 | EPI_ISL_511083 |
| Portugal/PT0889/2020 | Europe / Portugal |  | 2020-03-20 | EPI_ISL_511147 |
| Portugal/PT0890/2020 | Europe / Portugal |  | 2020-03-20 | EPI_ISL_511148 |
| Portugal/PT0891/2020 | Europe / Portugal |  | 2020-03-20 | EPI_ISL_511149 |
| Portugal/PT0892/2020 | Europe / Portugal |  | 2020-03-20 | EPI_ISL_511150 |
| Portugal/PT0893/2020 | Europe / Portugal |  | 2020-03-20 | EPI_ISL_511151 |
| Portugal/PT0894/2020 | Europe / Portugal |  | 2020-03-20 | EPI_ISL_511152 |
| Portugal/PT0895/2020 | Europe / Portugal |  | 2020-03-20 | EPI_ISL_511153 |
| Portugal/PT1138/2020 | Europe / Portugal |  | 2020-03-20 | EPI_ISL_511685 |
| Portugal/PT1170/2020 | Europe / Portugal |  | 2020-03-20 | EPI_ISL_511716 |
| Portugal/PT1171/2020 | Europe / Portugal |  | 2020-03-20 | EPI_ISL_511717 |
| Portugal/PT1172/2020 | Europe / Portugal |  | 2020-03-20 | EPI_ISL_511718 |
| Portugal/PT1270/2020 | Europe / Portugal |  | 2020-03-20 | EPI_ISL_511231 |
| Portugal/PT1271/2020 | Europe / Portugal |  | 2020-03-20 | EPI_ISL_511232 |
| Portugal/PT1272/2020 | Europe / Portugal |  | 2020-03-20 | EPI_ISL_511233 |
| Portugal/PT1474/2020 | Europe / Portugal |  | 2020-03-20 | EPI_ISL_511433 |
| Portugal/PT1499/2020 | Europe / Portugal | yes | 2020-03-20 | EPI_ISL_511458 |
| Portugal/PT0065/2020 | Europe / Portugal |  | 2020-03-21 | EPI_ISL_421468 |
| Portugal/PT0066/2020 | Europe / Portugal |  | 2020-03-21 | EPI_ISL_421469 |
| Portugal/PT0067/2020 | Europe / Portugal |  | 2020-03-21 | EPI_ISL_421470 |
| Portugal/PT0068/2020 | Europe / Portugal |  | 2020-03-21 | EPI_ISL_421471 |
| Portugal/PT0069/2020 | Europe / Portugal |  | 2020-03-21 | EPI_ISL_421472 |
| Portugal/PT0070/2020 | Europe / Portugal |  | 2020-03-21 | EPI_ISL_421473 |
| Portugal/PT0071/2020 | Europe / Portugal |  | 2020-03-21 | EPI_ISL_421474 |
| Portugal/PT0072/2020 | Europe / Portugal |  | 2020-03-21 | EPI_ISL_421475 |
| Portugal/PT0073/2020 | Europe / Portugal |  | 2020-03-21 | EPI_ISL_421476 |
| Portugal/PT0074/2020 | Europe / Portugal |  | 2020-03-21 | EPI_ISL_421477 |
| Portugal/PT0075/2020 | Europe / Portugal |  | 2020-03-21 | EPI_ISL_421478 |
| Portugal/PT0076/2020 | Europe / Portugal |  | 2020-03-21 | EPI_ISL_421479 |
| Portugal/PT0077/2020 | Europe / Portugal |  | 2020-03-21 | EPI_ISL_421480 |
| Portugal/PT0078/2020 | Europe / Portugal |  | 2020-03-21 | EPI_ISL_421481 |
| Portugal/PT0090/2020 | Europe / Portugal |  | 2020-03-21 | EPI_ISL_421493 |
| Portugal/PT0103/2020 | Europe / Portugal |  | 2020-03-21 | EPI_ISL_453819 |
| Portugal/PT0104/2020 | Europe / Portugal |  | 2020-03-21 | EPI_ISL_453820 |
| Portugal/PT0106/2020 | Europe / Portugal |  | 2020-03-21 | EPI_ISL_453822 |
| Portugal/PT0107/2020 | Europe / Portugal |  | 2020-03-21 | EPI_ISL_453823 |
| Portugal/PT0108/2020 | Europe / Portugal |  | 2020-03-21 | EPI_ISL_453824 |
| Portugal/PT0150/2020 | Europe / Portugal |  | 2020-03-21 | EPI_ISL_453866 |
| Portugal/PT0406/2020 | Europe / Portugal |  | 2020-03-21 | EPI_ISL_454130 |
| Portugal/PT0407/2020 | Europe / Portugal |  | 2020-03-21 | EPI_ISL_454131 |
| Portugal/PT0408/2020 | Europe / Portugal |  | 2020-03-21 | EPI_ISL_454132 |
| Portugal/PT0409/2020 | Europe / Portugal |  | 2020-03-21 | EPI_ISL_454133 |
| Portugal/PT0410/2020 | Europe / Portugal |  | 2020-03-21 | EPI_ISL_454134 |
| Portugal/PT0411/2020 | Europe / Portugal |  | 2020-03-21 | EPI_ISL_454135 |
| Portugal/PT0436/2020 | Europe / Portugal |  | 2020-03-21 | EPI_ISL_454160 |
| Portugal/PT0517/2020 | Europe / Portugal |  | 2020-03-21 | EPI_ISL_454241 |
| Portugal/PT0518/2020 | Europe / Portugal |  | 2020-03-21 | EPI_ISL_454242 |
| Portugal/PT0705/2020 | Europe / Portugal |  | 2020-03-21 | EPI_ISL_510964 |
| Portugal/PT0706/2020 | Europe / Portugal |  | 2020-03-21 | EPI_ISL_510965 |
| Portugal/PT0731/2020 | Europe / Portugal | yes | 2020-03-21 | EPI_ISL_510990 |
| Portugal/PT0733/2020 | Europe / Portugal | yes | 2020-03-21 | EPI_ISL_510992 |
| Portugal/PT0734/2020 | Europe / Portugal | yes | 2020-03-21 | EPI_ISL_510993 |
| Portugal/PT0735/2020 | Europe / Portugal |  | 2020-03-21 | EPI_ISL_510994 |
| Portugal/PT0736/2020 | Europe / Portugal | yes | 2020-03-21 | EPI_ISL_510995 |
| Portugal/PT0737/2020 | Europe / Portugal |  | 2020-03-21 | EPI_ISL_510996 |
| Portugal/PT0740/2020 | Europe / Portugal |  | 2020-03-21 | EPI_ISL_510999 |
| Portugal/PT0825/2020 | Europe / Portugal |  | 2020-03-21 | EPI_ISL_511084 |
| Portugal/PT0826/2020 | Europe / Portugal |  | 2020-03-21 | EPI_ISL_511085 |
| Portugal/PT0827/2020 | Europe / Portugal |  | 2020-03-21 | EPI_ISL_511086 |
| Portugal/PT0828/2020 | Europe / Portugal |  | 2020-03-21 | EPI_ISL_511087 |
| Portugal/PT0829/2020 | Europe / Portugal |  | 2020-03-21 | EPI_ISL_511088 |
| Portugal/PT0830/2020 | Europe / Portugal |  | 2020-03-21 | EPI_ISL_511089 |
| Portugal/PT0831/2020 | Europe / Portugal |  | 2020-03-21 | EPI_ISL_511090 |
| Portugal/PT0832/2020 | Europe / Portugal |  | 2020-03-21 | EPI_ISL_511091 |
| Portugal/PT0993/2020 | Europe / Portugal |  | 2020-03-21 | EPI_ISL_511541 |
| Portugal/PT0999/2020 | Europe / Portugal |  | 2020-03-21 | EPI_ISL_511547 |
| Portugal/PT1273/2020 | Europe / Portugal |  | 2020-03-21 | EPI_ISL_511234 |
| Portugal/PT1274/2020 | Europe / Portugal |  | 2020-03-21 | EPI_ISL_511235 |
| Portugal/PT1275/2020 | Europe / Portugal |  | 2020-03-21 | EPI_ISL_511236 |
| Portugal/PT1276/2020 | Europe / Portugal |  | 2020-03-21 | EPI_ISL_511237 |
| Portugal/PT1438/2020 | Europe / Portugal |  | 2020-03-21 | EPI_ISL_511397 |
| Portugal/PT0082/2020 | Europe / Portugal |  | 2020-03-22 | EPI_ISL_421485 |
| Portugal/PT0083/2020 | Europe / Portugal |  | 2020-03-22 | EPI_ISL_421486 |
| Portugal/PT0084/2020 | Europe / Portugal |  | 2020-03-22 | EPI_ISL_421487 |
| Portugal/PT0085/2020 | Europe / Portugal |  | 2020-03-22 | EPI_ISL_421488 |
| Portugal/PT0087/2020 | Europe / Portugal |  | 2020-03-22 | EPI_ISL_421490 |
| Portugal/PT0088/2020 | Europe / Portugal |  | 2020-03-22 | EPI_ISL_421491 |
| Portugal/PT0089/2020 | Europe / Portugal |  | 2020-03-22 | EPI_ISL_421492 |
| Portugal/PT0412/2020 | Europe / Portugal |  | 2020-03-22 | EPI_ISL_454136 |
| Portugal/PT0437/2020 | Europe / Portugal |  | 2020-03-22 | EPI_ISL_454161 |
| Portugal/PT0438/2020 | Europe / Portugal |  | 2020-03-22 | EPI_ISL_454162 |
| Portugal/PT0439/2020 | Europe / Portugal |  | 2020-03-22 | EPI_ISL_454163 |
| Portugal/PT0519/2020 | Europe / Portugal |  | 2020-03-22 | EPI_ISL_454243 |
| Portugal/PT0520/2020 | Europe / Portugal |  | 2020-03-22 | EPI_ISL_454244 |
| Portugal/PT0707/2020 | Europe / Portugal |  | 2020-03-22 | EPI_ISL_510966 |
| Portugal/PT0738/2020 | Europe / Portugal |  | 2020-03-22 | EPI_ISL_510997 |
| Portugal/PT0739/2020 | Europe / Portugal |  | 2020-03-22 | EPI_ISL_510998 |
| Portugal/PT0741/2020 | Europe / Portugal |  | 2020-03-22 | EPI_ISL_511000 |
| Portugal/PT0742/2020 | Europe / Portugal | yes | 2020-03-22 | EPI_ISL_511001 |
| Portugal/PT0743/2020 | Europe / Portugal |  | 2020-03-22 | EPI_ISL_511002 |
| Portugal/PT0744/2020 | Europe / Portugal |  | 2020-03-22 | EPI_ISL_511003 |
| Portugal/PT0745/2020 | Europe / Portugal | yes | 2020-03-22 | EPI_ISL_511004 |
| Portugal/PT0746/2020 | Europe / Portugal |  | 2020-03-22 | EPI_ISL_511005 |
| Portugal/PT0747a/2020 | Europe / Portugal |  | 2020-03-22 | EPI_ISL_511006 |
| Portugal/PT0748/2020 | Europe / Portugal |  | 2020-03-22 | EPI_ISL_511007 |
| Portugal/PT0749/2020 | Europe / Portugal |  | 2020-03-22 | EPI_ISL_511008 |
| Portugal/PT0833/2020 | Europe / Portugal |  | 2020-03-22 | EPI_ISL_511092 |
| Portugal/PT0834/2020 | Europe / Portugal |  | 2020-03-22 | EPI_ISL_511093 |
| Portugal/PT0835/2020 | Europe / Portugal |  | 2020-03-22 | EPI_ISL_511094 |
| Portugal/PT0836/2020 | Europe / Portugal |  | 2020-03-22 | EPI_ISL_511095 |
| Portugal/PT0837/2020 | Europe / Portugal |  | 2020-03-22 | EPI_ISL_511096 |
| Portugal/PT0838/2020 | Europe / Portugal |  | 2020-03-22 | EPI_ISL_511097 |
| Portugal/PT0839/2020 | Europe / Portugal |  | 2020-03-22 | EPI_ISL_511098 |
| Portugal/PT0840/2020 | Europe / Portugal |  | 2020-03-22 | EPI_ISL_511099 |
| Portugal/PT0841/2020 | Europe / Portugal |  | 2020-03-22 | EPI_ISL_511100 |
| Portugal/PT0842/2020 | Europe / Portugal |  | 2020-03-22 | EPI_ISL_511101 |
| Portugal/PT0843/2020 | Europe / Portugal |  | 2020-03-22 | EPI_ISL_511102 |
| Portugal/PT0981/2020 | Europe / Portugal |  | 2020-03-22 | EPI_ISL_511530 |
| Portugal/PT0982/2020 | Europe / Portugal |  | 2020-03-22 | EPI_ISL_582516 |
| Portugal/PT0983/2020 | Europe / Portugal |  | 2020-03-22 | EPI_ISL_511531 |
| Portugal/PT0984/2020 | Europe / Portugal |  | 2020-03-22 | EPI_ISL_511532 |
| Portugal/PT0985/2020 | Europe / Portugal |  | 2020-03-22 | EPI_ISL_511533 |
| Portugal/PT0992/2020 | Europe / Portugal |  | 2020-03-22 | EPI_ISL_511540 |
| Portugal/PT0994/2020 | Europe / Portugal | yes | 2020-03-22 | EPI_ISL_511542 |
| Portugal/PT0995/2020 | Europe / Portugal | yes | 2020-03-22 | EPI_ISL_511543 |
| Portugal/PT0996/2020 | Europe / Portugal |  | 2020-03-22 | EPI_ISL_511544 |
| Portugal/PT0997/2020 | Europe / Portugal |  | 2020-03-22 | EPI_ISL_511545 |
| Portugal/PT0998/2020 | Europe / Portugal | yes | 2020-03-22 | EPI_ISL_511546 |
| Portugal/PT1000/2020 | Europe / Portugal |  | 2020-03-22 | EPI_ISL_511548 |
| Portugal/PT1001/2020 | Europe / Portugal |  | 2020-03-22 | EPI_ISL_511549 |
| Portugal/PT1173/2020 | Europe / Portugal |  | 2020-03-22 | EPI_ISL_511719 |
| Portugal/PT1174/2020 | Europe / Portugal |  | 2020-03-22 | EPI_ISL_511720 |
| Portugal/PT0092/2020 | Europe / Portugal |  | 2020-03-23 | EPI_ISL_421495 |
| Portugal/PT0110/2020 | Europe / Portugal |  | 2020-03-23 | EPI_ISL_453826 |
| Portugal/PT0124/2020 | Europe / Portugal |  | 2020-03-23 | EPI_ISL_453840 |
| Portugal/PT0128/2020 | Europe / Portugal |  | 2020-03-23 | EPI_ISL_453844 |
| Portugal/PT0129/2020 | Europe / Portugal |  | 2020-03-23 | EPI_ISL_453845 |
| Portugal/PT0130/2020 | Europe / Portugal |  | 2020-03-23 | EPI_ISL_453846 |
| Portugal/PT0413/2020 | Europe / Portugal |  | 2020-03-23 | EPI_ISL_454137 |
| Portugal/PT0414/2020 | Europe / Portugal |  | 2020-03-23 | EPI_ISL_454138 |
| Portugal/PT0415/2020 | Europe / Portugal |  | 2020-03-23 | EPI_ISL_454139 |
| Portugal/PT0416/2020 | Europe / Portugal |  | 2020-03-23 | EPI_ISL_454140 |
| Portugal/PT0440/2020 | Europe / Portugal |  | 2020-03-23 | EPI_ISL_454164 |
| Portugal/PT0441/2020 | Europe / Portugal |  | 2020-03-23 | EPI_ISL_454165 |
| Portugal/PT0442/2020 | Europe / Portugal |  | 2020-03-23 | EPI_ISL_454166 |
| Portugal/PT0443/2020 | Europe / Portugal |  | 2020-03-23 | EPI_ISL_454167 |
| Portugal/PT0521/2020 | Europe / Portugal |  | 2020-03-23 | EPI_ISL_454245 |
| Portugal/PT0522/2020 | Europe / Portugal |  | 2020-03-23 | EPI_ISL_454246 |
| Portugal/PT0523/2020 | Europe / Portugal |  | 2020-03-23 | EPI_ISL_454247 |
| Portugal/PT0524/2020 | Europe / Portugal |  | 2020-03-23 | EPI_ISL_454248 |
| Portugal/PT0525/2020 | Europe / Portugal |  | 2020-03-23 | EPI_ISL_454249 |
| Portugal/PT0664/2020 | Europe / Portugal |  | 2020-03-23 | EPI_ISL_510923 |
| Portugal/PT0708/2020 | Europe / Portugal |  | 2020-03-23 | EPI_ISL_510967 |
| Portugal/PT0709/2020 | Europe / Portugal |  | 2020-03-23 | EPI_ISL_510968 |
| Portugal/PT0710/2020 | Europe / Portugal |  | 2020-03-23 | EPI_ISL_510969 |
| Portugal/PT0750/2020 | Europe / Portugal |  | 2020-03-23 | EPI_ISL_511009 |
| Portugal/PT0751/2020 | Europe / Portugal |  | 2020-03-23 | EPI_ISL_511010 |
| Portugal/PT0752/2020 | Europe / Portugal |  | 2020-03-23 | EPI_ISL_511011 |
| Portugal/PT0753/2020 | Europe / Portugal |  | 2020-03-23 | EPI_ISL_511012 |
| Portugal/PT0754/2020 | Europe / Portugal |  | 2020-03-23 | EPI_ISL_511013 |
| Portugal/PT0755/2020 | Europe / Portugal |  | 2020-03-23 | EPI_ISL_511014 |
| Portugal/PT0756/2020 | Europe / Portugal |  | 2020-03-23 | EPI_ISL_511015 |
| Portugal/PT0757/2020 | Europe / Portugal |  | 2020-03-23 | EPI_ISL_511016 |
| Portugal/PT0929/2020 | Europe / Portugal |  | 2020-03-23 | EPI_ISL_511186 |
| Portugal/PT0986/2020 | Europe / Portugal |  | 2020-03-23 | EPI_ISL_511534 |
| Portugal/PT0987/2020 | Europe / Portugal |  | 2020-03-23 | EPI_ISL_511535 |
| Portugal/PT0988/2020 | Europe / Portugal |  | 2020-03-23 | EPI_ISL_511536 |
| Portugal/PT0989/2020 | Europe / Portugal |  | 2020-03-23 | EPI_ISL_511537 |
| Portugal/PT0990/2020 | Europe / Portugal |  | 2020-03-23 | EPI_ISL_511538 |
| Portugal/PT0991/2020 | Europe / Portugal |  | 2020-03-23 | EPI_ISL_511539 |
| Portugal/PT1022/2020 | Europe / Portugal | yes | 2020-03-23 | EPI_ISL_511569 |
| Portugal/PT0659a/2020 | Europe / Portugal | yes | 2020-03-23 | EPI_ISL_511483 |
| Portugal/PT1024/2020 | Europe / Portugal |  | 2020-03-23 | EPI_ISL_511571 |
| Portugal/PT0657a/2020 | Europe / Portugal | yes | 2020-03-23 | EPI_ISL_511482 |
| Portugal/PT1175/2020 | Europe / Portugal |  | 2020-03-23 | EPI_ISL_511721 |
| Portugal/PT1176/2020 | Europe / Portugal |  | 2020-03-23 | EPI_ISL_511722 |
| Portugal/PT1277/2020 | Europe / Portugal |  | 2020-03-23 | EPI_ISL_511238 |
| Portugal/PT1278/2020 | Europe / Portugal |  | 2020-03-23 | EPI_ISL_511239 |
| Portugal/PT1279/2020 | Europe / Portugal |  | 2020-03-23 | EPI_ISL_511240 |
| Portugal/PT1280/2020 | Europe / Portugal |  | 2020-03-23 | EPI_ISL_511241 |
| Portugal/PT1281/2020 | Europe / Portugal |  | 2020-03-23 | EPI_ISL_511242 |
| Portugal/PT1282/2020 | Europe / Portugal |  | 2020-03-23 | EPI_ISL_511243 |
| Portugal/PT1408/2020 | Europe / Portugal |  | 2020-03-23 | EPI_ISL_511367 |
| Portugal/PT0093/2020 | Europe / Portugal |  | 2020-03-24 | EPI_ISL_421496 |
| Portugal/PT0094/2020 | Europe / Portugal |  | 2020-03-24 | EPI_ISL_421497 |
| Portugal/PT0095/2020 | Europe / Portugal |  | 2020-03-24 | EPI_ISL_421498 |
| Portugal/PT0096/2020 | Europe / Portugal |  | 2020-03-24 | EPI_ISL_421499 |
| Portugal/PT0097/2020 | Europe / Portugal |  | 2020-03-24 | EPI_ISL_453813 |
| Portugal/PT0098/2020 | Europe / Portugal |  | 2020-03-24 | EPI_ISL_453814 |
| Portugal/PT0099/2020 | Europe / Portugal |  | 2020-03-24 | EPI_ISL_453815 |
| Portugal/PT0100/2020 | Europe / Portugal |  | 2020-03-24 | EPI_ISL_453816 |
| Portugal/PT0101/2020 | Europe / Portugal |  | 2020-03-24 | EPI_ISL_453817 |
| Portugal/PT0102/2020 | Europe / Portugal |  | 2020-03-24 | EPI_ISL_453818 |
| Portugal/PT0127/2020 | Europe / Portugal |  | 2020-03-24 | EPI_ISL_453843 |
| Portugal/PT0149/2020 | Europe / Portugal |  | 2020-03-24 | EPI_ISL_453865 |
| Portugal/PT0417/2020 | Europe / Portugal |  | 2020-03-24 | EPI_ISL_454141 |
| Portugal/PT0418/2020 | Europe / Portugal |  | 2020-03-24 | EPI_ISL_454142 |
| Portugal/PT0577/2020 | Europe / Portugal |  | 2020-03-24 | EPI_ISL_454300 |
| Portugal/PT0578/2020 | Europe / Portugal |  | 2020-03-24 | EPI_ISL_454301 |
| Portugal/PT0579/2020 | Europe / Portugal |  | 2020-03-24 | EPI_ISL_454302 |
| Portugal/PT0620/2020 | Europe / Portugal | yes | 2020-03-24 | EPI_ISL_454343 |
| Portugal/PT0630/2020 | Europe / Portugal | yes | 2020-03-24 | EPI_ISL_510891 |
| Portugal/PT0631/2020 | Europe / Portugal | yes | 2020-03-24 | EPI_ISL_510892 |
| Portugal/PT0632/2020 | Europe / Portugal |  | 2020-03-24 | EPI_ISL_510893 |
| Portugal/PT0665/2020 | Europe / Portugal |  | 2020-03-24 | EPI_ISL_510924 |
| Portugal/PT0669/2020 | Europe / Portugal |  | 2020-03-24 | EPI_ISL_510928 |
| Portugal/PT0676/2020 | Europe / Portugal |  | 2020-03-24 | EPI_ISL_510935 |
| Portugal/PT0677/2020 | Europe / Portugal |  | 2020-03-24 | EPI_ISL_510936 |
| Portugal/PT0711/2020 | Europe / Portugal | yes | 2020-03-24 | EPI_ISL_510970 |
| Portugal/PT0712/2020 | Europe / Portugal |  | 2020-03-24 | EPI_ISL_510971 |
| Portugal/PT0713/2020 | Europe / Portugal |  | 2020-03-24 | EPI_ISL_510972 |
| Portugal/PT0714/2020 | Europe / Portugal |  | 2020-03-24 | EPI_ISL_510973 |
| Portugal/PT0715/2020 | Europe / Portugal |  | 2020-03-24 | EPI_ISL_510974 |
| Portugal/PT0716/2020 | Europe / Portugal |  | 2020-03-24 | EPI_ISL_510975 |
| Portugal/PT0758a/2020 | Europe / Portugal |  | 2020-03-24 | EPI_ISL_511017 |
| Portugal/PT0759/2020 | Europe / Portugal |  | 2020-03-24 | EPI_ISL_511018 |
| Portugal/PT0760/2020 | Europe / Portugal |  | 2020-03-24 | EPI_ISL_511019 |
| Portugal/PT0761/2020 | Europe / Portugal |  | 2020-03-24 | EPI_ISL_511020 |
| Portugal/PT0762/2020 | Europe / Portugal |  | 2020-03-24 | EPI_ISL_511021 |
| Portugal/PT0763/2020 | Europe / Portugal |  | 2020-03-24 | EPI_ISL_511022 |
| Portugal/PT0961/2020 | Europe / Portugal |  | 2020-03-24 | EPI_ISL_511510 |
| Portugal/PT0962/2020 | Europe / Portugal |  | 2020-03-24 | EPI_ISL_511511 |
| Portugal/PT1002/2020 | Europe / Portugal |  | 2020-03-24 | EPI_ISL_511550 |
| Portugal/PT1003/2020 | Europe / Portugal |  | 2020-03-24 | EPI_ISL_582517 |
| Portugal/PT1023/2020 | Europe / Portugal |  | 2020-03-24 | EPI_ISL_511570 |
| Portugal/PT1026/2020 | Europe / Portugal |  | 2020-03-24 | EPI_ISL_511573 |
| Portugal/PT1027/2020 | Europe / Portugal |  | 2020-03-24 | EPI_ISL_511574 |
| Portugal/PT1028/2020 | Europe / Portugal |  | 2020-03-24 | EPI_ISL_511575 |
| Portugal/PT1177/2020 | Europe / Portugal |  | 2020-03-24 | EPI_ISL_511723 |
| Portugal/PT1178/2020 | Europe / Portugal |  | 2020-03-24 | EPI_ISL_511724 |
| Portugal/PT1179/2020 | Europe / Portugal |  | 2020-03-24 | EPI_ISL_582519 |
| Portugal/PT1180/2020 | Europe / Portugal |  | 2020-03-24 | EPI_ISL_511725 |
| Portugal/PT1219/2020 | Europe / Portugal |  | 2020-03-24 | EPI_ISL_511762 |
| Portugal/PT1283/2020 | Europe / Portugal |  | 2020-03-24 | EPI_ISL_511244 |
| Portugal/PT1284/2020 | Europe / Portugal |  | 2020-03-24 | EPI_ISL_511245 |
| Portugal/PT1285/2020 | Europe / Portugal |  | 2020-03-24 | EPI_ISL_511246 |
| Portugal/PT1286/2020 | Europe / Portugal |  | 2020-03-24 | EPI_ISL_511247 |
| Portugal/PT1287/2020 | Europe / Portugal |  | 2020-03-24 | EPI_ISL_511248 |
| Portugal/PT1288/2020 | Europe / Portugal |  | 2020-03-24 | EPI_ISL_511249 |
| Portugal/PT1289/2020 | Europe / Portugal |  | 2020-03-24 | EPI_ISL_511250 |
| Portugal/PT1290/2020 | Europe / Portugal |  | 2020-03-24 | EPI_ISL_511251 |
| Portugal/PT1291/2020 | Europe / Portugal |  | 2020-03-24 | EPI_ISL_511252 |
| Portugal/PT1292/2020 | Europe / Portugal |  | 2020-03-24 | EPI_ISL_511253 |
| Portugal/PT1293/2020 | Europe / Portugal |  | 2020-03-24 | EPI_ISL_511254 |
| Portugal/PT1294/2020 | Europe / Portugal |  | 2020-03-24 | EPI_ISL_511255 |
| Portugal/PT1295/2020 | Europe / Portugal |  | 2020-03-24 | EPI_ISL_582522 |
| Portugal/PT1296/2020 | Europe / Portugal |  | 2020-03-24 | EPI_ISL_511256 |
| Portugal/PT1297/2020 | Europe / Portugal |  | 2020-03-24 | EPI_ISL_582523 |
| Portugal/PT1347/2020 | Europe / Portugal |  | 2020-03-24 | EPI_ISL_511306 |
| Portugal/PT1359/2020 | Europe / Portugal |  | 2020-03-24 | EPI_ISL_511318 |
| Portugal/PT1360/2020 | Europe / Portugal |  | 2020-03-24 | EPI_ISL_511319 |
| Portugal/PT1361/2020 | Europe / Portugal |  | 2020-03-24 | EPI_ISL_511320 |
| Portugal/PT1362/2020 | Europe / Portugal |  | 2020-03-24 | EPI_ISL_511321 |
| Portugal/PT1363/2020 | Europe / Portugal |  | 2020-03-24 | EPI_ISL_511322 |
| Portugal/PT1364/2020 | Europe / Portugal |  | 2020-03-24 | EPI_ISL_511323 |
| Portugal/PT1365/2020 | Europe / Portugal | yes | 2020-03-24 | EPI_ISL_511324 |
| Portugal/PT1366/2020 | Europe / Portugal |  | 2020-03-24 | EPI_ISL_511325 |
| Portugal/PT1367/2020 | Europe / Portugal | yes | 2020-03-24 | EPI_ISL_511326 |
| Portugal/PT1368/2020 | Europe / Portugal |  | 2020-03-24 | EPI_ISL_511327 |
| Portugal/PT1369/2020 | Europe / Portugal |  | 2020-03-24 | EPI_ISL_511328 |
| Portugal/PT1404/2020 | Europe / Portugal |  | 2020-03-24 | EPI_ISL_511363 |
| Portugal/PT1409/2020 | Europe / Portugal |  | 2020-03-24 | EPI_ISL_511368 |
| Portugal/PT1431/2020 | Europe / Portugal |  | 2020-03-24 | EPI_ISL_511390 |
| Portugal/PT1433/2020 | Europe / Portugal |  | 2020-03-24 | EPI_ISL_511392 |
| Portugal/PT1434/2020 | Europe / Portugal |  | 2020-03-24 | EPI_ISL_511393 |
| Portugal/PT1441/2020 | Europe / Portugal |  | 2020-03-24 | EPI_ISL_511400 |
| Portugal/PT1443/2020 | Europe / Portugal |  | 2020-03-24 | EPI_ISL_511402 |
| Portugal/PT1466/2020 | Europe / Portugal |  | 2020-03-24 | EPI_ISL_511425 |
| Portugal/PT0111/2020 | Europe / Portugal |  | 2020-03-25 | EPI_ISL_453827 |
| Portugal/PT0112/2020 | Europe / Portugal |  | 2020-03-25 | EPI_ISL_453828 |
| Portugal/PT0113/2020 | Europe / Portugal |  | 2020-03-25 | EPI_ISL_453829 |
| Portugal/PT0114/2020 | Europe / Portugal |  | 2020-03-25 | EPI_ISL_453830 |
| Portugal/PT0115/2020 | Europe / Portugal |  | 2020-03-25 | EPI_ISL_453831 |
| Portugal/PT0116/2020 | Europe / Portugal |  | 2020-03-25 | EPI_ISL_453832 |
| Portugal/PT0117/2020 | Europe / Portugal |  | 2020-03-25 | EPI_ISL_453833 |
| Portugal/PT0118/2020 | Europe / Portugal |  | 2020-03-25 | EPI_ISL_453834 |
| Portugal/PT0119/2020 | Europe / Portugal |  | 2020-03-25 | EPI_ISL_453835 |
| Portugal/PT0120/2020 | Europe / Portugal |  | 2020-03-25 | EPI_ISL_453836 |
| Portugal/PT0123/2020 | Europe / Portugal |  | 2020-03-25 | EPI_ISL_453839 |
| Portugal/PT0125/2020 | Europe / Portugal |  | 2020-03-25 | EPI_ISL_453841 |
| Portugal/PT0131/2020 | Europe / Portugal |  | 2020-03-25 | EPI_ISL_453847 |
| Portugal/PT0132/2020 | Europe / Portugal |  | 2020-03-25 | EPI_ISL_453848 |
| Portugal/PT0151/2020 | Europe / Portugal |  | 2020-03-25 | EPI_ISL_453867 |
| Portugal/PT0419/2020 | Europe / Portugal |  | 2020-03-25 | EPI_ISL_454143 |
| Portugal/PT0420/2020 | Europe / Portugal |  | 2020-03-25 | EPI_ISL_454144 |
| Portugal/PT0478/2020 | Europe / Portugal |  | 2020-03-25 | EPI_ISL_454202 |
| Portugal/PT0479/2020 | Europe / Portugal |  | 2020-03-25 | EPI_ISL_454203 |
| Portugal/PT0580/2020 | Europe / Portugal |  | 2020-03-25 | EPI_ISL_454303 |
| Portugal/PT0621/2020 | Europe / Portugal |  | 2020-03-25 | EPI_ISL_454344 |
| Portugal/PT0633/2020 | Europe / Portugal | yes | 2020-03-25 | EPI_ISL_510894 |
| Portugal/PT0634/2020 | Europe / Portugal | yes | 2020-03-25 | EPI_ISL_510895 |
| Portugal/PT0635/2020 | Europe / Portugal |  | 2020-03-25 | EPI_ISL_510896 |
| Portugal/PT0652/2020 | Europe / Portugal |  | 2020-03-25 | EPI_ISL_510913 |
| Portugal/PT0653/2020 | Europe / Portugal |  | 2020-03-25 | EPI_ISL_510914 |
| Portugal/PT0673/2020 | Europe / Portugal |  | 2020-03-25 | EPI_ISL_510932 |
| Portugal/PT0678/2020 | Europe / Portugal |  | 2020-03-25 | EPI_ISL_510937 |
| Portugal/PT0679/2020 | Europe / Portugal |  | 2020-03-25 | EPI_ISL_510938 |
| Portugal/PT0680/2020 | Europe / Portugal |  | 2020-03-25 | EPI_ISL_510939 |
| Portugal/PT0682/2020 | Europe / Portugal |  | 2020-03-25 | EPI_ISL_510941 |
| Portugal/PT0717/2020 | Europe / Portugal |  | 2020-03-25 | EPI_ISL_510976 |
| Portugal/PT0718/2020 | Europe / Portugal |  | 2020-03-25 | EPI_ISL_510977 |
| Portugal/PT0719/2020 | Europe / Portugal |  | 2020-03-25 | EPI_ISL_510978 |
| Portugal/PT0764/2020 | Europe / Portugal |  | 2020-03-25 | EPI_ISL_511023 |
| Portugal/PT0765/2020 | Europe / Portugal |  | 2020-03-25 | EPI_ISL_511024 |
| Portugal/PT0766/2020 | Europe / Portugal | yes | 2020-03-25 | EPI_ISL_511025 |
| Portugal/PT0930/2020 | Europe / Portugal |  | 2020-03-25 | EPI_ISL_511187 |
| Portugal/PT0931/2020 | Europe / Portugal |  | 2020-03-25 | EPI_ISL_511188 |
| Portugal/PT0964/2020 | Europe / Portugal |  | 2020-03-25 | EPI_ISL_511513 |
| Portugal/PT0965/2020 | Europe / Portugal |  | 2020-03-25 | EPI_ISL_511514 |
| Portugal/PT0966/2020 | Europe / Portugal | yes | 2020-03-25 | EPI_ISL_511515 |
| Portugal/PT1004/2020 | Europe / Portugal |  | 2020-03-25 | EPI_ISL_511551 |
| Portugal/PT1017/2020 | Europe / Portugal | yes | 2020-03-25 | EPI_ISL_511564 |
| Portugal/PT1124/2020 | Europe / Portugal |  | 2020-03-25 | EPI_ISL_511671 |
| Portugal/PT1181/2020 | Europe / Portugal |  | 2020-03-25 | EPI_ISL_511726 |
| Portugal/PT1182/2020 | Europe / Portugal |  | 2020-03-25 | EPI_ISL_511727 |
| Portugal/PT1183/2020 | Europe / Portugal |  | 2020-03-25 | EPI_ISL_511728 |
| Portugal/PT1184/2020 | Europe / Portugal |  | 2020-03-25 | EPI_ISL_511729 |
| Portugal/PT1185/2020 | Europe / Portugal |  | 2020-03-25 | EPI_ISL_511730 |
| Portugal/PT1186/2020 | Europe / Portugal |  | 2020-03-25 | EPI_ISL_511731 |
| Portugal/PT1227/2020 | Europe / Portugal |  | 2020-03-25 | EPI_ISL_511770 |
| Portugal/PT1346/2020 | Europe / Portugal | yes | 2020-03-25 | EPI_ISL_511305 |
| Portugal/PT1349/2020 | Europe / Portugal | yes | 2020-03-25 | EPI_ISL_511308 |
| Portugal/PT1350/2020 | Europe / Portugal |  | 2020-03-25 | EPI_ISL_511309 |
| Portugal/PT1351/2020 | Europe / Portugal |  | 2020-03-25 | EPI_ISL_511310 |
| Portugal/PT1352/2020 | Europe / Portugal |  | 2020-03-25 | EPI_ISL_511311 |
| Portugal/PT1353/2020 | Europe / Portugal | yes | 2020-03-25 | EPI_ISL_511312 |
| Portugal/PT1354/2020 | Europe / Portugal | yes | 2020-03-25 | EPI_ISL_511313 |
| Portugal/PT1355/2020 | Europe / Portugal |  | 2020-03-25 | EPI_ISL_511314 |
| Portugal/PT1356/2020 | Europe / Portugal | yes | 2020-03-25 | EPI_ISL_511315 |
| Portugal/PT1357/2020 | Europe / Portugal |  | 2020-03-25 | EPI_ISL_511316 |
| Portugal/PT1358/2020 | Europe / Portugal |  | 2020-03-25 | EPI_ISL_511317 |
| Portugal/PT1371/2020 | Europe / Portugal |  | 2020-03-25 | EPI_ISL_511330 |
| Portugal/PT1372/2020 | Europe / Portugal |  | 2020-03-25 | EPI_ISL_511331 |
| Portugal/PT1373/2020 | Europe / Portugal |  | 2020-03-25 | EPI_ISL_511332 |
| Portugal/PT1374/2020 | Europe / Portugal |  | 2020-03-25 | EPI_ISL_511333 |
| Portugal/PT1375/2020 | Europe / Portugal |  | 2020-03-25 | EPI_ISL_511334 |
| Portugal/PT1407/2020 | Europe / Portugal |  | 2020-03-25 | EPI_ISL_511366 |
| Portugal/PT1410/2020 | Europe / Portugal |  | 2020-03-25 | EPI_ISL_511369 |
| Portugal/PT1412/2020 | Europe / Portugal | yes | 2020-03-25 | EPI_ISL_511371 |
| Portugal/PT1419/2020 | Europe / Portugal |  | 2020-03-25 | EPI_ISL_511378 |
| Portugal/PT1427/2020 | Europe / Portugal |  | 2020-03-25 | EPI_ISL_511386 |
| Portugal/PT1442/2020 | Europe / Portugal |  | 2020-03-25 | EPI_ISL_511401 |
| Portugal/PT1444/2020 | Europe / Portugal |  | 2020-03-25 | EPI_ISL_511403 |
| Portugal/PT0121/2020 | Europe / Portugal |  | 2020-03-26 | EPI_ISL_453837 |
| Portugal/PT0122/2020 | Europe / Portugal |  | 2020-03-26 | EPI_ISL_453838 |
| Portugal/PT0126/2020 | Europe / Portugal |  | 2020-03-26 | EPI_ISL_453842 |
| Portugal/PT0133/2020 | Europe / Portugal |  | 2020-03-26 | EPI_ISL_453849 |
| Portugal/PT0134/2020 | Europe / Portugal |  | 2020-03-26 | EPI_ISL_453850 |
| Portugal/PT0135/2020 | Europe / Portugal |  | 2020-03-26 | EPI_ISL_453851 |
| Portugal/PT0136/2020 | Europe / Portugal |  | 2020-03-26 | EPI_ISL_453852 |
| Portugal/PT0137/2020 | Europe / Portugal |  | 2020-03-26 | EPI_ISL_453853 |
| Portugal/PT0216/2020 | Europe / Portugal |  | 2020-03-26 | EPI_ISL_453930 |
| Portugal/PT0444/2020 | Europe / Portugal |  | 2020-03-26 | EPI_ISL_454168 |
| Portugal/PT0445/2020 | Europe / Portugal |  | 2020-03-26 | EPI_ISL_454169 |
| Portugal/PT0564/2020 | Europe / Portugal |  | 2020-03-26 | EPI_ISL_454287 |
| Portugal/PT0565/2020 | Europe / Portugal |  | 2020-03-26 | EPI_ISL_454288 |
| Portugal/PT0571/2020 | Europe / Portugal |  | 2020-03-26 | EPI_ISL_454294 |
| Portugal/PT0572/2020 | Europe / Portugal |  | 2020-03-26 | EPI_ISL_454295 |
| Portugal/PT0582/2020 | Europe / Portugal |  | 2020-03-26 | EPI_ISL_454305 |
| Portugal/PT0603/2020 | Europe / Portugal |  | 2020-03-26 | EPI_ISL_454326 |
| Portugal/PT0622/2020 | Europe / Portugal |  | 2020-03-26 | EPI_ISL_454345 |
| Portugal/PT0623/2020 | Europe / Portugal | yes | 2020-03-26 | EPI_ISL_454346 |
| Portugal/PT0624/2020 | Europe / Portugal |  | 2020-03-26 | EPI_ISL_454347 |
| Portugal/PT0654/2020 | Europe / Portugal | yes | 2020-03-26 | EPI_ISL_510915 |
| Portugal/PT0666/2020 | Europe / Portugal |  | 2020-03-26 | EPI_ISL_510925 |
| Portugal/PT0720/2020 | Europe / Portugal |  | 2020-03-26 | EPI_ISL_510979 |
| Portugal/PT0721/2020 | Europe / Portugal |  | 2020-03-26 | EPI_ISL_510980 |
| Portugal/PT0768/2020 | Europe / Portugal | yes | 2020-03-26 | EPI_ISL_511027 |
| Portugal/PT0897/2020 | Europe / Portugal |  | 2020-03-26 | EPI_ISL_511154 |
| Portugal/PT0932/2020 | Europe / Portugal |  | 2020-03-26 | EPI_ISL_511484 |
| Portugal/PT0960/2020 | Europe / Portugal |  | 2020-03-26 | EPI_ISL_511509 |
| Portugal/PT0963/2020 | Europe / Portugal |  | 2020-03-26 | EPI_ISL_511512 |
| Portugal/PT1005/2020 | Europe / Portugal | yes | 2020-03-26 | EPI_ISL_511552 |
| Portugal/PT1012/2020 | Europe / Portugal |  | 2020-03-26 | EPI_ISL_511559 |
| Portugal/PT1013/2020 | Europe / Portugal | yes | 2020-03-26 | EPI_ISL_511560 |
| Portugal/PT1014/2020 | Europe / Portugal | yes | 2020-03-26 | EPI_ISL_511561 |
| Portugal/PT1015/2020 | Europe / Portugal | yes | 2020-03-26 | EPI_ISL_511562 |
| Portugal/PT1016/2020 | Europe / Portugal |  | 2020-03-26 | EPI_ISL_511563 |
| Portugal/PT1018/2020 | Europe / Portugal |  | 2020-03-26 | EPI_ISL_511565 |
| Portugal/PT1019/2020 | Europe / Portugal |  | 2020-03-26 | EPI_ISL_511566 |
| Portugal/PT1020/2020 | Europe / Portugal | yes | 2020-03-26 | EPI_ISL_511567 |
| Portugal/PT1021/2020 | Europe / Portugal |  | 2020-03-26 | EPI_ISL_511568 |
| Portugal/PT1025/2020 | Europe / Portugal |  | 2020-03-26 | EPI_ISL_511572 |
| Portugal/PT1029/2020 | Europe / Portugal |  | 2020-03-26 | EPI_ISL_511576 |
| Portugal/PT1034/2020 | Europe / Portugal |  | 2020-03-26 | EPI_ISL_511581 |
| Portugal/PT1035/2020 | Europe / Portugal |  | 2020-03-26 | EPI_ISL_511582 |
| Portugal/PT1036/2020 | Europe / Portugal |  | 2020-03-26 | EPI_ISL_511583 |
| Portugal/PT1037/2020 | Europe / Portugal |  | 2020-03-26 | EPI_ISL_511584 |
| Portugal/PT1125/2020 | Europe / Portugal |  | 2020-03-26 | EPI_ISL_511672 |
| Portugal/PT1126/2020 | Europe / Portugal |  | 2020-03-26 | EPI_ISL_511673 |
| Portugal/PT1187/2020 | Europe / Portugal |  | 2020-03-26 | EPI_ISL_511732 |
| Portugal/PT1188/2020 | Europe / Portugal |  | 2020-03-26 | EPI_ISL_511733 |
| Portugal/PT1208/2020 | Europe / Portugal |  | 2020-03-26 | EPI_ISL_511751 |
| Portugal/PT1209/2020 | Europe / Portugal |  | 2020-03-26 | EPI_ISL_511752 |
| Portugal/PT1210/2020 | Europe / Portugal | yes | 2020-03-26 | EPI_ISL_511753 |
| Portugal/PT1211/2020 | Europe / Portugal | yes | 2020-03-26 | EPI_ISL_511754 |
| Portugal/PT1212/2020 | Europe / Portugal | yes | 2020-03-26 | EPI_ISL_511755 |
| Portugal/PT1213/2020 | Europe / Portugal | yes | 2020-03-26 | EPI_ISL_511756 |
| Portugal/PT1214/2020 | Europe / Portugal | yes | 2020-03-26 | EPI_ISL_511757 |
| Portugal/PT1215/2020 | Europe / Portugal | yes | 2020-03-26 | EPI_ISL_511758 |
| Portugal/PT1376/2020 | Europe / Portugal |  | 2020-03-26 | EPI_ISL_511335 |
| Portugal/PT1377/2020 | Europe / Portugal |  | 2020-03-26 | EPI_ISL_511336 |
| Portugal/PT1378/2020 | Europe / Portugal |  | 2020-03-26 | EPI_ISL_511337 |
| Portugal/PT1379/2020 | Europe / Portugal |  | 2020-03-26 | EPI_ISL_511338 |
| Portugal/PT1380/2020 | Europe / Portugal |  | 2020-03-26 | EPI_ISL_511339 |
| Portugal/PT1405/2020 | Europe / Portugal | yes | 2020-03-26 | EPI_ISL_511364 |
| Portugal/PT1414/2020 | Europe / Portugal |  | 2020-03-26 | EPI_ISL_511373 |
| Portugal/PT1418/2020 | Europe / Portugal |  | 2020-03-26 | EPI_ISL_511377 |
| Portugal/PT1420/2020 | Europe / Portugal |  | 2020-03-26 | EPI_ISL_511379 |
| Portugal/PT1421/2020 | Europe / Portugal |  | 2020-03-26 | EPI_ISL_511380 |
| Portugal/PT1422/2020 | Europe / Portugal |  | 2020-03-26 | EPI_ISL_511381 |
| Portugal/PT1432/2020 | Europe / Portugal |  | 2020-03-26 | EPI_ISL_511391 |
| Portugal/PT1435/2020 | Europe / Portugal |  | 2020-03-26 | EPI_ISL_511394 |
| Portugal/PT1437/2020 | Europe / Portugal |  | 2020-03-26 | EPI_ISL_511396 |
| Portugal/PT1446/2020 | Europe / Portugal |  | 2020-03-26 | EPI_ISL_511405 |
| Portugal/PT1449/2020 | Europe / Portugal | yes | 2020-03-26 | EPI_ISL_511408 |
| Portugal/PT1451/2020 | Europe / Portugal |  | 2020-03-26 | EPI_ISL_511410 |
| Portugal/PT1452/2020 | Europe / Portugal |  | 2020-03-26 | EPI_ISL_511411 |
| Portugal/PT1454/2020 | Europe / Portugal |  | 2020-03-26 | EPI_ISL_511413 |
| Portugal/PT1457/2020 | Europe / Portugal |  | 2020-03-26 | EPI_ISL_511416 |
| Portugal/PT1459/2020 | Europe / Portugal | yes | 2020-03-26 | EPI_ISL_511418 |
| Portugal/PT1471/2020 | Europe / Portugal | yes | 2020-03-26 | EPI_ISL_511430 |
| Portugal/PT1484/2020 | Europe / Portugal | yes | 2020-03-26 | EPI_ISL_511443 |
| Portugal/PT0138/2020 | Europe / Portugal |  | 2020-03-27 | EPI_ISL_453854 |
| Portugal/PT0139/2020 | Europe / Portugal |  | 2020-03-27 | EPI_ISL_453855 |
| Portugal/PT0140/2020 | Europe / Portugal |  | 2020-03-27 | EPI_ISL_453856 |
| Portugal/PT0141/2020 | Europe / Portugal |  | 2020-03-27 | EPI_ISL_453857 |
| Portugal/PT0142/2020 | Europe / Portugal |  | 2020-03-27 | EPI_ISL_453858 |
| Portugal/PT0143/2020 | Europe / Portugal |  | 2020-03-27 | EPI_ISL_453859 |
| Portugal/PT0144/2020 | Europe / Portugal |  | 2020-03-27 | EPI_ISL_453860 |
| Portugal/PT0145/2020 | Europe / Portugal |  | 2020-03-27 | EPI_ISL_453861 |
| Portugal/PT0146/2020 | Europe / Portugal |  | 2020-03-27 | EPI_ISL_453862 |
| Portugal/PT0147/2020 | Europe / Portugal |  | 2020-03-27 | EPI_ISL_453863 |
| Portugal/PT0154/2020 | Europe / Portugal |  | 2020-03-27 | EPI_ISL_453870 |
| Portugal/PT0155/2020 | Europe / Portugal |  | 2020-03-27 | EPI_ISL_453871 |
| Portugal/PT0156/2020 | Europe / Portugal | yes | 2020-03-27 | EPI_ISL_453872 |
| Portugal/PT0157/2020 | Europe / Portugal |  | 2020-03-27 | EPI_ISL_453873 |
| Portugal/PT0158/2020 | Europe / Portugal |  | 2020-03-27 | EPI_ISL_453874 |
| Portugal/PT0159/2020 | Europe / Portugal | yes | 2020-03-27 | EPI_ISL_453875 |
| Portugal/PT0160/2020 | Europe / Portugal | yes | 2020-03-27 | EPI_ISL_453876 |
| Portugal/PT0204/2020 | Europe / Portugal |  | 2020-03-27 | EPI_ISL_453918 |
| Portugal/PT0205/2020 | Europe / Portugal |  | 2020-03-27 | EPI_ISL_453919 |
| Portugal/PT0446/2020 | Europe / Portugal |  | 2020-03-27 | EPI_ISL_454170 |
| Portugal/PT0447/2020 | Europe / Portugal |  | 2020-03-27 | EPI_ISL_454171 |
| Portugal/PT0480/2020 | Europe / Portugal |  | 2020-03-27 | EPI_ISL_454204 |
| Portugal/PT0583/2020 | Europe / Portugal |  | 2020-03-27 | EPI_ISL_454306 |
| Portugal/PT0584/2020 | Europe / Portugal |  | 2020-03-27 | EPI_ISL_454307 |
| Portugal/PT0586/2020 | Europe / Portugal |  | 2020-03-27 | EPI_ISL_454309 |
| Portugal/PT0587/2020 | Europe / Portugal |  | 2020-03-27 | EPI_ISL_454310 |
| Portugal/PT0604/2020 | Europe / Portugal | yes | 2020-03-27 | EPI_ISL_454327 |
| Portugal/PT0625/2020 | Europe / Portugal |  | 2020-03-27 | EPI_ISL_454348 |
| Portugal/PT0667/2020 | Europe / Portugal |  | 2020-03-27 | EPI_ISL_510926 |
| Portugal/PT0683/2020 | Europe / Portugal |  | 2020-03-27 | EPI_ISL_510942 |
| Portugal/PT0684/2020 | Europe / Portugal |  | 2020-03-27 | EPI_ISL_510943 |
| Portugal/PT0722/2020 | Europe / Portugal |  | 2020-03-27 | EPI_ISL_510981 |
| Portugal/PT0723/2020 | Europe / Portugal |  | 2020-03-27 | EPI_ISL_510982 |
| Portugal/PT0724/2020 | Europe / Portugal |  | 2020-03-27 | EPI_ISL_510983 |
| Portugal/PT0725/2020 | Europe / Portugal |  | 2020-03-27 | EPI_ISL_510984 |
| Portugal/PT0726/2020 | Europe / Portugal |  | 2020-03-27 | EPI_ISL_510985 |
| Portugal/PT0727/2020 | Europe / Portugal |  | 2020-03-27 | EPI_ISL_510986 |
| Portugal/PT0728/2020 | Europe / Portugal |  | 2020-03-27 | EPI_ISL_510987 |
| Portugal/PT0767/2020 | Europe / Portugal | yes | 2020-03-27 | EPI_ISL_511026 |
| Portugal/PT0848/2020 | Europe / Portugal |  | 2020-03-27 | EPI_ISL_511107 |
| Portugal/PT0849/2020 | Europe / Portugal | yes | 2020-03-27 | EPI_ISL_511108 |
| Portugal/PT0850/2020 | Europe / Portugal | yes | 2020-03-27 | EPI_ISL_511109 |
| Portugal/PT0851/2020 | Europe / Portugal | yes | 2020-03-27 | EPI_ISL_511110 |
| Portugal/PT0852/2020 | Europe / Portugal | yes | 2020-03-27 | EPI_ISL_511111 |
| Portugal/PT0853/2020 | Europe / Portugal | yes | 2020-03-27 | EPI_ISL_511112 |
| Portugal/PT0854/2020 | Europe / Portugal | yes | 2020-03-27 | EPI_ISL_511113 |
| Portugal/PT0855/2020 | Europe / Portugal | yes | 2020-03-27 | EPI_ISL_511114 |
| Portugal/PT0856/2020 | Europe / Portugal |  | 2020-03-27 | EPI_ISL_511115 |
| Portugal/PT0857/2020 | Europe / Portugal | yes | 2020-03-27 | EPI_ISL_511116 |
| Portugal/PT0858/2020 | Europe / Portugal | yes | 2020-03-27 | EPI_ISL_511117 |
| Portugal/PT0859/2020 | Europe / Portugal |  | 2020-03-27 | EPI_ISL_511118 |
| Portugal/PT0860/2020 | Europe / Portugal | yes | 2020-03-27 | EPI_ISL_511119 |
| Portugal/PT0862/2020 | Europe / Portugal | yes | 2020-03-27 | EPI_ISL_511121 |
| Portugal/PT0863/2020 | Europe / Portugal | yes | 2020-03-27 | EPI_ISL_511122 |
| Portugal/PT0864/2020 | Europe / Portugal | yes | 2020-03-27 | EPI_ISL_511123 |
| Portugal/PT0865/2020 | Europe / Portugal | yes | 2020-03-27 | EPI_ISL_511124 |
| Portugal/PT0866/2020 | Europe / Portugal | yes | 2020-03-27 | EPI_ISL_511125 |
| Portugal/PT0867/2020 | Europe / Portugal | yes | 2020-03-27 | EPI_ISL_511126 |
| Portugal/PT0868/2020 | Europe / Portugal | yes | 2020-03-27 | EPI_ISL_511127 |
| Portugal/PT0869/2020 | Europe / Portugal | yes | 2020-03-27 | EPI_ISL_511128 |
| Portugal/PT0870/2020 | Europe / Portugal | yes | 2020-03-27 | EPI_ISL_511129 |
| Portugal/PT0871/2020 | Europe / Portugal | yes | 2020-03-27 | EPI_ISL_511130 |
| Portugal/PT0872/2020 | Europe / Portugal | yes | 2020-03-27 | EPI_ISL_511131 |
| Portugal/PT0873/2020 | Europe / Portugal | yes | 2020-03-27 | EPI_ISL_511132 |
| Portugal/PT0874/2020 | Europe / Portugal | yes | 2020-03-27 | EPI_ISL_511133 |
| Portugal/PT0875/2020 | Europe / Portugal | yes | 2020-03-27 | EPI_ISL_511134 |
| Portugal/PT0876/2020 | Europe / Portugal | yes | 2020-03-27 | EPI_ISL_511135 |
| Portugal/PT0877/2020 | Europe / Portugal |  | 2020-03-27 | EPI_ISL_511136 |
| Portugal/PT0898/2020 | Europe / Portugal |  | 2020-03-27 | EPI_ISL_511155 |
| Portugal/PT0922/2020 | Europe / Portugal |  | 2020-03-27 | EPI_ISL_511179 |
| Portugal/PT0923/2020 | Europe / Portugal | yes | 2020-03-27 | EPI_ISL_511180 |
| Portugal/PT0933/2020 | Europe / Portugal |  | 2020-03-27 | EPI_ISL_511485 |
| Portugal/PT0934/2020 | Europe / Portugal |  | 2020-03-27 | EPI_ISL_511486 |
| Portugal/PT0973/2020 | Europe / Portugal |  | 2020-03-27 | EPI_ISL_511522 |
| Portugal/PT0974/2020 | Europe / Portugal |  | 2020-03-27 | EPI_ISL_511523 |
| Portugal/PT0975/2020 | Europe / Portugal |  | 2020-03-27 | EPI_ISL_511524 |
| Portugal/PT0976/2020 | Europe / Portugal |  | 2020-03-27 | EPI_ISL_511525 |
| Portugal/PT1006/2020 | Europe / Portugal |  | 2020-03-27 | EPI_ISL_511553 |
| Portugal/PT1007/2020 | Europe / Portugal | yes | 2020-03-27 | EPI_ISL_511554 |
| Portugal/PT1008/2020 | Europe / Portugal |  | 2020-03-27 | EPI_ISL_511555 |
| Portugal/PT1009/2020 | Europe / Portugal | yes | 2020-03-27 | EPI_ISL_511556 |
| Portugal/PT1010/2020 | Europe / Portugal |  | 2020-03-27 | EPI_ISL_511557 |
| Portugal/PT1011/2020 | Europe / Portugal |  | 2020-03-27 | EPI_ISL_511558 |
| Portugal/PT1038/2020 | Europe / Portugal |  | 2020-03-27 | EPI_ISL_511585 |
| Portugal/PT1039/2020 | Europe / Portugal |  | 2020-03-27 | EPI_ISL_511586 |
| Portugal/PT1040/2020 | Europe / Portugal |  | 2020-03-27 | EPI_ISL_511587 |
| Portugal/PT1044/2020 | Europe / Portugal |  | 2020-03-27 | EPI_ISL_511591 |
| Portugal/PT1067/2020 | Europe / Portugal | yes | 2020-03-27 | EPI_ISL_511614 |
| Portugal/PT1069/2020 | Europe / Portugal | yes | 2020-03-27 | EPI_ISL_511616 |
| Portugal/PT1070/2020 | Europe / Portugal | yes | 2020-03-27 | EPI_ISL_511617 |
| Portugal/PT1071/2020 | Europe / Portugal | yes | 2020-03-27 | EPI_ISL_511618 |
| Portugal/PT1072/2020 | Europe / Portugal |  | 2020-03-27 | EPI_ISL_511619 |
| Portugal/PT1075/2020 | Europe / Portugal | yes | 2020-03-27 | EPI_ISL_511622 |
| Portugal/PT1076/2020 | Europe / Portugal |  | 2020-03-27 | EPI_ISL_511623 |
| Portugal/PT1080/2020 | Europe / Portugal |  | 2020-03-27 | EPI_ISL_511627 |
| Portugal/PT1082/2020 | Europe / Portugal | yes | 2020-03-27 | EPI_ISL_511629 |
| Portugal/PT1086/2020 | Europe / Portugal | yes | 2020-03-27 | EPI_ISL_511633 |
| Portugal/PT1089/2020 | Europe / Portugal | yes | 2020-03-27 | EPI_ISL_511636 |
| Portugal/PT1102/2020 | Europe / Portugal |  | 2020-03-27 | EPI_ISL_511649 |
| Portugal/PT1104/2020 | Europe / Portugal |  | 2020-03-27 | EPI_ISL_511651 |
| Portugal/PT1105/2020 | Europe / Portugal | yes | 2020-03-27 | EPI_ISL_511652 |
| Portugal/PT1106/2020 | Europe / Portugal |  | 2020-03-27 | EPI_ISL_511653 |
| Portugal/PT1107/2020 | Europe / Portugal |  | 2020-03-27 | EPI_ISL_511654 |
| Portugal/PT1108/2020 | Europe / Portugal | yes | 2020-03-27 | EPI_ISL_511655 |
| Portugal/PT1111/2020 | Europe / Portugal |  | 2020-03-27 | EPI_ISL_511658 |
| Portugal/PT1112/2020 | Europe / Portugal |  | 2020-03-27 | EPI_ISL_511659 |
| Portugal/PT1113/2020 | Europe / Portugal |  | 2020-03-27 | EPI_ISL_511660 |
| Portugal/PT1114/2020 | Europe / Portugal |  | 2020-03-27 | EPI_ISL_511661 |
| Portugal/PT1115/2020 | Europe / Portugal |  | 2020-03-27 | EPI_ISL_511662 |
| Portugal/PT1132/2020 | Europe / Portugal |  | 2020-03-27 | EPI_ISL_511679 |
| Portugal/PT1133/2020 | Europe / Portugal | yes | 2020-03-27 | EPI_ISL_511680 |
| Portugal/PT1134/2020 | Europe / Portugal | yes | 2020-03-27 | EPI_ISL_511681 |
| Portugal/PT1135/2020 | Europe / Portugal | yes | 2020-03-27 | EPI_ISL_511682 |
| Portugal/PT1139/2020 | Europe / Portugal |  | 2020-03-27 | EPI_ISL_511686 |
| Portugal/PT1190/2020 | Europe / Portugal |  | 2020-03-27 | EPI_ISL_511735 |
| Portugal/PT1220/2020 | Europe / Portugal |  | 2020-03-27 | EPI_ISL_511763 |
| Portugal/PT1348/2020 | Europe / Portugal |  | 2020-03-27 | EPI_ISL_511307 |
| Portugal/PT1370/2020 | Europe / Portugal |  | 2020-03-27 | EPI_ISL_511329 |
| Portugal/PT1381/2020 | Europe / Portugal |  | 2020-03-27 | EPI_ISL_511340 |
| Portugal/PT1382/2020 | Europe / Portugal |  | 2020-03-27 | EPI_ISL_511341 |
| Portugal/PT1383/2020 | Europe / Portugal |  | 2020-03-27 | EPI_ISL_511342 |
| Portugal/PT1384/2020 | Europe / Portugal |  | 2020-03-27 | EPI_ISL_511343 |
| Portugal/PT1385/2020 | Europe / Portugal |  | 2020-03-27 | EPI_ISL_511344 |
| Portugal/PT1386/2020 | Europe / Portugal |  | 2020-03-27 | EPI_ISL_511345 |
| Portugal/PT1387/2020 | Europe / Portugal |  | 2020-03-27 | EPI_ISL_511346 |
| Portugal/PT1388/2020 | Europe / Portugal |  | 2020-03-27 | EPI_ISL_511347 |
| Portugal/PT1389/2020 | Europe / Portugal |  | 2020-03-27 | EPI_ISL_511348 |
| Portugal/PT1390/2020 | Europe / Portugal |  | 2020-03-27 | EPI_ISL_511349 |
| Portugal/PT1391/2020 | Europe / Portugal |  | 2020-03-27 | EPI_ISL_511350 |
| Portugal/PT1392/2020 | Europe / Portugal |  | 2020-03-27 | EPI_ISL_511351 |
| Portugal/PT1393/2020 | Europe / Portugal |  | 2020-03-27 | EPI_ISL_511352 |
| Portugal/PT1394/2020 | Europe / Portugal |  | 2020-03-27 | EPI_ISL_511353 |
| Portugal/PT1395/2020 | Europe / Portugal |  | 2020-03-27 | EPI_ISL_511354 |
| Portugal/PT1396/2020 | Europe / Portugal |  | 2020-03-27 | EPI_ISL_511355 |
| Portugal/PT1397/2020 | Europe / Portugal |  | 2020-03-27 | EPI_ISL_511356 |
| Portugal/PT1406/2020 | Europe / Portugal |  | 2020-03-27 | EPI_ISL_511365 |
| Portugal/PT1417/2020 | Europe / Portugal | yes | 2020-03-27 | EPI_ISL_511376 |
| Portugal/PT1423/2020 | Europe / Portugal |  | 2020-03-27 | EPI_ISL_511382 |
| Portugal/PT1424/2020 | Europe / Portugal | yes | 2020-03-27 | EPI_ISL_511383 |
| Portugal/PT1425/2020 | Europe / Portugal |  | 2020-03-27 | EPI_ISL_511384 |
| Portugal/PT1440/2020 | Europe / Portugal |  | 2020-03-27 | EPI_ISL_511399 |
| Portugal/PT1447/2020 | Europe / Portugal |  | 2020-03-27 | EPI_ISL_511406 |
| Portugal/PT1450/2020 | Europe / Portugal |  | 2020-03-27 | EPI_ISL_511409 |
| Portugal/PT1461/2020 | Europe / Portugal |  | 2020-03-27 | EPI_ISL_511420 |
| Portugal/PT1479/2020 | Europe / Portugal |  | 2020-03-27 | EPI_ISL_511438 |
| Portugal/PT1495/2020 | Europe / Portugal | yes | 2020-03-27 | EPI_ISL_511454 |
| Portugal/PT0148/2020 | Europe / Portugal |  | 2020-03-28 | EPI_ISL_453864 |
| Portugal/PT0152/2020 | Europe / Portugal |  | 2020-03-28 | EPI_ISL_453868 |
| Portugal/PT0153/2020 | Europe / Portugal |  | 2020-03-28 | EPI_ISL_453869 |
| Portugal/PT0161/2020 | Europe / Portugal |  | 2020-03-28 | EPI_ISL_453877 |
| Portugal/PT0162/2020 | Europe / Portugal |  | 2020-03-28 | EPI_ISL_453878 |
| Portugal/PT0163/2020 | Europe / Portugal |  | 2020-03-28 | EPI_ISL_453879 |
| Portugal/PT0164/2020 | Europe / Portugal |  | 2020-03-28 | EPI_ISL_453880 |
| Portugal/PT0165/2020 | Europe / Portugal | yes | 2020-03-28 | EPI_ISL_453881 |
| Portugal/PT0166/2020 | Europe / Portugal | yes | 2020-03-28 | EPI_ISL_453882 |
| Portugal/PT0167/2020 | Europe / Portugal | yes | 2020-03-28 | EPI_ISL_453883 |
| Portugal/PT0168/2020 | Europe / Portugal | yes | 2020-03-28 | EPI_ISL_453884 |
| Portugal/PT0169/2020 | Europe / Portugal | yes | 2020-03-28 | EPI_ISL_453885 |
| Portugal/PT0170/2020 | Europe / Portugal | yes | 2020-03-28 | EPI_ISL_453886 |
| Portugal/PT0171/2020 | Europe / Portugal |  | 2020-03-28 | EPI_ISL_453887 |
| Portugal/PT0172/2020 | Europe / Portugal |  | 2020-03-28 | EPI_ISL_453888 |
| Portugal/PT0173/2020 | Europe / Portugal |  | 2020-03-28 | EPI_ISL_453889 |
| Portugal/PT0174/2020 | Europe / Portugal |  | 2020-03-28 | EPI_ISL_453890 |
| Portugal/PT0175/2020 | Europe / Portugal |  | 2020-03-28 | EPI_ISL_453891 |
| Portugal/PT0176/2020 | Europe / Portugal |  | 2020-03-28 | EPI_ISL_453892 |
| Portugal/PT0177/2020 | Europe / Portugal |  | 2020-03-28 | EPI_ISL_453893 |
| Portugal/PT0178/2020 | Europe / Portugal |  | 2020-03-28 | EPI_ISL_453894 |
| Portugal/PT0179/2020 | Europe / Portugal |  | 2020-03-28 | EPI_ISL_453895 |
| Portugal/PT0180/2020 | Europe / Portugal |  | 2020-03-28 | EPI_ISL_453896 |
| Portugal/PT0181/2020 | Europe / Portugal |  | 2020-03-28 | EPI_ISL_453897 |
| Portugal/PT0182/2020 | Europe / Portugal |  | 2020-03-28 | EPI_ISL_453898 |
| Portugal/PT0183/2020 | Europe / Portugal |  | 2020-03-28 | EPI_ISL_453899 |
| Portugal/PT0184/2020 | Europe / Portugal |  | 2020-03-28 | EPI_ISL_453900 |
| Portugal/PT0185/2020 | Europe / Portugal |  | 2020-03-28 | EPI_ISL_453901 |
| Portugal/PT0199/2020 | Europe / Portugal |  | 2020-03-28 | EPI_ISL_453915 |
| Portugal/PT0421/2020 | Europe / Portugal |  | 2020-03-28 | EPI_ISL_454145 |
| Portugal/PT0422/2020 | Europe / Portugal |  | 2020-03-28 | EPI_ISL_454146 |
| Portugal/PT0423/2020 | Europe / Portugal |  | 2020-03-28 | EPI_ISL_454147 |
| Portugal/PT0424/2020 | Europe / Portugal |  | 2020-03-28 | EPI_ISL_454148 |
| Portugal/PT0425/2020 | Europe / Portugal |  | 2020-03-28 | EPI_ISL_454149 |
| Portugal/PT0426/2020 | Europe / Portugal |  | 2020-03-28 | EPI_ISL_454150 |
| Portugal/PT0427/2020 | Europe / Portugal |  | 2020-03-28 | EPI_ISL_454151 |
| Portugal/PT0428/2020 | Europe / Portugal |  | 2020-03-28 | EPI_ISL_454152 |
| Portugal/PT0448/2020 | Europe / Portugal |  | 2020-03-28 | EPI_ISL_454172 |
| Portugal/PT0449/2020 | Europe / Portugal |  | 2020-03-28 | EPI_ISL_454173 |
| Portugal/PT0481/2020 | Europe / Portugal |  | 2020-03-28 | EPI_ISL_454205 |
| Portugal/PT0588/2020 | Europe / Portugal |  | 2020-03-28 | EPI_ISL_454311 |
| Portugal/PT0589/2020 | Europe / Portugal |  | 2020-03-28 | EPI_ISL_454312 |
| Portugal/PT0590/2020 | Europe / Portugal |  | 2020-03-28 | EPI_ISL_454313 |
| Portugal/PT0591/2020 | Europe / Portugal |  | 2020-03-28 | EPI_ISL_454314 |
| Portugal/PT0596/2020 | Europe / Portugal |  | 2020-03-28 | EPI_ISL_454319 |
| Portugal/PT0597/2020 | Europe / Portugal |  | 2020-03-28 | EPI_ISL_454320 |
| Portugal/PT0598/2020 | Europe / Portugal |  | 2020-03-28 | EPI_ISL_454321 |
| Portugal/PT0599/2020 | Europe / Portugal |  | 2020-03-28 | EPI_ISL_454322 |
| Portugal/PT0636/2020 | Europe / Portugal | yes | 2020-03-28 | EPI_ISL_510897 |
| Portugal/PT0637/2020 | Europe / Portugal |  | 2020-03-28 | EPI_ISL_510898 |
| Portugal/PT0638/2020 | Europe / Portugal | yes | 2020-03-28 | EPI_ISL_510899 |
| Portugal/PT0639/2020 | Europe / Portugal |  | 2020-03-28 | EPI_ISL_510900 |
| Portugal/PT0655/2020 | Europe / Portugal |  | 2020-03-28 | EPI_ISL_510916 |
| Portugal/PT0656/2020 | Europe / Portugal | yes | 2020-03-28 | EPI_ISL_510917 |
| Portugal/PT0658/2020 | Europe / Portugal | yes | 2020-03-28 | EPI_ISL_510918 |
| Portugal/PT0681/2020 | Europe / Portugal |  | 2020-03-28 | EPI_ISL_510940 |
| Portugal/PT0861/2020 | Europe / Portugal | yes | 2020-03-28 | EPI_ISL_511120 |
| Portugal/PT0878/2020 | Europe / Portugal | yes | 2020-03-28 | EPI_ISL_511137 |
| Portugal/PT0879/2020 | Europe / Portugal | yes | 2020-03-28 | EPI_ISL_511138 |
| Portugal/PT0899/2020 | Europe / Portugal |  | 2020-03-28 | EPI_ISL_511156 |
| Portugal/PT0900/2020 | Europe / Portugal |  | 2020-03-28 | EPI_ISL_511157 |
| Portugal/PT0901/2020 | Europe / Portugal |  | 2020-03-28 | EPI_ISL_511158 |
| Portugal/PT0902/2020 | Europe / Portugal |  | 2020-03-28 | EPI_ISL_511159 |
| Portugal/PT0903/2020 | Europe / Portugal |  | 2020-03-28 | EPI_ISL_511160 |
| Portugal/PT0904/2020 | Europe / Portugal | yes | 2020-03-28 | EPI_ISL_511161 |
| Portugal/PT0905/2020 | Europe / Portugal | yes | 2020-03-28 | EPI_ISL_511162 |
| Portugal/PT0906/2020 | Europe / Portugal |  | 2020-03-28 | EPI_ISL_511163 |
| Portugal/PT0907/2020 | Europe / Portugal |  | 2020-03-28 | EPI_ISL_511164 |
| Portugal/PT0908/2020 | Europe / Portugal |  | 2020-03-28 | EPI_ISL_511165 |
| Portugal/PT0909/2020 | Europe / Portugal |  | 2020-03-28 | EPI_ISL_511166 |
| Portugal/PT0910/2020 | Europe / Portugal |  | 2020-03-28 | EPI_ISL_511167 |
| Portugal/PT0911/2020 | Europe / Portugal |  | 2020-03-28 | EPI_ISL_511168 |
| Portugal/PT0912/2020 | Europe / Portugal |  | 2020-03-28 | EPI_ISL_511169 |
| Portugal/PT0913/2020 | Europe / Portugal |  | 2020-03-28 | EPI_ISL_511170 |
| Portugal/PT0914/2020 | Europe / Portugal |  | 2020-03-28 | EPI_ISL_511171 |
| Portugal/PT0915/2020 | Europe / Portugal |  | 2020-03-28 | EPI_ISL_511172 |
| Portugal/PT0916/2020 | Europe / Portugal |  | 2020-03-28 | EPI_ISL_511173 |
| Portugal/PT0917/2020 | Europe / Portugal |  | 2020-03-28 | EPI_ISL_511174 |
| Portugal/PT0918/2020 | Europe / Portugal |  | 2020-03-28 | EPI_ISL_511175 |
| Portugal/PT0969/2020 | Europe / Portugal |  | 2020-03-28 | EPI_ISL_511518 |
| Portugal/PT0970/2020 | Europe / Portugal |  | 2020-03-28 | EPI_ISL_511519 |
| Portugal/PT0971/2020 | Europe / Portugal |  | 2020-03-28 | EPI_ISL_511520 |
| Portugal/PT1041/2020 | Europe / Portugal |  | 2020-03-28 | EPI_ISL_511588 |
| Portugal/PT1042/2020 | Europe / Portugal |  | 2020-03-28 | EPI_ISL_511589 |
| Portugal/PT1043/2020 | Europe / Portugal |  | 2020-03-28 | EPI_ISL_511590 |
| Portugal/PT1045/2020 | Europe / Portugal |  | 2020-03-28 | EPI_ISL_511592 |
| Portugal/PT1046/2020 | Europe / Portugal |  | 2020-03-28 | EPI_ISL_511593 |
| Portugal/PT1047/2020 | Europe / Portugal |  | 2020-03-28 | EPI_ISL_511594 |
| Portugal/PT1048/2020 | Europe / Portugal |  | 2020-03-28 | EPI_ISL_511595 |
| Portugal/PT1049/2020 | Europe / Portugal | yes | 2020-03-28 | EPI_ISL_511596 |
| Portugal/PT1050/2020 | Europe / Portugal |  | 2020-03-28 | EPI_ISL_511597 |
| Portugal/PT1051/2020 | Europe / Portugal |  | 2020-03-28 | EPI_ISL_511598 |
| Portugal/PT1052/2020 | Europe / Portugal | yes | 2020-03-28 | EPI_ISL_511599 |
| Portugal/PT1053/2020 | Europe / Portugal |  | 2020-03-28 | EPI_ISL_511600 |
| Portugal/PT1054/2020 | Europe / Portugal |  | 2020-03-28 | EPI_ISL_511601 |
| Portugal/PT1055/2020 | Europe / Portugal |  | 2020-03-28 | EPI_ISL_511602 |
| Portugal/PT1059/2020 | Europe / Portugal |  | 2020-03-28 | EPI_ISL_511606 |
| Portugal/PT1064/2020 | Europe / Portugal | yes | 2020-03-28 | EPI_ISL_511611 |
| Portugal/PT1065/2020 | Europe / Portugal | yes | 2020-03-28 | EPI_ISL_511612 |
| Portugal/PT1066/2020 | Europe / Portugal | yes | 2020-03-28 | EPI_ISL_511613 |
| Portugal/PT1068/2020 | Europe / Portugal |  | 2020-03-28 | EPI_ISL_511615 |
| Portugal/PT1073/2020 | Europe / Portugal | yes | 2020-03-28 | EPI_ISL_511620 |
| Portugal/PT1074/2020 | Europe / Portugal | yes | 2020-03-28 | EPI_ISL_511621 |
| Portugal/PT1077/2020 | Europe / Portugal | yes | 2020-03-28 | EPI_ISL_511624 |
| Portugal/PT1078/2020 | Europe / Portugal | yes | 2020-03-28 | EPI_ISL_511625 |
| Portugal/PT1079/2020 | Europe / Portugal | yes | 2020-03-28 | EPI_ISL_511626 |
| Portugal/PT1081/2020 | Europe / Portugal | yes | 2020-03-28 | EPI_ISL_511628 |
| Portugal/PT1083/2020 | Europe / Portugal | yes | 2020-03-28 | EPI_ISL_511630 |
| Portugal/PT1084/2020 | Europe / Portugal | yes | 2020-03-28 | EPI_ISL_511631 |
| Portugal/PT1085/2020 | Europe / Portugal | yes | 2020-03-28 | EPI_ISL_511632 |
| Portugal/PT1087/2020 | Europe / Portugal | yes | 2020-03-28 | EPI_ISL_511634 |
| Portugal/PT1088/2020 | Europe / Portugal |  | 2020-03-28 | EPI_ISL_511635 |
| Portugal/PT1090/2020 | Europe / Portugal | yes | 2020-03-28 | EPI_ISL_511637 |
| Portugal/PT1091/2020 | Europe / Portugal | yes | 2020-03-28 | EPI_ISL_511638 |
| Portugal/PT1092/2020 | Europe / Portugal |  | 2020-03-28 | EPI_ISL_511639 |
| Portugal/PT1093/2020 | Europe / Portugal | yes | 2020-03-28 | EPI_ISL_511640 |
| Portugal/PT1094/2020 | Europe / Portugal |  | 2020-03-28 | EPI_ISL_511641 |
| Portugal/PT1100/2020 | Europe / Portugal |  | 2020-03-28 | EPI_ISL_511647 |
| Portugal/PT1103/2020 | Europe / Portugal | yes | 2020-03-28 | EPI_ISL_511650 |
| Portugal/PT1127/2020 | Europe / Portugal |  | 2020-03-28 | EPI_ISL_511674 |
| Portugal/PT1189/2020 | Europe / Portugal |  | 2020-03-28 | EPI_ISL_511734 |
| Portugal/PT1216/2020 | Europe / Portugal |  | 2020-03-28 | EPI_ISL_511759 |
| Portugal/PT1217/2020 | Europe / Portugal |  | 2020-03-28 | EPI_ISL_511760 |
| Portugal/PT1218/2020 | Europe / Portugal |  | 2020-03-28 | EPI_ISL_511761 |
| Portugal/PT1221/2020 | Europe / Portugal |  | 2020-03-28 | EPI_ISL_511764 |
| Portugal/PT1222/2020 | Europe / Portugal |  | 2020-03-28 | EPI_ISL_511765 |
| Portugal/PT1398/2020 | Europe / Portugal |  | 2020-03-28 | EPI_ISL_511357 |
| Portugal/PT1399/2020 | Europe / Portugal |  | 2020-03-28 | EPI_ISL_511358 |
| Portugal/PT1400/2020 | Europe / Portugal |  | 2020-03-28 | EPI_ISL_511359 |
| Portugal/PT1411/2020 | Europe / Portugal |  | 2020-03-28 | EPI_ISL_511370 |
| Portugal/PT1429/2020 | Europe / Portugal | yes | 2020-03-28 | EPI_ISL_511388 |
| Portugal/PT1462/2020 | Europe / Portugal |  | 2020-03-28 | EPI_ISL_511421 |
| Portugal/PT1464/2020 | Europe / Portugal | yes | 2020-03-28 | EPI_ISL_511423 |
| Portugal/PT1490/2020 | Europe / Portugal |  | 2020-03-28 | EPI_ISL_511449 |
| Portugal/PT1505/2020 | Europe / Portugal |  | 2020-03-28 | EPI_ISL_511464 |
| Portugal/PT1510/2020 | Europe / Portugal |  | 2020-03-28 | EPI_ISL_511469 |
| Portugal/PT0186/2020 | Europe / Portugal |  | 2020-03-29 | EPI_ISL_453902 |
| Portugal/PT0187/2020 | Europe / Portugal |  | 2020-03-29 | EPI_ISL_453903 |
| Portugal/PT0188/2020 | Europe / Portugal | yes | 2020-03-29 | EPI_ISL_453904 |
| Portugal/PT0189/2020 | Europe / Portugal | yes | 2020-03-29 | EPI_ISL_453905 |
| Portugal/PT0190/2020 | Europe / Portugal | yes | 2020-03-29 | EPI_ISL_453906 |
| Portugal/PT0191/2020 | Europe / Portugal | yes | 2020-03-29 | EPI_ISL_453907 |
| Portugal/PT0192/2020 | Europe / Portugal |  | 2020-03-29 | EPI_ISL_453908 |
| Portugal/PT0193/2020 | Europe / Portugal | yes | 2020-03-29 | EPI_ISL_453909 |
| Portugal/PT0194/2020 | Europe / Portugal | yes | 2020-03-29 | EPI_ISL_453910 |
| Portugal/PT0195/2020 | Europe / Portugal | yes | 2020-03-29 | EPI_ISL_453911 |
| Portugal/PT0196/2020 | Europe / Portugal | yes | 2020-03-29 | EPI_ISL_453912 |
| Portugal/PT0197/2020 | Europe / Portugal | yes | 2020-03-29 | EPI_ISL_453913 |
| Portugal/PT0198/2020 | Europe / Portugal |  | 2020-03-29 | EPI_ISL_453914 |
| Portugal/PT0200/2020 | Europe / Portugal |  | 2020-03-29 | EPI_ISL_453916 |
| Portugal/PT0201/2020 | Europe / Portugal |  | 2020-03-29 | EPI_ISL_455625 |
| Portugal/PT0202/2020 | Europe / Portugal |  | 2020-03-29 | EPI_ISL_455626 |
| Portugal/PT0203/2020 | Europe / Portugal |  | 2020-03-29 | EPI_ISL_453917 |
| Portugal/PT0482/2020 | Europe / Portugal | yes | 2020-03-29 | EPI_ISL_454206 |
| Portugal/PT0574/2020 | Europe / Portugal |  | 2020-03-29 | EPI_ISL_454297 |
| Portugal/PT0600/2020 | Europe / Portugal |  | 2020-03-29 | EPI_ISL_454323 |
| Portugal/PT0626/2020 | Europe / Portugal | yes | 2020-03-29 | EPI_ISL_454349 |
| Portugal/PT0640/2020 | Europe / Portugal |  | 2020-03-29 | EPI_ISL_510901 |
| Portugal/PT0641/2020 | Europe / Portugal | yes | 2020-03-29 | EPI_ISL_510902 |
| Portugal/PT0642/2020 | Europe / Portugal |  | 2020-03-29 | EPI_ISL_510903 |
| Portugal/PT0643/2020 | Europe / Portugal |  | 2020-03-29 | EPI_ISL_510904 |
| Portugal/PT0644/2020 | Europe / Portugal |  | 2020-03-29 | EPI_ISL_510905 |
| Portugal/PT0645/2020 | Europe / Portugal | yes | 2020-03-29 | EPI_ISL_510906 |
| Portugal/PT0660/2020 | Europe / Portugal |  | 2020-03-29 | EPI_ISL_510919 |
| Portugal/PT0661/2020 | Europe / Portugal | yes | 2020-03-29 | EPI_ISL_510920 |
| Portugal/PT0919/2020 | Europe / Portugal |  | 2020-03-29 | EPI_ISL_511176 |
| Portugal/PT0920/2020 | Europe / Portugal |  | 2020-03-29 | EPI_ISL_511177 |
| Portugal/PT0967/2020 | Europe / Portugal |  | 2020-03-29 | EPI_ISL_511516 |
| Portugal/PT0968/2020 | Europe / Portugal |  | 2020-03-29 | EPI_ISL_511517 |
| Portugal/PT0972/2020 | Europe / Portugal |  | 2020-03-29 | EPI_ISL_511521 |
| Portugal/PT1056/2020 | Europe / Portugal |  | 2020-03-29 | EPI_ISL_511603 |
| Portugal/PT1057/2020 | Europe / Portugal |  | 2020-03-29 | EPI_ISL_511604 |
| Portugal/PT1058/2020 | Europe / Portugal |  | 2020-03-29 | EPI_ISL_511605 |
| Portugal/PT1060/2020 | Europe / Portugal | yes | 2020-03-29 | EPI_ISL_511607 |
| Portugal/PT1061/2020 | Europe / Portugal | yes | 2020-03-29 | EPI_ISL_511608 |
| Portugal/PT1101/2020 | Europe / Portugal |  | 2020-03-29 | EPI_ISL_511648 |
| Portugal/PT0530a/2020 | Europe / Portugal | yes | 2020-03-29 | EPI_ISL_511480 |
| Portugal/PT1191/2020 | Europe / Portugal |  | 2020-03-29 | EPI_ISL_582520 |
| Portugal/PT1223/2020 | Europe / Portugal |  | 2020-03-29 | EPI_ISL_511766 |
| Portugal/PT1224/2020 | Europe / Portugal |  | 2020-03-29 | EPI_ISL_511767 |
| Portugal/PT1225/2020 | Europe / Portugal |  | 2020-03-29 | EPI_ISL_511768 |
| Portugal/PT1226/2020 | Europe / Portugal |  | 2020-03-29 | EPI_ISL_511769 |
| Portugal/PT1228/2020 | Europe / Portugal |  | 2020-03-29 | EPI_ISL_511771 |
| Portugal/PT1229/2020 | Europe / Portugal |  | 2020-03-29 | EPI_ISL_511190 |
| Portugal/PT1401/2020 | Europe / Portugal |  | 2020-03-29 | EPI_ISL_511360 |
| Portugal/PT1445/2020 | Europe / Portugal |  | 2020-03-29 | EPI_ISL_511404 |
| Portugal/PT1460/2020 | Europe / Portugal |  | 2020-03-29 | EPI_ISL_511419 |
| Portugal/PT1507/2020 | Europe / Portugal |  | 2020-03-29 | EPI_ISL_511466 |
| Portugal/PT0206/2020 | Europe / Portugal |  | 2020-03-30 | EPI_ISL_453920 |
| Portugal/PT0207/2020 | Europe / Portugal |  | 2020-03-30 | EPI_ISL_453921 |
| Portugal/PT0208/2020 | Europe / Portugal |  | 2020-03-30 | EPI_ISL_453922 |
| Portugal/PT0209/2020 | Europe / Portugal |  | 2020-03-30 | EPI_ISL_453923 |
| Portugal/PT0210/2020 | Europe / Portugal |  | 2020-03-30 | EPI_ISL_453924 |
| Portugal/PT0211/2020 | Europe / Portugal |  | 2020-03-30 | EPI_ISL_453925 |
| Portugal/PT0212/2020 | Europe / Portugal |  | 2020-03-30 | EPI_ISL_453926 |
| Portugal/PT0213/2020 | Europe / Portugal |  | 2020-03-30 | EPI_ISL_453927 |
| Portugal/PT0214/2020 | Europe / Portugal |  | 2020-03-30 | EPI_ISL_453928 |
| Portugal/PT0215/2020 | Europe / Portugal |  | 2020-03-30 | EPI_ISL_453929 |
| Portugal/PT0217/2020 | Europe / Portugal | yes | 2020-03-30 | EPI_ISL_453931 |
| Portugal/PT0218/2020 | Europe / Portugal | yes | 2020-03-30 | EPI_ISL_453932 |
| Portugal/PT0219/2020 | Europe / Portugal | yes | 2020-03-30 | EPI_ISL_453933 |
| Portugal/PT0220/2020 | Europe / Portugal | yes | 2020-03-30 | EPI_ISL_453934 |
| Portugal/PT0221/2020 | Europe / Portugal | yes | 2020-03-30 | EPI_ISL_453935 |
| Portugal/PT0222/2020 | Europe / Portugal |  | 2020-03-30 | EPI_ISL_453936 |
| Portugal/PT0223/2020 | Europe / Portugal |  | 2020-03-30 | EPI_ISL_453937 |
| Portugal/PT0483/2020 | Europe / Portugal |  | 2020-03-30 | EPI_ISL_454207 |
| Portugal/PT0485/2020 | Europe / Portugal |  | 2020-03-30 | EPI_ISL_454209 |
| Portugal/PT0486/2020 | Europe / Portugal |  | 2020-03-30 | EPI_ISL_454210 |
| Portugal/PT0592/2020 | Europe / Portugal |  | 2020-03-30 | EPI_ISL_454315 |
| Portugal/PT0646/2020 | Europe / Portugal | yes | 2020-03-30 | EPI_ISL_510907 |
| Portugal/PT0647/2020 | Europe / Portugal |  | 2020-03-30 | EPI_ISL_510908 |
| Portugal/PT0685/2020 | Europe / Portugal |  | 2020-03-30 | EPI_ISL_510944 |
| Portugal/PT0686/2020 | Europe / Portugal |  | 2020-03-30 | EPI_ISL_510945 |
| Portugal/PT0977/2020 | Europe / Portugal |  | 2020-03-30 | EPI_ISL_511526 |
| Portugal/PT0978/2020 | Europe / Portugal |  | 2020-03-30 | EPI_ISL_511527 |
| Portugal/PT0979/2020 | Europe / Portugal |  | 2020-03-30 | EPI_ISL_511528 |
| Portugal/PT1062/2020 | Europe / Portugal |  | 2020-03-30 | EPI_ISL_511609 |
| Portugal/PT1063/2020 | Europe / Portugal |  | 2020-03-30 | EPI_ISL_511610 |
| Portugal/PT1110/2020 | Europe / Portugal |  | 2020-03-30 | EPI_ISL_511657 |
| Portugal/PT1117/2020 | Europe / Portugal | yes | 2020-03-30 | EPI_ISL_511664 |
| Portugal/PT1118/2020 | Europe / Portugal | yes | 2020-03-30 | EPI_ISL_511665 |
| Portugal/PT1119/2020 | Europe / Portugal |  | 2020-03-30 | EPI_ISL_511666 |
| Portugal/PT1120/2020 | Europe / Portugal |  | 2020-03-30 | EPI_ISL_511667 |
| Portugal/PT1123/2020 | Europe / Portugal |  | 2020-03-30 | EPI_ISL_511670 |
| Portugal/PT1192/2020 | Europe / Portugal |  | 2020-03-30 | EPI_ISL_511736 |
| Portugal/PT1205/2020 | Europe / Portugal | yes | 2020-03-30 | EPI_ISL_511748 |
| Portugal/PT1206/2020 | Europe / Portugal |  | 2020-03-30 | EPI_ISL_511749 |
| Portugal/PT1207/2020 | Europe / Portugal | yes | 2020-03-30 | EPI_ISL_511750 |
| Portugal/PT1230/2020 | Europe / Portugal |  | 2020-03-30 | EPI_ISL_511191 |
| Portugal/PT1416/2020 | Europe / Portugal |  | 2020-03-30 | EPI_ISL_511375 |
| Portugal/PT1428/2020 | Europe / Portugal |  | 2020-03-30 | EPI_ISL_511387 |
| Portugal/PT1436/2020 | Europe / Portugal |  | 2020-03-30 | EPI_ISL_511395 |
| Portugal/PT1448/2020 | Europe / Portugal |  | 2020-03-30 | EPI_ISL_511407 |
| Portugal/PT1455/2020 | Europe / Portugal |  | 2020-03-30 | EPI_ISL_511414 |
| Portugal/PT1456/2020 | Europe / Portugal |  | 2020-03-30 | EPI_ISL_511415 |
| Portugal/PT1463/2020 | Europe / Portugal |  | 2020-03-30 | EPI_ISL_511422 |
| Portugal/PT1465/2020 | Europe / Portugal | yes | 2020-03-30 | EPI_ISL_511424 |
| Portugal/PT1477/2020 | Europe / Portugal |  | 2020-03-30 | EPI_ISL_511436 |
| Portugal/PT1480/2020 | Europe / Portugal |  | 2020-03-30 | EPI_ISL_511439 |
| Portugal/PT1483/2020 | Europe / Portugal | yes | 2020-03-30 | EPI_ISL_511442 |
| Portugal/PT1494/2020 | Europe / Portugal | yes | 2020-03-30 | EPI_ISL_511453 |
| Portugal/PT1498/2020 | Europe / Portugal | yes | 2020-03-30 | EPI_ISL_511457 |
| Portugal/PT1502/2020 | Europe / Portugal |  | 2020-03-30 | EPI_ISL_511461 |
| Portugal/PT0224/2020 | Europe / Portugal |  | 2020-03-31 | EPI_ISL_453938 |
| Portugal/PT0225/2020 | Europe / Portugal |  | 2020-03-31 | EPI_ISL_453939 |
| Portugal/PT0226/2020 | Europe / Portugal |  | 2020-03-31 | EPI_ISL_453940 |
| Portugal/PT0227/2020 | Europe / Portugal |  | 2020-03-31 | EPI_ISL_453941 |
| Portugal/PT0228/2020 | Europe / Portugal | yes | 2020-03-31 | EPI_ISL_453942 |
| Portugal/PT0229/2020 | Europe / Portugal | yes | 2020-03-31 | EPI_ISL_453943 |
| Portugal/PT0230/2020 | Europe / Portugal | yes | 2020-03-31 | EPI_ISL_453944 |
| Portugal/PT0231/2020 | Europe / Portugal | yes | 2020-03-31 | EPI_ISL_453945 |
| Portugal/PT0232/2020 | Europe / Portugal | yes | 2020-03-31 | EPI_ISL_453946 |
| Portugal/PT0233/2020 | Europe / Portugal | yes | 2020-03-31 | EPI_ISL_453947 |
| Portugal/PT0234/2020 | Europe / Portugal | yes | 2020-03-31 | EPI_ISL_453948 |
| Portugal/PT0235/2020 | Europe / Portugal | yes | 2020-03-31 | EPI_ISL_453949 |
| Portugal/PT0236/2020 | Europe / Portugal | yes | 2020-03-31 | EPI_ISL_453950 |
| Portugal/PT0237/2020 | Europe / Portugal | yes | 2020-03-31 | EPI_ISL_453951 |
| Portugal/PT0238/2020 | Europe / Portugal | yes | 2020-03-31 | EPI_ISL_453952 |
| Portugal/PT0239/2020 | Europe / Portugal | yes | 2020-03-31 | EPI_ISL_453953 |
| Portugal/PT0240/2020 | Europe / Portugal |  | 2020-03-31 | EPI_ISL_453954 |
| Portugal/PT0241/2020 | Europe / Portugal | yes | 2020-03-31 | EPI_ISL_453955 |
| Portugal/PT0242/2020 | Europe / Portugal | yes | 2020-03-31 | EPI_ISL_453956 |
| Portugal/PT0243/2020 | Europe / Portugal | yes | 2020-03-31 | EPI_ISL_453957 |
| Portugal/PT0244/2020 | Europe / Portugal |  | 2020-03-31 | EPI_ISL_453958 |
| Portugal/PT0429/2020 | Europe / Portugal |  | 2020-03-31 | EPI_ISL_454153 |
| Portugal/PT0430/2020 | Europe / Portugal |  | 2020-03-31 | EPI_ISL_454154 |
| Portugal/PT0450/2020 | Europe / Portugal |  | 2020-03-31 | EPI_ISL_454174 |
| Portugal/PT0484/2020 | Europe / Portugal |  | 2020-03-31 | EPI_ISL_454208 |
| Portugal/PT0488/2020 | Europe / Portugal |  | 2020-03-31 | EPI_ISL_454212 |
| Portugal/PT0627/2020 | Europe / Portugal |  | 2020-03-31 | EPI_ISL_454350 |
| Portugal/PT0648/2020 | Europe / Portugal |  | 2020-03-31 | EPI_ISL_510909 |
| Portugal/PT0649/2020 | Europe / Portugal |  | 2020-03-31 | EPI_ISL_510910 |
| Portugal/PT0650/2020 | Europe / Portugal | yes | 2020-03-31 | EPI_ISL_510911 |
| Portugal/PT0651/2020 | Europe / Portugal | yes | 2020-03-31 | EPI_ISL_510912 |
| Portugal/PT0668/2020 | Europe / Portugal |  | 2020-03-31 | EPI_ISL_510927 |
| Portugal/PT0687/2020 | Europe / Portugal |  | 2020-03-31 | EPI_ISL_510946 |
| Portugal/PT0921/2020 | Europe / Portugal | yes | 2020-03-31 | EPI_ISL_511178 |
| Portugal/PT0980/2020 | Europe / Portugal |  | 2020-03-31 | EPI_ISL_511529 |
| Portugal/PT1109/2020 | Europe / Portugal |  | 2020-03-31 | EPI_ISL_511656 |
| Portugal/PT1116/2020 | Europe / Portugal |  | 2020-03-31 | EPI_ISL_511663 |
| Portugal/PT1121/2020 | Europe / Portugal |  | 2020-03-31 | EPI_ISL_511668 |
| Portugal/PT1122/2020 | Europe / Portugal |  | 2020-03-31 | EPI_ISL_511669 |
| Portugal/PT1128/2020 | Europe / Portugal |  | 2020-03-31 | EPI_ISL_511675 |
| Portugal/PT1140/2020 | Europe / Portugal |  | 2020-03-31 | EPI_ISL_511687 |
| Portugal/PT1141/2020 | Europe / Portugal |  | 2020-03-31 | EPI_ISL_511688 |
| Portugal/PT1193/2020 | Europe / Portugal | yes | 2020-03-31 | EPI_ISL_511737 |
| Portugal/PT1194/2020 | Europe / Portugal | yes | 2020-03-31 | EPI_ISL_511738 |
| Portugal/PT1195/2020 | Europe / Portugal | yes | 2020-03-31 | EPI_ISL_511739 |
| Portugal/PT1196/2020 | Europe / Portugal | yes | 2020-03-31 | EPI_ISL_511740 |
| Portugal/PT1197/2020 | Europe / Portugal | yes | 2020-03-31 | EPI_ISL_511741 |
| Portugal/PT1198/2020 | Europe / Portugal |  | 2020-03-31 | EPI_ISL_511742 |
| Portugal/PT1199/2020 | Europe / Portugal |  | 2020-03-31 | EPI_ISL_582521 |
| Portugal/PT1200/2020 | Europe / Portugal | yes | 2020-03-31 | EPI_ISL_511743 |
| Portugal/PT1201/2020 | Europe / Portugal | yes | 2020-03-31 | EPI_ISL_511744 |
| Portugal/PT1202/2020 | Europe / Portugal | yes | 2020-03-31 | EPI_ISL_511745 |
| Portugal/PT1203/2020 | Europe / Portugal | yes | 2020-03-31 | EPI_ISL_511746 |
| Portugal/PT1204/2020 | Europe / Portugal | yes | 2020-03-31 | EPI_ISL_511747 |
| Portugal/PT1231/2020 | Europe / Portugal |  | 2020-03-31 | EPI_ISL_511192 |
| Portugal/PT1232/2020 | Europe / Portugal |  | 2020-03-31 | EPI_ISL_511193 |
| Portugal/PT1233/2020 | Europe / Portugal |  | 2020-03-31 | EPI_ISL_511194 |
| Portugal/PT1234/2020 | Europe / Portugal |  | 2020-03-31 | EPI_ISL_511195 |
| Portugal/PT1235/2020 | Europe / Portugal |  | 2020-03-31 | EPI_ISL_511196 |
| Portugal/PT1236/2020 | Europe / Portugal |  | 2020-03-31 | EPI_ISL_511197 |
| Portugal/PT1237/2020 | Europe / Portugal |  | 2020-03-31 | EPI_ISL_511198 |
| Portugal/PT1238/2020 | Europe / Portugal |  | 2020-03-31 | EPI_ISL_511199 |
| Portugal/PT1239/2020 | Europe / Portugal |  | 2020-03-31 | EPI_ISL_511200 |
| Portugal/PT1240/2020 | Europe / Portugal |  | 2020-03-31 | EPI_ISL_511201 |
| Portugal/PT1241/2020 | Europe / Portugal |  | 2020-03-31 | EPI_ISL_511202 |
| Portugal/PT1242/2020 | Europe / Portugal |  | 2020-03-31 | EPI_ISL_511203 |
| Portugal/PT1243/2020 | Europe / Portugal |  | 2020-03-31 | EPI_ISL_511204 |
| Portugal/PT1244/2020 | Europe / Portugal |  | 2020-03-31 | EPI_ISL_511205 |
| Portugal/PT1245/2020 | Europe / Portugal |  | 2020-03-31 | EPI_ISL_511206 |
| Portugal/PT1246/2020 | Europe / Portugal |  | 2020-03-31 | EPI_ISL_511207 |
| Portugal/PT1247/2020 | Europe / Portugal |  | 2020-03-31 | EPI_ISL_511208 |
| Portugal/PT1248/2020 | Europe / Portugal |  | 2020-03-31 | EPI_ISL_511209 |
| Portugal/PT1403/2020 | Europe / Portugal |  | 2020-03-31 | EPI_ISL_511362 |
| Portugal/PT1439/2020 | Europe / Portugal |  | 2020-03-31 | EPI_ISL_511398 |
| Portugal/PT1472/2020 | Europe / Portugal |  | 2020-03-31 | EPI_ISL_511431 |
| Portugal/PT1473/2020 | Europe / Portugal |  | 2020-03-31 | EPI_ISL_511432 |
| Portugal/PT1476/2020 | Europe / Portugal | yes | 2020-03-31 | EPI_ISL_511435 |
| Portugal/PT1486/2020 | Europe / Portugal | yes | 2020-03-31 | EPI_ISL_511445 |
| Portugal/PT1492/2020 | Europe / Portugal |  | 2020-03-31 | EPI_ISL_511451 |
| Portugal/PT1497/2020 | Europe / Portugal |  | 2020-03-31 | EPI_ISL_511456 |
| Portugal/PT1511/2020 | Europe / Portugal |  | 2020-03-31 | EPI_ISL_511470 |
| Portugal/PT0245/2020 | Europe / Portugal |  | 2020-04-01 | EPI_ISL_453959 |
| Portugal/PT0431/2020 | Europe / Portugal |  | 2020-04-01 | EPI_ISL_454155 |
| Portugal/PT0432/2020 | Europe / Portugal |  | 2020-04-01 | EPI_ISL_454156 |
| Portugal/PT0433/2020 | Europe / Portugal |  | 2020-04-01 | EPI_ISL_454157 |
| Portugal/PT0487/2020 | Europe / Portugal |  | 2020-04-01 | EPI_ISL_454211 |
| Portugal/PT0769a/2020 | Europe / Portugal |  | 2020-04-01 | EPI_ISL_511028 |
| Portugal/PT1129/2020 | Europe / Portugal |  | 2020-04-01 | EPI_ISL_511676 |
| Portugal/PT1142/2020 | Europe / Portugal |  | 2020-04-01 | EPI_ISL_511689 |
| Portugal/PT1158/2020 | Europe / Portugal |  | 2020-04-01 | EPI_ISL_511705 |
| Portugal/PT1160/2020 | Europe / Portugal |  | 2020-04-01 | EPI_ISL_511707 |
| Portugal/PT0246/2020 | Europe / Portugal | yes | 2020-04-02 | EPI_ISL_453960 |
| Portugal/PT0247/2020 | Europe / Portugal | yes | 2020-04-02 | EPI_ISL_453961 |
| Portugal/PT0248/2020 | Europe / Portugal |  | 2020-04-02 | EPI_ISL_453962 |
| Portugal/PT0249/2020 | Europe / Portugal |  | 2020-04-02 | EPI_ISL_453963 |
| Portugal/PT0250/2020 | Europe / Portugal |  | 2020-04-02 | EPI_ISL_453964 |
| Portugal/PT0251/2020 | Europe / Portugal |  | 2020-04-02 | EPI_ISL_453965 |
| Portugal/PT0252/2020 | Europe / Portugal |  | 2020-04-02 | EPI_ISL_453966 |
| Portugal/PT0671/2020 | Europe / Portugal |  | 2020-04-02 | EPI_ISL_510930 |
| Portugal/PT0773/2020 | Europe / Portugal |  | 2020-04-02 | EPI_ISL_511032 |
| Portugal/PT1159/2020 | Europe / Portugal |  | 2020-04-02 | EPI_ISL_511706 |
| Portugal/PT1161/2020 | Europe / Portugal |  | 2020-04-02 | EPI_ISL_511708 |
| Portugal/PT1162/2020 | Europe / Portugal |  | 2020-04-02 | EPI_ISL_511709 |
| Portugal/PT1163/2020 | Europe / Portugal |  | 2020-04-02 | EPI_ISL_511710 |
| Portugal/PT0253/2020 | Europe / Portugal |  | 2020-04-03 | EPI_ISL_453967 |
| Portugal/PT0254/2020 | Europe / Portugal | yes | 2020-04-03 | EPI_ISL_453968 |
| Portugal/PT0255/2020 | Europe / Portugal | yes | 2020-04-03 | EPI_ISL_453969 |
| Portugal/PT0256/2020 | Europe / Portugal | yes | 2020-04-03 | EPI_ISL_453970 |
| Portugal/PT0257/2020 | Europe / Portugal |  | 2020-04-03 | EPI_ISL_453971 |
| Portugal/PT0258/2020 | Europe / Portugal |  | 2020-04-03 | EPI_ISL_453972 |
| Portugal/PT0259/2020 | Europe / Portugal |  | 2020-04-03 | EPI_ISL_453973 |
| Portugal/PT0260/2020 | Europe / Portugal |  | 2020-04-03 | EPI_ISL_453974 |
| Portugal/PT0261/2020 | Europe / Portugal |  | 2020-04-03 | EPI_ISL_453975 |
| Portugal/PT0262/2020 | Europe / Portugal |  | 2020-04-03 | EPI_ISL_453976 |
| Portugal/PT0451/2020 | Europe / Portugal |  | 2020-04-03 | EPI_ISL_454175 |
| Portugal/PT0547/2020 | Europe / Portugal | yes | 2020-04-03 | EPI_ISL_454271 |
| Portugal/PT0549/2020 | Europe / Portugal |  | 2020-04-03 | EPI_ISL_455627 |
| Portugal/PT0594/2020 | Europe / Portugal |  | 2020-04-03 | EPI_ISL_454317 |
| Portugal/PT0774/2020 | Europe / Portugal |  | 2020-04-03 | EPI_ISL_511033 |
| Portugal/PT0928/2020 | Europe / Portugal |  | 2020-04-03 | EPI_ISL_511185 |
| Portugal/PT0936/2020 | Europe / Portugal |  | 2020-04-03 | EPI_ISL_511487 |
| Portugal/PT0937/2020 | Europe / Portugal |  | 2020-04-03 | EPI_ISL_511488 |
| Portugal/PT0938/2020 | Europe / Portugal |  | 2020-04-03 | EPI_ISL_511489 |
| Portugal/PT0263/2020 | Europe / Portugal |  | 2020-04-04 | EPI_ISL_453977 |
| Portugal/PT0264/2020 | Europe / Portugal |  | 2020-04-04 | EPI_ISL_453978 |
| Portugal/PT0265/2020 | Europe / Portugal | yes | 2020-04-04 | EPI_ISL_453979 |
| Portugal/PT0266/2020 | Europe / Portugal | yes | 2020-04-04 | EPI_ISL_453980 |
| Portugal/PT0267/2020 | Europe / Portugal |  | 2020-04-04 | EPI_ISL_453981 |
| Portugal/PT0268/2020 | Europe / Portugal |  | 2020-04-04 | EPI_ISL_453982 |
| Portugal/PT0269/2020 | Europe / Portugal |  | 2020-04-04 | EPI_ISL_453983 |
| Portugal/PT0452/2020 | Europe / Portugal |  | 2020-04-04 | EPI_ISL_454176 |
| Portugal/PT0453/2020 | Europe / Portugal |  | 2020-04-04 | EPI_ISL_454177 |
| Portugal/PT0662/2020 | Europe / Portugal |  | 2020-04-04 | EPI_ISL_510921 |
| Portugal/PT0663/2020 | Europe / Portugal | yes | 2020-04-04 | EPI_ISL_510922 |
| Portugal/PT0924/2020 | Europe / Portugal |  | 2020-04-04 | EPI_ISL_511181 |
| Portugal/PT1143/2020 | Europe / Portugal |  | 2020-04-04 | EPI_ISL_511690 |
| Portugal/PT0270/2020 | Europe / Portugal |  | 2020-04-05 | EPI_ISL_453984 |
| Portugal/PT0271/2020 | Europe / Portugal |  | 2020-04-05 | EPI_ISL_453985 |
| Portugal/PT0272/2020 | Europe / Portugal |  | 2020-04-05 | EPI_ISL_453986 |
| Portugal/PT0273/2020 | Europe / Portugal |  | 2020-04-05 | EPI_ISL_453987 |
| Portugal/PT0274/2020 | Europe / Portugal |  | 2020-04-05 | EPI_ISL_453988 |
| Portugal/PT0275/2020 | Europe / Portugal |  | 2020-04-05 | EPI_ISL_453989 |
| Portugal/PT0276/2020 | Europe / Portugal |  | 2020-04-05 | EPI_ISL_453990 |
| Portugal/PT0277/2020 | Europe / Portugal |  | 2020-04-05 | EPI_ISL_453991 |
| Portugal/PT0278/2020 | Europe / Portugal |  | 2020-04-05 | EPI_ISL_453992 |
| Portugal/PT0279/2020 | Europe / Portugal |  | 2020-04-05 | EPI_ISL_453993 |
| Portugal/PT0280/2020 | Europe / Portugal |  | 2020-04-05 | EPI_ISL_453994 |
| Portugal/PT0281/2020 | Europe / Portugal |  | 2020-04-05 | EPI_ISL_453995 |
| Portugal/PT0282/2020 | Europe / Portugal |  | 2020-04-05 | EPI_ISL_453996 |
| Portugal/PT1166/2020 | Europe / Portugal |  | 2020-04-05 | EPI_ISL_511712 |
| Portugal/PT1167/2020 | Europe / Portugal |  | 2020-04-05 | EPI_ISL_511713 |
| Portugal/PT1168/2020 | Europe / Portugal |  | 2020-04-05 | EPI_ISL_511714 |
| Portugal/PT1169/2020 | Europe / Portugal |  | 2020-04-05 | EPI_ISL_511715 |
| Portugal/PT0526/2020 | Europe / Portugal |  | 2020-04-06 | EPI_ISL_454250 |
| Portugal/PT0527/2020 | Europe / Portugal | yes | 2020-04-06 | EPI_ISL_454251 |
| Portugal/PT0528/2020 | Europe / Portugal | yes | 2020-04-06 | EPI_ISL_454252 |
| Portugal/PT0529/2020 | Europe / Portugal |  | 2020-04-06 | EPI_ISL_454253 |
| Portugal/PT0601/2020 | Europe / Portugal |  | 2020-04-06 | EPI_ISL_454324 |
| Portugal/PT0628/2020 | Europe / Portugal |  | 2020-04-06 | EPI_ISL_454351 |
| Portugal/PT0629/2020 | Europe / Portugal |  | 2020-04-06 | EPI_ISL_454352 |
| Portugal/PT1164/2020 | Europe / Portugal |  | 2020-04-06 | EPI_ISL_582518 |
| Portugal/PT1165/2020 | Europe / Portugal |  | 2020-04-06 | EPI_ISL_511711 |
| Portugal/PT1402/2020 | Europe / Portugal |  | 2020-04-06 | EPI_ISL_511361 |
| Portugal/PT0531/2020 | Europe / Portugal |  | 2020-04-07 | EPI_ISL_454255 |
| Portugal/PT0532/2020 | Europe / Portugal |  | 2020-04-07 | EPI_ISL_454256 |
| Portugal/PT0533/2020 | Europe / Portugal |  | 2020-04-07 | EPI_ISL_454257 |
| Portugal/PT0534/2020 | Europe / Portugal |  | 2020-04-07 | EPI_ISL_454258 |
| Portugal/PT0535/2020 | Europe / Portugal |  | 2020-04-07 | EPI_ISL_454259 |
| Portugal/PT0536/2020 | Europe / Portugal | yes | 2020-04-07 | EPI_ISL_454260 |
| Portugal/PT0537/2020 | Europe / Portugal | yes | 2020-04-07 | EPI_ISL_454261 |
| Portugal/PT0538/2020 | Europe / Portugal |  | 2020-04-07 | EPI_ISL_454262 |
| Portugal/PT0539/2020 | Europe / Portugal | yes | 2020-04-07 | EPI_ISL_454263 |
| Portugal/PT0551/2020 | Europe / Portugal | yes | 2020-04-07 | EPI_ISL_454274 |
| Portugal/PT0552/2020 | Europe / Portugal | yes | 2020-04-07 | EPI_ISL_454275 |
| Portugal/PT0553/2020 | Europe / Portugal | yes | 2020-04-07 | EPI_ISL_454276 |
| Portugal/PT0554/2020 | Europe / Portugal | yes | 2020-04-07 | EPI_ISL_454277 |
| Portugal/PT0540/2020 | Europe / Portugal |  | 2020-04-08 | EPI_ISL_454264 |
| Portugal/PT0541/2020 | Europe / Portugal |  | 2020-04-08 | EPI_ISL_454265 |
| Portugal/PT0542/2020 | Europe / Portugal |  | 2020-04-08 | EPI_ISL_454266 |
| Portugal/PT0543/2020 | Europe / Portugal |  | 2020-04-08 | EPI_ISL_454267 |
| Portugal/PT0555/2020 | Europe / Portugal | yes | 2020-04-08 | EPI_ISL_454278 |
| Portugal/PT0770a/2020 | Europe / Portugal |  | 2020-04-08 | EPI_ISL_511029 |
| Portugal/PT0939/2020 | Europe / Portugal |  | 2020-04-08 | EPI_ISL_582514 |
| Portugal/PT0940/2020 | Europe / Portugal |  | 2020-04-08 | EPI_ISL_511490 |
| Portugal/PT0941/2020 | Europe / Portugal |  | 2020-04-08 | EPI_ISL_511491 |
| Portugal/PT1144/2020 | Europe / Portugal |  | 2020-04-08 | EPI_ISL_511691 |
| Portugal/PT1145/2020 | Europe / Portugal |  | 2020-04-08 | EPI_ISL_511692 |
| Portugal/PT1146/2020 | Europe / Portugal |  | 2020-04-08 | EPI_ISL_511693 |
| Portugal/PT1147/2020 | Europe / Portugal |  | 2020-04-08 | EPI_ISL_511694 |
| Portugal/PT1148/2020 | Europe / Portugal |  | 2020-04-08 | EPI_ISL_511695 |
| Portugal/PT1149/2020 | Europe / Portugal |  | 2020-04-08 | EPI_ISL_511696 |
| Portugal/PT0544/2020 | Europe / Portugal | yes | 2020-04-09 | EPI_ISL_454268 |
| Portugal/PT0602/2020 | Europe / Portugal |  | 2020-04-09 | EPI_ISL_454325 |
| Portugal/PT0942/2020 | Europe / Portugal |  | 2020-04-09 | EPI_ISL_511492 |
| Portugal/PT0943/2020 | Europe / Portugal |  | 2020-04-09 | EPI_ISL_511493 |
| Portugal/PT0944/2020 | Europe / Portugal |  | 2020-04-09 | EPI_ISL_511494 |
| Portugal/PT1030/2020 | Europe / Portugal |  | 2020-04-09 | EPI_ISL_511577 |
| Portugal/PT0568a/2020 | Europe / Portugal |  | 2020-04-09 | EPI_ISL_511481 |
| Portugal/PT0545/2020 | Europe / Portugal |  | 2020-04-10 | EPI_ISL_454269 |
| Portugal/PT0566/2020 | Europe / Portugal | yes | 2020-04-10 | EPI_ISL_454289 |
| Portugal/PT0945/2020 | Europe / Portugal |  | 2020-04-10 | EPI_ISL_511495 |
| Portugal/PT1130/2020 | Europe / Portugal | yes | 2020-04-10 | EPI_ISL_511677 |
| Portugal/PT1131/2020 | Europe / Portugal |  | 2020-04-10 | EPI_ISL_511678 |
| Portugal/PT0573/2020 | Europe / Portugal |  | 2020-04-11 | EPI_ISL_454296 |
| Portugal/PT0575/2020 | Europe / Portugal |  | 2020-04-11 | EPI_ISL_454298 |
| Portugal/PT0946/2020 | Europe / Portugal |  | 2020-04-11 | EPI_ISL_511496 |
| Portugal/PT0947/2020 | Europe / Portugal |  | 2020-04-11 | EPI_ISL_511497 |
| Portugal/PT1097/2020 | Europe / Portugal |  | 2020-04-11 | EPI_ISL_511644 |
| Portugal/PT0948/2020 | Europe / Portugal |  | 2020-04-12 | EPI_ISL_511498 |
| Portugal/PT0949/2020 | Europe / Portugal |  | 2020-04-12 | EPI_ISL_511499 |
| Portugal/PT0950/2020 | Europe / Portugal |  | 2020-04-12 | EPI_ISL_511500 |
| Portugal/PT0576/2020 | Europe / Portugal |  | 2020-04-13 | EPI_ISL_454299 |
| Portugal/PT0401/2020 | Europe / Portugal | yes | 2020-04-13 | EPI_ISL_454125 |
| Portugal/PT0672/2020 | Europe / Portugal |  | 2020-04-13 | EPI_ISL_510931 |
| Portugal/PT0951/2020 | Europe / Portugal |  | 2020-04-13 | EPI_ISL_511501 |
| Portugal/PT0952/2020 | Europe / Portugal |  | 2020-04-13 | EPI_ISL_511502 |
| Portugal/PT0389a/2020 | Europe / Portugal |  | 2020-04-14 | EPI_ISL_454105 |
| Portugal/PT0390a/2020 | Europe / Portugal |  | 2020-04-14 | EPI_ISL_454108 |
| Portugal/PT0391a/2020 | Europe / Portugal |  | 2020-04-14 | EPI_ISL_454110 |
| Portugal/PT0392/2020 | Europe / Portugal | yes | 2020-04-14 | EPI_ISL_454111 |
| Portugal/PT0393/2020 | Europe / Portugal | yes | 2020-04-14 | EPI_ISL_454112 |
| Portugal/PT0394/2020 | Europe / Portugal | yes | 2020-04-14 | EPI_ISL_454113 |
| Portugal/PT0546/2020 | Europe / Portugal |  | 2020-04-14 | EPI_ISL_454270 |
| Portugal/PT0567/2020 | Europe / Portugal |  | 2020-04-14 | EPI_ISL_454290 |
| Portugal/PT0569/2020 | Europe / Portugal |  | 2020-04-14 | EPI_ISL_454292 |
| Portugal/PT0570/2020 | Europe / Portugal |  | 2020-04-14 | EPI_ISL_454293 |
| Portugal/PT0953/2020 | Europe / Portugal |  | 2020-04-14 | EPI_ISL_511503 |
| Portugal/PT0688/2020 | Europe / Portugal |  | 2020-04-15 | EPI_ISL_510947 |
| Portugal/PT0689/2020 | Europe / Portugal |  | 2020-04-15 | EPI_ISL_510948 |
| Portugal/PT0283a/2020 | Europe / Portugal |  | 2020-04-16 | EPI_ISL_453997 |
| Portugal/PT0454/2020 | Europe / Portugal |  | 2020-04-17 | EPI_ISL_454178 |
| Portugal/PT0455/2020 | Europe / Portugal |  | 2020-04-17 | EPI_ISL_454179 |
| Portugal/PT0459/2020 | Europe / Portugal |  | 2020-04-17 | EPI_ISL_454183 |
| Portugal/PT0460/2020 | Europe / Portugal |  | 2020-04-17 | EPI_ISL_454184 |
| Portugal/PT0461/2020 | Europe / Portugal |  | 2020-04-17 | EPI_ISL_454185 |
| Portugal/PT0462/2020 | Europe / Portugal |  | 2020-04-17 | EPI_ISL_454186 |
| Portugal/PT0463/2020 | Europe / Portugal |  | 2020-04-17 | EPI_ISL_454187 |
| Portugal/PT0464/2020 | Europe / Portugal |  | 2020-04-17 | EPI_ISL_454188 |
| Portugal/PT0674/2020 | Europe / Portugal |  | 2020-04-17 | EPI_ISL_510933 |
| Portugal/PT0690/2020 | Europe / Portugal |  | 2020-04-17 | EPI_ISL_510949 |
| Portugal/PT0954/2020 | Europe / Portugal |  | 2020-04-17 | EPI_ISL_582515 |
| Portugal/PT0958/2020 | Europe / Portugal |  | 2020-04-17 | EPI_ISL_511507 |
| Portugal/PT0395a/2020 | Europe / Portugal | yes | 2020-04-18 | EPI_ISL_454114 |
| Portugal/PT0396a/2020 | Europe / Portugal |  | 2020-04-18 | EPI_ISL_454117 |
| Portugal/PT0456/2020 | Europe / Portugal |  | 2020-04-18 | EPI_ISL_454180 |
| Portugal/PT0457/2020 | Europe / Portugal |  | 2020-04-18 | EPI_ISL_454181 |
| Portugal/PT0465/2020 | Europe / Portugal |  | 2020-04-18 | EPI_ISL_454189 |
| Portugal/PT0466/2020 | Europe / Portugal |  | 2020-04-18 | EPI_ISL_454190 |
| Portugal/PT0467/2020 | Europe / Portugal |  | 2020-04-18 | EPI_ISL_454191 |
| Portugal/PT0468/2020 | Europe / Portugal |  | 2020-04-18 | EPI_ISL_454192 |
| Portugal/PT0469/2020 | Europe / Portugal |  | 2020-04-18 | EPI_ISL_454193 |
| Portugal/PT0470a/2020 | Europe / Portugal |  | 2020-04-18 | EPI_ISL_454194 |
| Portugal/PT0471/2020 | Europe / Portugal |  | 2020-04-18 | EPI_ISL_454195 |
| Portugal/PT0472/2020 | Europe / Portugal |  | 2020-04-18 | EPI_ISL_454196 |
| Portugal/PT0397a/2020 | Europe / Portugal | yes | 2020-04-19 | EPI_ISL_454119 |
| Portugal/PT0398/2020 | Europe / Portugal | yes | 2020-04-19 | EPI_ISL_454121 |
| Portugal/PT0399a/2020 | Europe / Portugal |  | 2020-04-19 | EPI_ISL_454122 |
| Portugal/PT0458/2020 | Europe / Portugal |  | 2020-04-19 | EPI_ISL_454182 |
| Portugal/PT0691/2020 | Europe / Portugal |  | 2020-04-19 | EPI_ISL_510950 |
| Portugal/PT0955/2020 | Europe / Portugal |  | 2020-04-19 | EPI_ISL_511504 |
| Portugal/PT0957/2020 | Europe / Portugal |  | 2020-04-19 | EPI_ISL_511506 |
| Portugal/PT0284/2020 | Europe / Portugal |  | 2020-04-20 | EPI_ISL_453999 |
| Portugal/PT0400/2020 | Europe / Portugal | yes | 2020-04-20 | EPI_ISL_454124 |
| Portugal/PT0556/2020 | Europe / Portugal |  | 2020-04-20 | EPI_ISL_454279 |
| Portugal/PT0557/2020 | Europe / Portugal |  | 2020-04-20 | EPI_ISL_454280 |
| Portugal/PT0956/2020 | Europe / Portugal |  | 2020-04-20 | EPI_ISL_511505 |
| Portugal/PT0558/2020 | Europe / Portugal |  | 2020-04-21 | EPI_ISL_454281 |
| Portugal/PT0402/2020 | Europe / Portugal | yes | 2020-04-22 | EPI_ISL_454126 |
| Portugal/PT0559/2020 | Europe / Portugal |  | 2020-04-22 | EPI_ISL_454282 |
| Portugal/PT0560/2020 | Europe / Portugal |  | 2020-04-22 | EPI_ISL_454283 |
| Portugal/PT0692/2020 | Europe / Portugal |  | 2020-04-23 | EPI_ISL_510951 |
| Portugal/PT0473/2020 | Europe / Portugal |  | 2020-04-24 | EPI_ISL_454197 |
| Portugal/PT0474/2020 | Europe / Portugal |  | 2020-04-24 | EPI_ISL_454198 |
| Portugal/PT0475/2020 | Europe / Portugal |  | 2020-04-24 | EPI_ISL_454199 |
| Portugal/PT0476/2020 | Europe / Portugal |  | 2020-04-24 | EPI_ISL_454200 |
| Portugal/PT0581/2020 | Europe / Portugal |  | 2020-04-24 | EPI_ISL_454304 |
| Portugal/PT0880/2020 | Europe / Portugal |  | 2020-04-24 | EPI_ISL_511139 |
| Portugal/PT1150/2020 | Europe / Portugal |  | 2020-04-24 | EPI_ISL_511697 |
| Portugal/PT0675/2020 | Europe / Portugal |  | 2020-04-25 | EPI_ISL_510934 |
| Portugal/PT0693/2020 | Europe / Portugal |  | 2020-04-25 | EPI_ISL_510952 |
| Portugal/PT0925/2020 | Europe / Portugal |  | 2020-04-25 | EPI_ISL_511182 |
| Portugal/PT0926/2020 | Europe / Portugal |  | 2020-04-25 | EPI_ISL_511183 |
| Portugal/PT1151/2020 | Europe / Portugal |  | 2020-04-25 | EPI_ISL_511698 |
| Portugal/PT1152/2020 | Europe / Portugal |  | 2020-04-25 | EPI_ISL_511699 |
| Portugal/PT1153/2020 | Europe / Portugal |  | 2020-04-25 | EPI_ISL_511700 |
| Portugal/PT1154/2020 | Europe / Portugal |  | 2020-04-25 | EPI_ISL_511701 |
| Portugal/PT1155/2020 | Europe / Portugal |  | 2020-04-25 | EPI_ISL_511702 |
| Portugal/PT1156/2020 | Europe / Portugal |  | 2020-04-25 | EPI_ISL_511703 |
| Portugal/PT0477/2020 | Europe / Portugal |  | 2020-04-26 | EPI_ISL_454201 |
| Portugal/PT0881/2020 | Europe / Portugal |  | 2020-04-26 | EPI_ISL_511140 |
| Portugal/PT0927/2020 | Europe / Portugal |  | 2020-04-26 | EPI_ISL_511184 |
| Portugal/PT1157/2020 | Europe / Portugal |  | 2020-04-26 | EPI_ISL_511704 |
| Portugal/PT0489/2020 | Europe / Portugal |  | 2020-04-27 | EPI_ISL_454213 |
| Portugal/PT0561/2020 | Europe / Portugal |  | 2020-04-27 | EPI_ISL_454284 |
| Portugal/PT0562/2020 | Europe / Portugal | yes | 2020-04-27 | EPI_ISL_454285 |
| Portugal/PT0490/2020 | Europe / Portugal |  | 2020-04-28 | EPI_ISL_454214 |
| Portugal/PT0844/2020 | Europe / Portugal |  | 2020-04-28 | EPI_ISL_511103 |
| Portugal/PT0491/2020 | Europe / Portugal |  | 2020-04-29 | EPI_ISL_454215 |
| Portugal/PT0492/2020 | Europe / Portugal |  | 2020-04-29 | EPI_ISL_454216 |
| Portugal/PT0493/2020 | Europe / Portugal |  | 2020-04-29 | EPI_ISL_454217 |
| Portugal/PT0494/2020 | Europe / Portugal |  | 2020-04-29 | EPI_ISL_454218 |
| Portugal/PT0959/2020 | Europe / Portugal |  | 2020-04-29 | EPI_ISL_511508 |
| Portugal/PT0495/2020 | Europe / Portugal |  | 2020-04-30 | EPI_ISL_454219 |
| Portugal/PT0496/2020 | Europe / Portugal |  | 2020-04-30 | EPI_ISL_454220 |
| Portugal/PT0847/2020 | Europe / Portugal |  | 2020-04-30 | EPI_ISL_511106 |
| Portugal/PT0882/2020 | Europe / Portugal |  | 2020-04-30 | EPI_ISL_511141 |
| Portugal/PT0497/2020 | Europe / Portugal |  | 2020-05-01 | EPI_ISL_454221 |
| Portugal/PT0548/2020 | Europe / Portugal | yes | 2020-05-02 | EPI_ISL_454272 |
| Portugal/PT0563/2020 | Europe / Portugal | yes | 2020-05-02 | EPI_ISL_454286 |
| Portugal/PT0593/2020 | Europe / Portugal |  | 2020-05-04 | EPI_ISL_454316 |
| Portugal/PT0550/2020 | Europe / Portugal | yes | 2020-05-05 | EPI_ISL_454273 |
| Portugal/PT0771/2020 | Europe / Portugal |  | 2020-05-05 | EPI_ISL_511030 |
| Portugal/PT0846/2020 | Europe / Portugal |  | 2020-05-05 | EPI_ISL_511105 |
| Portugal/PT0884/2020 | Europe / Portugal |  | 2020-05-05 | EPI_ISL_511142 |
| Portugal/PT0845/2020 | Europe / Portugal |  | 2020-05-06 | EPI_ISL_511104 |
| Portugal/PT0885/2020 | Europe / Portugal |  | 2020-05-06 | EPI_ISL_511143 |
| Portugal/PT1136/2020 | Europe / Portugal |  | 2020-05-06 | EPI_ISL_511683 |
| Portugal/PT1137/2020 | Europe / Portugal |  | 2020-05-06 | EPI_ISL_511684 |
| Portugal/PT0772/2020 | Europe / Portugal |  | 2020-05-17 | EPI_ISL_511031 |
| Portugal/PT1031/2020 | Europe / Portugal |  | 2020-05-21 | EPI_ISL_511578 |
| Portugal/PT1032a/2020 | Europe / Portugal |  | 2020-06-05 | EPI_ISL_511579 |
| Portugal/PT1033/2020 | Europe / Portugal |  | 2020-06-06 | EPI_ISL_511580 |

| **Table S2. List of SARS-CoV-2 Spike amino acid sequences with mutations in the 839 site available at GISAID, as of July 23^rd^, 2020.** | | | | | | | | |
| --- | --- | --- | --- | --- | --- | --- | --- | --- |
|  |  |  |  |  |  |  |  |  |
| **virus ID** | **Mutation in Spike amino acid D839** | **Spike D614G background** | **Nexstrain clade^a^** | **GISAID Accession ID^b^** | **date of colllection** | **continent** | **country** | **region** |
| Italy/PV-5314-N/2020 | D839Y | G614 | 20A | EPI_ISL_451307 | 2020-02-21 | Europe | Italy | Lombardy |
| Netherlands/Diemen_1363454/2020 | D839E | G614 | 20B | EPI_ISL_413570 | 2020-02-28 | Europe | Netherlands | Netherlands |
| England/20099107406/2020 | D839Y | G614 | 20A | EPI_ISL_415131 | 2020-02-29 | Europe | United Kingdom | England |
| England/20102000506/2020 | D839Y | G614 | 20A | EPI_ISL_415147 | 2020-03-01 | Europe | United Kingdom | England |
| England/201061455/2020 | D839Y | G614 | 20A | EPI_ISL_464413 | 2020-03-01 | Europe | United Kingdom | England |
| England/CAMB-847A5/2020 | D839Y | G614 | 20A | EPI_ISL_440513 | 2020-03-01 | Europe | United Kingdom | England |
| Iceland/14/2020 | D839Y | G614 | 20A | EPI_ISL_417736 | 2020-03-01 | Europe | Iceland | Reykjavik |
| England/20102132304/2020 | D839Y | G614 | 20A | EPI_ISL_464290 | 2020-03-02 | Europe | United Kingdom | England |
| England/20104009002/2020 | D839Y | G614 | 20A | EPI_ISL_417233 | 2020-03-02 | Europe | United Kingdom | England |
| Iceland/20/2020 | D839Y | G614 | 20A | EPI_ISL_417695 | 2020-03-02 | Europe | Iceland | Reykjavik |
| England/20108003302/2020 | D839Y | G614 | 20A | EPI_ISL_417248 | 2020-03-03 | Europe | United Kingdom | England |
| England/20108004803/2020 | D839Y | G614 | 20A | EPI_ISL_417251 | 2020-03-03 | Europe | United Kingdom | England |
| England/201080050/2020 | D839Y | G614 | 20A | EPI_ISL_464442 | 2020-03-03 | Europe | United Kingdom | England |
| England/BRIS-12177C/2020 | D839Y | G614 | 20A | EPI_ISL_440256 | 2020-03-03 | Europe | United Kingdom | England |
| England/BRIS-12178B/2020 | D839Y | G614 | 20A | EPI_ISL_440212 | 2020-03-03 | Europe | United Kingdom | England |
| England/BRIS-12F5A1/2020 | D839Y | G614 | 20A | EPI_ISL_488219 | 2020-03-03 | Europe | United Kingdom | England |
| England/201060017/2020 | D839Y | G614 | 20A | EPI_ISL_464362 | 2020-03-04 | Europe | United Kingdom | England |
| England/201080076/2020 | D839Y | G614 | 20A | EPI_ISL_464459 | 2020-03-04 | Europe | United Kingdom | England |
| England/20110000706/2020 | D839Y | G614 | 20A | EPI_ISL_417302 | 2020-03-05 | Europe | United Kingdom | England |
| England/20110000606/2020 | D839Y | G614 | 20A | EPI_ISL_417301 | 2020-03-06 | Europe | United Kingdom | England |
| England/20110000906/2020 | D839Y | G614 | 20A | EPI_ISL_417304 | 2020-03-06 | Europe | United Kingdom | England |
| England/BRIS-12F617/2020 | D839Y | G614 | 20A | EPI_ISL_488253 | 2020-03-06 | Europe | United Kingdom | England |
| England/20109007304/2020 | D839Y | G614 | 20A | EPI_ISL_464527 | 2020-03-07 | Europe | United Kingdom | England |
| England/201090513/2020 | D839Y | G614 | 20A | EPI_ISL_464577 | 2020-03-07 | Europe | United Kingdom | England |
| Iceland/69/2020 | D839Y | G614 | 20A | EPI_ISL_417749 | 2020-03-08 | Europe | Iceland | Reykjavik |
| England/201120242/2020 | D839Y | G614 | 20A | EPI_ISL_464672 | 2020-03-09 | Europe | United Kingdom | England |
| England/201140338/2020 | D839Y | G614 | 20A | EPI_ISL_464724 | 2020-03-09 | Europe | United Kingdom | England |
| England/BRIS-12F750/2020 | D839Y | G614 | 20A | EPI_ISL_488349 | 2020-03-09 | Europe | United Kingdom | England |
| Iceland/103/2020 | D839Y | G614 | 20A | EPI_ISL_417698 | 2020-03-10 | Europe | Iceland | Reykjavik |
| England/201140062/2020 | D839Y | G614 | 20A | EPI_ISL_464713 | 2020-03-11 | Europe | United Kingdom | England |
| England/20119002104/2020 | D839Y | G614 | 20A | EPI_ISL_465007 | 2020-03-12 | Europe | United Kingdom | England |
| England/CAMB-75516/2020 | D839Y | G614 | 20A | EPI_ISL_425401 | 2020-03-13 | Europe | United Kingdom | England |
| Georgia/Tb/2020 | D839Y | G614 | 20A | EPI_ISL_416482 | 2020-03-13 | Asia | Georgia | Tbilisi |
| Netherlands/ZuidHolland_44/2020 | D839Y | G614 | 20A | EPI_ISL_422916 | 2020-03-19 | Europe | Netherlands | South Holland |
| Netherlands/ZuidHolland_45/2020 | D839Y | G614 | 20A | EPI_ISL_422917 | 2020-03-19 | Europe | Netherlands | South Holland |
| Netherlands/ZuidHolland_52/2020 | D839Y | G614 | 20A | EPI_ISL_422920 | 2020-03-19 | Europe | Netherlands | South Holland |
| Poland/1105973/2020 | D839Y | G614 | 20A | EPI_ISL_428234 | 2020-03-19 | Europe | Poland | Pomorskie |
| NewZealand/20VR1278/2020 | D839Y | G614 | 20A | EPI_ISL_456203 | 2020-03-20 | Oceania | New Zealand | Auckland |
| Netherlands/NA_165/2020 | D839Y | G614 | 20A | EPI_ISL_422700 | 2020-03-21 | Europe | Netherlands | Netherlands |
| Netherlands/NA_173/2020 | D839Y | G614 | 20A | EPI_ISL_422708 | 2020-03-22 | Europe | Netherlands | Netherlands |
| Netherlands/Utrecht_10022/2020 | D839Y | G614 | 20A | EPI_ISL_454771 | 2020-03-23 | Europe | Netherlands | Utrecht |
| England/NORT-281D2C/2020 | D839G | D614 | 19A | EPI_ISL_478483 | 2020-03-24 | Europe | United Kingdom | England |
| Netherlands/ZuidHolland_73/2020 | D839Y | G614 | 20A | EPI_ISL_422941 | 2020-03-24 | Europe | Netherlands | South Holland |
| Austria/CeMM0397/2020 | D839Y | G614 | 20A | EPI_ISL_475834 | 2020-03-25 | Europe | Austria | Austria |
| Wales/PHWC-25EDE/2020 | D839N | D614 | 19A | EPI_ISL_422317 | 2020-03-25 | Europe | United Kingdom | Wales |
| Switzerland/100799/2020 | D839Y | G614 | 20A | EPI_ISL_451749 | 2020-03-25 | Europe | Switzerland | Uri |
| Switzerland/100804/2020 | D839Y | G614 | 20A | EPI_ISL_451754 | 2020-03-25 | Europe | Switzerland | Uri |
| USA/TX-HMH0273/2020 | D839Y | G614 | 20A | EPI_ISL_434901 | 2020-03-26 | America | USA | Texas |
| Switzerland/110014_159_F6/2020 | D839Y | G614 | 20A | EPI_ISL_486454 | 2020-04-01 | Europe | Switzerland | Uri |
| Netherlands/NA_308/2020 | D839Y | G614 | 20A | EPI_ISL_422617 | 2020-04-02 | Europe | Netherlands | Netherlands |
| NewZealand/20VR2058/2020 | D839Y | G614 | 20A | EPI_ISL_456352 | 2020-04-05 | Oceania | New Zealand | Canterbury |
| NewZealand/20VR2065/2020 | D839Y | G614 | 20A | EPI_ISL_456356 | 2020-04-06 | Oceania | New Zealand | Canterbury |
| NewZealand/20VR2067/2020 | D839Y | G614 | 20A | EPI_ISL_456358 | 2020-04-06 | Oceania | New Zealand | Canterbury |
| England/EXET-136DAF/2020 | D839Y | G614 | 20A | EPI_ISL_471924 | 2020-04-07 | Europe | United Kingdom | England |
| NewZealand/20VR2075/2020 | D839Y | G614 | 20A | EPI_ISL_456362 | 2020-04-09 | Oceania | New Zealand | Canterbury |
| NewZealand/20VR2076/2020 | D839Y | G614 | 20A | EPI_ISL_456363 | 2020-04-09 | Oceania | New Zealand | Canterbury |
| NewZealand/20VR2077/2020 | D839Y | G614 | 20A | EPI_ISL_456364 | 2020-04-09 | Oceania | New Zealand | Canterbury |
| NewZealand/20VR2078/2020 | D839Y | G614 | 20A | EPI_ISL_456365 | 2020-04-09 | Oceania | New Zealand | Canterbury |
| NewZealand/20VR2079/2020 | D839Y | G614 | 20A | EPI_ISL_456366 | 2020-04-10 | Oceania | New Zealand | Canterbury |
| NewZealand/20VR2093/2020 | D839Y | G614 | 20A | EPI_ISL_456374 | 2020-04-10 | Oceania | New Zealand | Canterbury |
| Wales/PHWC-2B579/2020 | D839Y | G614 | 20A | EPI_ISL_445727 | 2020-04-10 | Europe | United Kingdom | Wales |
| NewZealand/20VR2081/2020 | D839Y | G614 | 20A | EPI_ISL_456367 | 2020-04-11 | Oceania | New Zealand | Canterbury |
| NewZealand/20VR2085/2020 | D839Y | G614 | 20A | EPI_ISL_456369 | 2020-04-12 | Oceania | New Zealand | Canterbury |
| NewZealand/20VR2088/2020 | D839Y | G614 | 20A | EPI_ISL_456371 | 2020-04-12 | Oceania | New Zealand | Canterbury |
| Wales/PHWC-31516/2020 | D839Y | G614 | 20A | EPI_ISL_446456 | 2020-04-12 | Europe | United Kingdom | Wales |
| Wales/PHWC-31B9C/2020 | D839Y | G614 | 20A | EPI_ISL_446537 | 2020-04-12 | Europe | United Kingdom | Wales |
| England/BRIS-12F26E/2020 | D839Y | G614 | 20A | EPI_ISL_488372 | 2020-04-13 | Europe | United Kingdom | England |
| England/BRIS-12F4D1/2020 | D839Y | G614 | 20A | EPI_ISL_488265 | 2020-04-13 | Europe | United Kingdom | England |
| England/BRIS-12F4E0/2020 | D839Y | G614 | 20A | EPI_ISL_488309 | 2020-04-13 | Europe | United Kingdom | England |
| England/EXET-135915/2020 | D839Y | G614 | 20A | EPI_ISL_457116 | 2020-04-13 | Europe | United Kingdom | England |
| Netherlands/ZuidHolland_141/2020 | D839Y | G614 | 20A | EPI_ISL_461314 | 2020-04-13 | Europe | Netherlands | South Holland |
| NewZealand/20VR2089/2020 | D839Y | G614 | 20A | EPI_ISL_456372 | 2020-04-13 | Oceania | New Zealand | Canterbury |
| England/EXET-135863/2020 | D839Y | G614 | 20A | EPI_ISL_457108 | 2020-04-15 | Europe | United Kingdom | England |
| England/EXET-1358BE/2020 | D839Y | G614 | 20A | EPI_ISL_457112 | 2020-04-15 | Europe | United Kingdom | England |
| Wales/PHWC-3415B/2020 | D839Y | G614 | 20A | EPI_ISL_446877 | 2020-04-16 | Europe | United Kingdom | Wales |
| Wales/PHWC-3416A/2020 | D839Y | G614 | 20A | EPI_ISL_446878 | 2020-04-16 | Europe | United Kingdom | Wales |
| Wales/PHWC-34461/2020 | D839Y | G614 | 20A | EPI_ISL_446924 | 2020-04-16 | Europe | United Kingdom | Wales |
| Wales/PHWC-34470/2020 | D839Y | G614 | 20A | EPI_ISL_446925 | 2020-04-16 | Europe | United Kingdom | Wales |
| Wales/PHWC-15C329/2020 | D839Y | G614 | 20A | EPI_ISL_472471 | 2020-04-18 | Europe | United Kingdom | Wales |
| Wales/PHWC-35A6E/2020 | D839Y | G614 | 20A | EPI_ISL_474518 | 2020-04-24 | Europe | United Kingdom | Wales |
| Wales/PHWC-35B01/2020 | D839Y | G614 | 20C | EPI_ISL_474528 | 2020-04-24 | Europe | United Kingdom | Wales |
| Wales/PHWC-163E00/2020 | D839Y | G614 | 20A | EPI_ISL_474087 | 2020-04-25 | Europe | United Kingdom | Wales |
| England/TBSD-2488DC3/2020 | D839Y | G614 | 20A | EPI_ISL_482060 | 2020-04-26 | Europe | United Kingdom | England |
| Wales/PHWC-35F41/2020 | D839Y | G614 | 20A | EPI_ISL_474594 | 2020-04-27 | Europe | United Kingdom | Wales |
| England/TBSD-2488E57/2020 | D839Y | G614 | 20A | EPI_ISL_482059 | 2020-04-28 | Europe | United Kingdom | England |
| Switzerland/120232/2020 | D839Y | G614 | 20A | EPI_ISL_468293 | 2020-05-01 | Europe | Switzerland | Uri |
| England/BRIS-12D87D/2020 | D839Y | G614 | 20A | EPI_ISL_481951 | 2020-05-06 | Europe | United Kingdom | England |
| England/NORT-295AF1/2020 | D839G | D614 | 19A | EPI_ISL_472288 | 2020-05-08 | Europe | United Kingdom | England |
| Wales/PHWC-16095D/2020 | D839Y | G614 | 20A | EPI_ISL_472997 | 2020-05-18 | Europe | United Kingdom | Wales |
| Wales/PHWC-160B84/2020 | D839Y | G614 | 20A | EPI_ISL_473022 | 2020-05-21 | Europe | United Kingdom | Wales |
| India/InStem_NCBS_0020/2020 | D839Y | G614 | 20A | EPI_ISL_477222 | 2020-06-11 | Asia | India | Karnataka |
| India/InStem_NCBS_0021/2020 | D839Y | G614 | 20A | EPI_ISL_477223 | 2020-06-11 | Asia | India | Karnataka |
| India/InStem_NCBS_0081/2020 | D839Y | G614 | 20A | EPI_ISL_479752 | 2020-06-17 | Asia | India | Karnataka |
| Australia/VIC2137/2020 | D839N | G614 | 20A | EPI_ISL_480738 | 2020-06-20 | Oceania | Australia | Victoria |
| England/OXON-B0667/2020 | D839Y | G614 | 20A | EPI_ISL_478896 | 2020 | Europe | United Kingdom | England |
| India/InStem_NCBS_0089/2020 | D839Y | G614 | 20A | EPI_ISL_486838 | 2020 | Asia | India | Karnataka |
| Estonia/ChVir1985/2020 | D839Y | G614 | 20A | EPI_ISL_420067 | 2020-03 | Europe | Estonia | Estonia |
|  |  |  |  |  |  |  |  |  |
| ^a^ Nextstrain clade was determined using Nextclade online tool (https://clades.nextstrain.org/). | | | | | |  |  |  |
| ^b^ Acknowledgement regarding sequences from GISAID’s EpiFlu™ Database: We acknowledge the authors, originating and submitting laboratories of the sequences from GISAID’s Database on which this research is based. | | | | | | | | |
|  |  |  |  |  |  |  |  |  |
